# Meta-Analysis Comparing Zero-Profile Spacer and Anterior Plate in Anterior Cervical Fusion

**DOI:** 10.1371/journal.pone.0130223

**Published:** 2015-06-11

**Authors:** Jun Dong, Meng Lu, Teng Lu, Baobao Liang, Junkui Xu, Jun Zhou, Hongjun Lv, Jie Qin, Xuan Cai, Sihua Huang, Haopeng Li, Dong Wang, Xijing He

**Affiliations:** 1 Department of Orthopaedics, Second Affiliated Hospital of Xi’an Jiaotong University, Xi’an, Shaanxi Province 710004, China; 2 Department of Plastic Surgery, Second Affiliated Hospital of Xi’an Jiaotong University, Xi’an, Shaanxi Province 710004, China; 3 Department of Orthopaedics, Xi’an Honghui hospital of Xi’an Jiaotong University, Xi’an, Shaanxi Province 710054, China; 4 Department of Dermatology, Second Affiliated Hospital of Xi’an Jiaotong University, Xi’an, Shaanxi Province 710004, China; 5 Department of Endocrinology, First Affiliated Hospital of Xi’an Jiaotong University, Xi’an, Shaanxi Province 710061, China; University of Pennsylvania, UNITED STATES

## Abstract

**Background:**

Anterior plate fusion is an effective procedure for the treatment of cervical spinal diseases but is accompanied by a high incidence of postoperative dysphagia. A zero profile (Zero-P) spacer is increasingly being used to reduce postoperative dysphagia and other potential complications associated with surgical intervention. Studies comparing the Zero-P spacer and anterior plate have reported conflicting results.

**Methodology:**

A meta-analysis was conducted to compare the safety, efficacy, radiological outcomes and complications associated with the use of a Zero-P spacer versus an anterior plate in anterior cervical spine fusion for the treatment of cervical spinal disease. We comprehensively searched PubMed, Embase, the Cochrane Library and other databases and performed a meta-analysis of all randomized controlled trials (RCTs) and prospective or retrospective comparative studies assessing the two techniques.

**Results:**

Ten studies enrolling 719 cervical spondylosis patients were included. The pooled data showed significant differences in the operation time [SMD = –0.58 (95% CI = −0.77 to 0.40, p < 0.01)] and blood loss [SMD = −0.40, 95% CI (−0.59 to –0.21), p < 0.01] between the two groups. Compared to the anterior plate group, the Zero-P group exhibited a significantly improved JOA score and reduced NDI and VAS. However, anterior plate fusion had greater postoperative segmental and cervical Cobb’s angles than the Zero-P group at the last follow-up. The fusion rate in the two groups was similar. More importantly, the Zero-P group had a lower incidence of earlier and later postoperative dysphagia.

**Conclusions:**

Compared to anterior plate fusion, Zero-P is a safer and effective procedure, with a similar fusion rate and lower incidence of earlier and later postoperative dysphagia. However, the results of this meta-analysis should be accepted with caution due to the limitations of the study. Further evaluation and large-sample RCTs are required to confirm and update the results of this study.

## Introduction

Cervical spondylosis is a major cause of spinal cord dysfunction[1]. Patients suffering from this disease are often treated with anterior cervical discectomy and fusion (ACDF), which has been regarded as the gold-standard procedure in recent decades[2]. This surgical procedure usually fuses two or more cervical vertebrae using an anterior plate combined with vertebral screws or by using an interbody fusion device or bone graft after decompression by discectomy. The procedure can provide spine segments with a stable biomechanical environment at the operative level. Although ACDF has been reported to have a high fusion rate of 92.8%[3], as well as maximum restoration of physiological sagittal alignment[4], it is also accompanied by various complications[5], one of which is dysphagia. The reported incidence rate of postoperative dysphagia at the early stage varies from 1% to 71%[6–8], and its one-year prevalence rate plateau varies from 12.5% to 21%[9, 10]. The high profile of the plate is one of the notable drawbacks leading to postoperative dysphagia[11].

An anchored spacer, a zero-profile interbody fusion device (Zero-P), has been developed to reduce the potential risk of complications after anterior cervical fusion. Biomechanical testing of Zero-P has demonstrated similar stability when compared with the anterior plate[12, 13]. This device contains an interbody spacer and a rigid, very low-profile plate for the screw fixation in the interbody interval instead of the anterior space of the cervical vertebral body. Although theoretically, the width of the pre-vertebral soft tissue could be decreased by the Zero-P device, studies have reported that the postoperative width of the pre-vertebral soft tissue did not contribute to postoperative dysphagia[14, 15]. Though Zero-P requires a smaller surgical area, it takes time to dissect the soft tissues, paravertebral muscle and anterior longitudinal ligament in order to expose the intervertebral space for its implantation.

A randomized controlled study reported that the difference in the incidence of dysphagia in patients who underwent Zero-P fusion was not significant compared with the patients in the anterior plate fusion group[16]. However, the number of patients in Nemoto et al.’s study was small[16]. Several *in vivo* studies showed that Zero-P did decrease the operative time, volume of blood loss and incidence rate of postoperative dysphagia versus anterior plate fusion[17–20]. To obtain a reliable conclusion, we performed a meta-analysis to compare the operative time, volume of blood loss, dysphagia rate, Cobb’s angle and other evaluation indexes between Zero-P fusion and anterior plate fusion. To the best of our knowledge, this study is the first meta-analysis comparing Zero-P and anterior plate fusion.

## Materials and Methods

Our study was conducted under the guidelines from the Review Manager handbook (version: 5.3.3) from Cochrane Collaboration and was performed on the basis of preferred reporting items for systematic reviews and meta-analysis (PRISMA)[21]. The PRISMA checklist of our study is shown in **S1 Table**. The protocol was prepared in advance. The search strategy, inclusion and exclusion criteria, data extraction and quality assessment, and statistical analysis were performed as follows.

### Search strategy

Electronic databases, including MEDLINE, Embase, Cochrane Library, ISI Web of Knowledge (all databases), and CBM (China Biology Medicine) were searched for relevant reports published up to October 31, 2014. The keywords used for our searches included ‘cervica* AND spin*,’ ‘anterior cervical fusion,’ ‘anterior plate,’ ‘anterior cervical discectomy and fusion,’ ‘ACDF,’ ‘interbody fusion,’ ‘low profile,’ ‘zero profile,’ ‘zero-p,’ ‘anchored fusion,’ ‘anchored spacer device,’ and ‘stand alone.’ The conjunctions “AND” and “OR” were used during the literature retrieval. The language was not restricted. The retrieval processes are shown in **S1–S5 Figs**. References from the retrieved articles were checked manually for additional studies.

### Inclusion and exclusion criteria

Two authors (Jun D. and Meng L.) reviewed the articles, including randomized controlled trials (RCTs) and retrospective or prospective studies, in detail. The inclusion criteria for this study were as follows: (1) all patients with cervical spondylosis and who have undergone an anterior fusion procedure; (2) studies involving two cervical fusion groups: one with a Zero-P device and the other with an anterior fusion plate; and (3) a follow-up time of no less than 12 months. The following articles were excluded: (1) meeting abstracts, review articles, editorial comments, letters, technical reports, case reports, biomechanical studies and animal experiments; (2) studies that did not meet the inclusion criteria; (3) articles considered as duplicate publications; and (4) articles for which the full text was not available.

### Data extraction and quality assessment

Two authors (Teng L. and Baobao L.) independently extracted data from each included study. The primary end points were the dysphagia rates within six weeks and at last follow-up. The secondary end points were the operation time, blood loss, preoperative and postoperative Japanese Orthopedic Association (JOA) score, preoperative and postoperative neck disability index (NDI), preoperative and postoperative visual analog scale (VAS), postoperative segmental Cobb’s angle, postoperative cervical Cobb’s angle (the angle between the inferior endplate of C_2_ and the inferior endplate of C_7_) and fusion rate. The following data were recorded: (1) basic characteristics: first author, publication year, study design, sample size, etc.; (2) intraoperative outcomes: operation time and volume of blood loss; (3) clinical outcomes: JOA score, VAS score and NDI for neck pain; (4) radiological outcomes: cervical Cobb’s angle, segment Cobb’s angle and fusion rate; and (5) complications: dysphagia rate within six weeks and at the last follow-up. If the data were available in figures rather than in a table, the software GetData Graph Digitizer (S. Fedorov, version: 2.24) was used to obtain the original (x, y) data from the graphs. If the data [mean or standard deviation (SD)] needed to be combined from multiple groups into one group, a formula for data combining was used (**S2 Table**). Two authors independently assessed the methodological quality of each study. The RCTs were evaluated according to a method described by Chalmers et al.[22] (**S3 Table**) Retrospective and prospective studies underwent quality assessment using the modified MINORS criteria[23] (**S4 Table**). Corresponding authors were contacted for further details. The authors resolved any disagreements through discussions with the senior author (Xijing H.).

### Statistical analysis

Meta-analysis was performed using the Mantel-Haenszel method with Review Manager software (RevMan version 5.3.3, Cochrane Collaboration, Germany). The mean difference (MD)/standardized mean difference (SMD) and 95% confidence interval (CI) were used for continuous data calculation. Relative risk (RR) and 95% CI were used for dichotomous data. Heterogeneity was assessed using I^2^ and the result of the chi-squared test. Significant heterogeneity was assumed when the p-value was less than 0.1 or the I^2^ value was greater than 50%. Fixed-effects modeling was applied in the absence of heterogeneity. Subgroup or sensitivity analysis was performed when the cause of heterogeneity was ascertained. However, if the heterogeneity could not be eliminated, random-effects modeling was used for the combined analysis of the studies.

## Results

### Literature results and bias assessment

**Fig 1:**
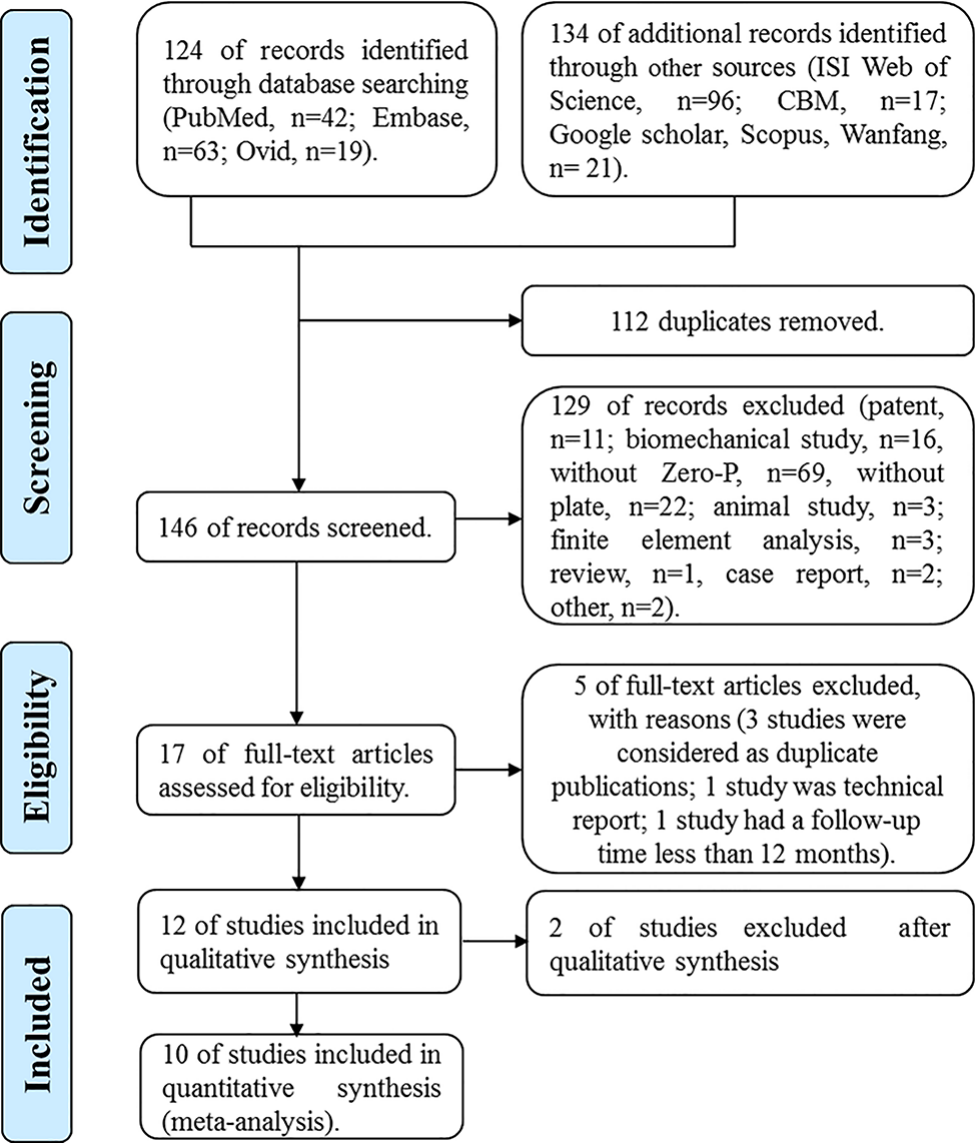
Flow diagram of literature search.

A total of 258 records were retrieved according to the search strategy. Among these, 124 records were identified through database searching (PubMed, n = 42; Ovid, n = 19; Embase, n = 63), and 134 additional records were identified via other sources (ISI Web of Science, n = 96; CBM, n = 17; Google Scholar, Scopus, Wanfang, n = 21). After review by title, abstract or full text, 248 reports were excluded for not meeting the inclusion criteria. Five full-text excluded studies and reasons for their exclusion are shown in **S1 File**. Finally, 10 eligible records were included in this study[16–20, 24–28] (**Fig 1**). The included studies were published between 2012 and 2014 and included 719 cervical spondylosis patients (Zero-P group, 343; Plate group, 376). The basic characteristics are summarized in **Table 1.** The statistically similar baseline between Zero-P and anterior plate groups is summarized in **Table 2**. The preoperative and postoperative evaluation data are summarized in **Table 3**and **Table 4**, respectively. Among the included studies were two small-sample RCTs, three prospective studies and five retrospective studies.

**Table 1:** Basic character of included studies.

Study	Country	Language	F (M)	Simple Size (M\F)		Mean Age (Year)		Operation Time (Min)		Blood Loss (mL)	
				Zero-P	Plate	Zero-P	Plate	Zero-P	Plate	Zero-P	Plate
**18 ^**a**^**	America	English	13.9	35(16\19)	35(18\17)	56.8±1.6	51.5±2	107.1±4.5	109.4±5.7	53.8±4.3	103.3±22.3
**24 ^**c**^**	China	English	24	23(14\9)	23(10\13)	49.3±8.3	50.1±6.4	94.7±4.6	86.1±4.1	87.1±3.5	89.5±3.4
**25 ^**b**^**	China	English	19.6	39(23\16)	50(29\21)	50.3	52.6	\	\	\	\
**16 ^**c**^**	Japan	English	24	24(21\3)	22(21\1)	40.9±7.2	41.6±7.0	116.4±17.1	128.5±17.4	27.7±19.0	30.1±25.8
**19 ^**a**^**	China	English	19	83(47\36)	107(58\49)	43.6	44.9	130.3±34.4	153.5±43.2	170.0±46.0	185.0±52.0
**26 ^**a**^**	Korea	English	13	20(7\13)	20(13\7)	50.0±12.0	44.3±9.7	\	\	\	\
**27 ^**b**^**	Czech	English	24	44(26\18)	33(19\14)	50.2± 10.3	51.8±12.9	\	\	\	\
**20 ^**a**^**	China	English	24	30(18\12)	33(14\19)	56.8±11.0	54.0±10.0	103.8±27.5	124.4±28.3	68.5±23.4	95.2±33.1
**28 ^**a**^**	China	English	33	22(11\11)	25(10\15)	50.9±8.8	53.7±8.9	98.2±15.6	105.4±14.4	87.9±12.0	92.4±11.3
**17 ^**b**^**	China	English	14.6	23(17\7)	28(21\7)	55.3±8.9	56.4±7.9	116.9±17.7	128.2±18.9	102.6±27.0	108.9±26.5

Note: ^a^ Retrospective, ^b^ Prospective, ^c^ Randomized controlled trial; F.: Follow-up time; \: no data available.

**Table 2:** Comparison of baseline of Zero-P and anterior plate in cervical fusion.

Characteristic	Study									
	18	24	25	16	19	26	27	20	28	17
Mean age	*	NS	NC	NS	NS	NS	NS	NS	NS	NS
Gender	NS	NS	NC	NS	NS	NS	NS	NS	NS	NS
Length of follow-up	NS	NS	NC	NS	\	NS	NS	NS	NS	NS
Locations of fusions	NS	NS	NC	\	NC	NC	NS	\	\	\
Number of levels operated	\	NS	NC	NS	NS	NC	NC	NS	NS	NS
Preoperative JOA Score	NS	NC	NS	\	\	\	\	NS	NS	NS
Preoperative NDI	\	\	\	\	NC	\		NS	\	NS
Preoperative VAS	\	NC	NS	NS	NC	\	\	\	\	\
Preoperative Segmental Angle	\	\	\	NS	\	NC	NC	\	\	\
Preoperative Cervical Angle	\	\	NS	NS	NC	NC	NC	NS	\	NS

*: Statistical differences (p<0.05)

NS: Not Significant (p>0.05)

JOA: Japanese Orthopedic Association

NDI: Neck Disability Index

VAS: Visual Analogue Scale

NC: not compared; the author published the data with Mean value but did not provide the SD. We considered that the difference of the two procedures could not be compared

\: no data available.

**Table 3:** Preoperative evaluation data.

Study	JOA SCORE		NDI		VAS		S Cobb's Angle (°)		C Cobb's Angle (°)	
	Zero- P	Plate	Zero- P	Plate	Zero- P	Plate	Zero- P	Plate	Zero-P	Plate
**18**	13.6±0.3	13.7±0.2	\	\	\	\	\	\	\	\
**24**	7.03	7.16	\	\	7.8	7.43	\	\	\	\
**25**	9.7±1.7	10.0±1.4	\	\	7.3±1.3	6.4±1.2	\	\	8.9±10.1	1.5±11.1
**16**	\	\	\	\	4.3±1.4	4.5±1.3	1.9±2.3	1.8 ± 2.6	6.2±3.3	5.6 ± 3.9
**19**	\	\	50.6±0.7	51.6±0.8	78.6±0.31	78.0±1.8	\	\	10.8±2.9	10.4±2.9
**26**	\	\	\	\	\	\	1.1±5.3	-2.2±7.6	7.7±12.7	9.9±12.3
**27**	\	\	25.4±4.0	23.7±4.7	\	\	2.5±1.5	4±1.7	7.1±3.8	11.6±4.4
**20**	9.1±2.4	9.4±2.0	36.8±16.0	37.6±16.7	\	\	\	\	12.4±9.1	11.9±10.0
**28**	9.1±1.4	9.2±1.6	\	\	\	\	\	\	\	\
**17**	8.6±1.8	9.0±1.5	13.2±3.5	12.5±3.2	\	\	\	\	10.2±5.4	10.8±5.2

Note: JOA, Japanese orthopedic association; NDI, Neck disability index; VAS, Visual analog scale; S Cobb's Angle, segmental Cobb’s angle; C Cobb's Angle, Cervical Cobb’s angle; \: no data available.

**Table 4:** Postoperative evaluation data.

Study	JOA		NDI		VAS		S Cobb's Angle		C Cobb's Angle		Early D		Last D		Fusion	
	ZP	AP	ZP	AP	ZP	AP	ZP	AP	ZP	AP	ZP	AP	ZP	AP	ZP	AP
**18**	15.54±0.47	15.31±0.29	\	\	\	\	\	\	\	\	11	14	1	7	\	\
**24**	10.4	10.3	\	\	0.91	1.26	\	\	\	\	\	\	0	4	25	24
**25**	15.13±1.232	15.02±1.56	\	\	1.13±0.64	0.6±0.52	\	\	17.58±8.69	16.75±8.29	\	\	0	1	\	\
**16**	\	\	\	\	0.9±0.8	1.1±0.7	6.0±3.0	6.9±3.1	15.03±0.50	14.10±4.30	\	\	0	4	\	\
**19**	\	\	24.9±1.0	24.7±1.3	22±2.8	24.7±1.1	\	\	16.30±3.20	18.0±3.60	9	10	0	0	22	21
**26**	\	\	\	\	\	\	3.46±5.21	3.85±4.87	12.51±11.07	14.73±9.22	\	\	0	5	\	\
**27**	\	\	12.7±2.7	15.8±3.1	\	\	4.1±1.0	5.1±1.1	7.2±3.8	14.9±4.3	1	6	0	0	\	\
**20**	13.86±1.7	14.12±1.13	\	\	\	\	\	\	\	\	6	14	0	9	\	\
**28**	14.9±2.1	14.7±2.0	11.8±9.0	12.7±9.1	\	\	\	\	16.10±7.90	16.60±9.30	10	10	1	3	41	29
**17**	13.96±1.52	13.57±1.35	3.56±1.77	3.93±1.66	\	\	\	\	16.93±2.78	18.36±3.67	2	8	0	1	\	\

Note: JOA, Japanese orthopedic association; NDI, Neck disability index; VAS, Visual analog scale; S Cobb's Angle, segmental Cobb’s angle; C Cobb's Angle, Cervical Cobb’s angle; Early D, Dysphagia within six weeks; Last D, Dysphagia at last follow-up; ZP, Zero-P; AP, anterior plate; \: no data available.

### Assessment of study quality

Two studies were excluded for unclear inclusion and exclusion criteria, unspecified locations of fusions, an unspecified number of vertebrae operated on, and other methodological ambiguities. The quality assessment of two RCTs[16, 24] is shown in **S5 Table**. Both RCTs clearly reported specific definitions of diagnosis, selection criteria and good representativeness of the source population in the control group. Each study obtained nine scores, suggesting high quality. The assessment of the non-randomized studies is shown in **S6 Table.** The eight cohort studies reported a clearly stated aim, follow-up period appropriate to the aim of the study, loss to follow-up less than 5%, adequate control group, contemporary groups and adequate statistical analyses. However, no study adequately reported an unbiased assessment of the study endpoint or prospective calculation of the study size. Only three studies[17, 19, 25] reported prospective collection of data. All, except the study by Hofstetter et al.[18], clearly described the inclusion of consecutive patients and endpoints appropriate to the aim of the study. The baseline equivalence of groups was well established except that in Miao et al.[25].

### Operation time

**Fig 2:**
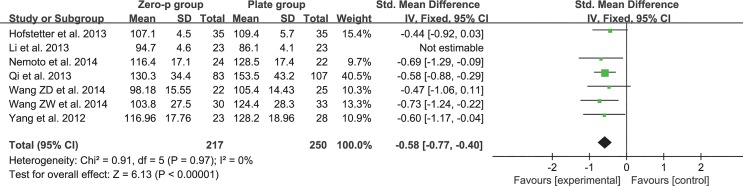
Standardized mean difference (SMD) estimate for operation time.

Data regarding operation time were available in seven separate studies with a total of 513 patients (Zero-P group, 240; Plate group, 273)[16–20, 24, 28]. The fixed-effects model was applied to determine the operation time between the two groups after sensitivity analysis of eliminating the study of Li et al.[24] (Heterogeneity: I^2^ = 0%). The pooled SMD was -0.58 (95% CI = -0.77 to 0.40, p < 0.01) for patients in the Zero-P group compared to the plate group, suggesting that there was a significant difference between these two treatment groups in terms of operation time (**Fig 2**).

### Blood loss

**Fig 3:**
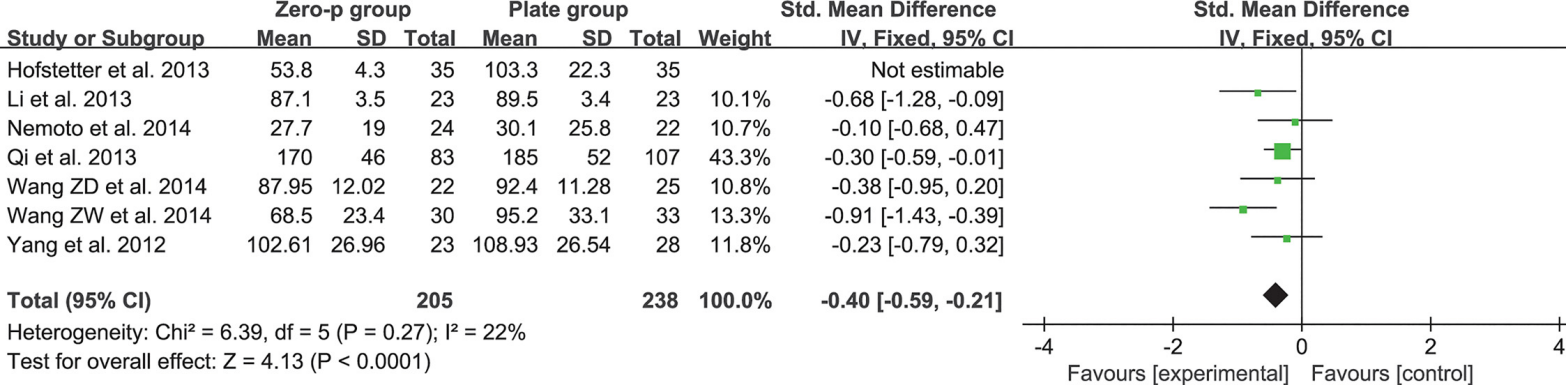
Standardized mean difference (SMD) estimate for blood loss.

The relevant data regarding the blood loss were documented in seven articles including 513 patients (Zero-P group, 240; Plate group, 273)[16–20, 24, 28]. The fixed-effects model was applied to determine the difference in blood loss between the two groups after sensitivity analysis of eliminating the study of Hofstetter et al.[18] (Heterogeneity: I^2^ = 22%). The remaining six trials showed that the Zero-P group had a significantly reduced intraoperative blood loss compared to the Plate group. Pooling of relevant data also showed a significant difference between the two groups (SMD = -0.40, 95% CI (-0.59 to -0.21), p < 0.01) (**Fig 3**).

### JOA score

**Fig 4:**
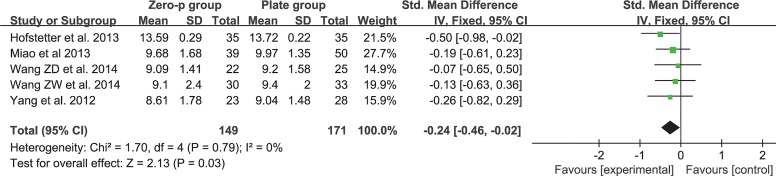
Standardized mean difference (SMD) estimate for preoperative JOA score.

**Fig 5:**
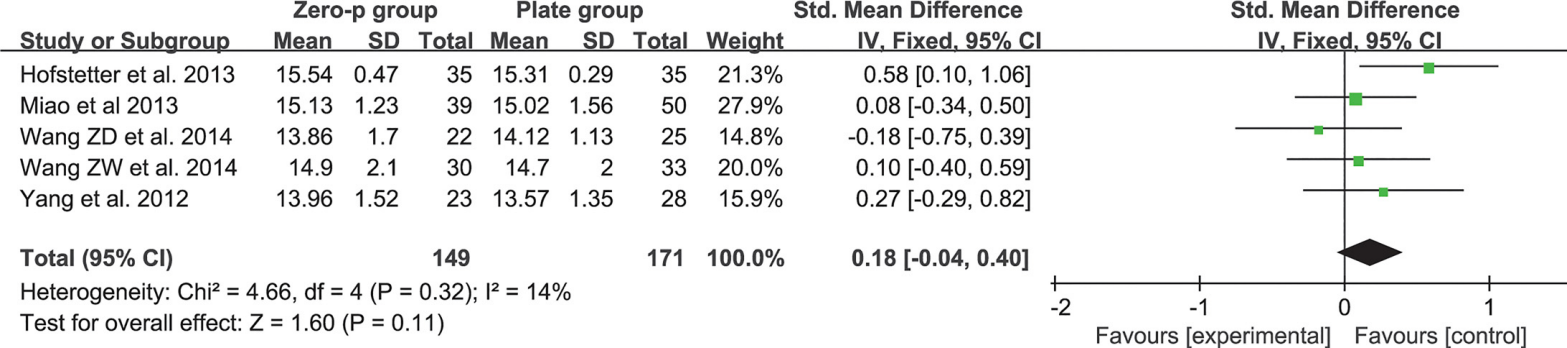
Standardized mean difference (SMD) estimate for postoperative JOA score.

Five studies consisting of 320 patients reported the preoperative and postoperative JOA scores (Zero-P group, 149; Plate group, 171)[17, 18, 20, 25, 28]. The fixed-effects model was applied to compare the JOA score between the two groups. The pooled estimate revealed that the preoperative JOA score of the Zero-P group was lower and that the difference was significant between the two groups without heterogeneity (SMD = -0.24, 95% CI (−0.46 to −0.02), p = 0.03) (**Fig 4)**. The pooled estimate of the postoperative JOA score revealed no significant difference (SMD = 0.18, 95% CI (−0.04 to 0.40), p = 0.11), with low heterogeneity: I^2^ = 14% **(Fig 5**).

### NDI

**Fig 6:**
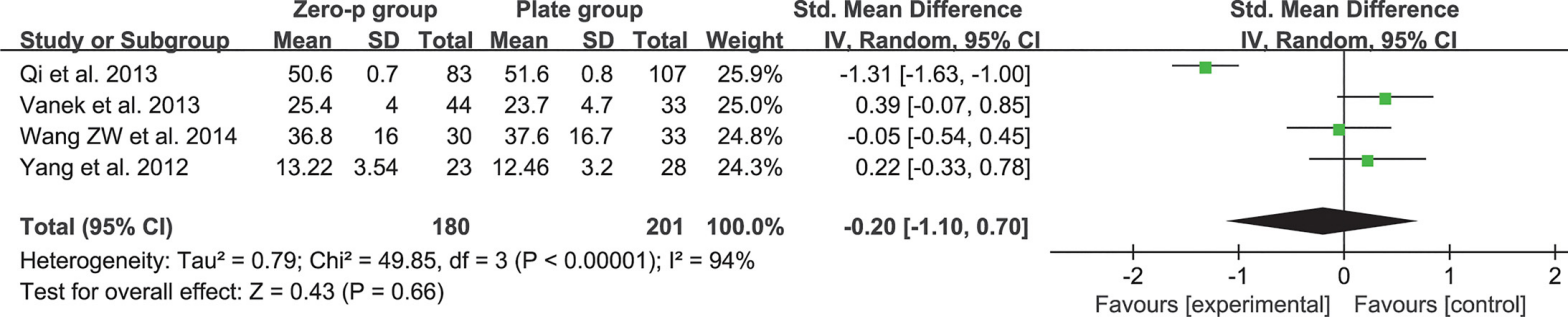
Standardized mean difference (SMD) estimate for preoperative NDI.

**Fig 7:**
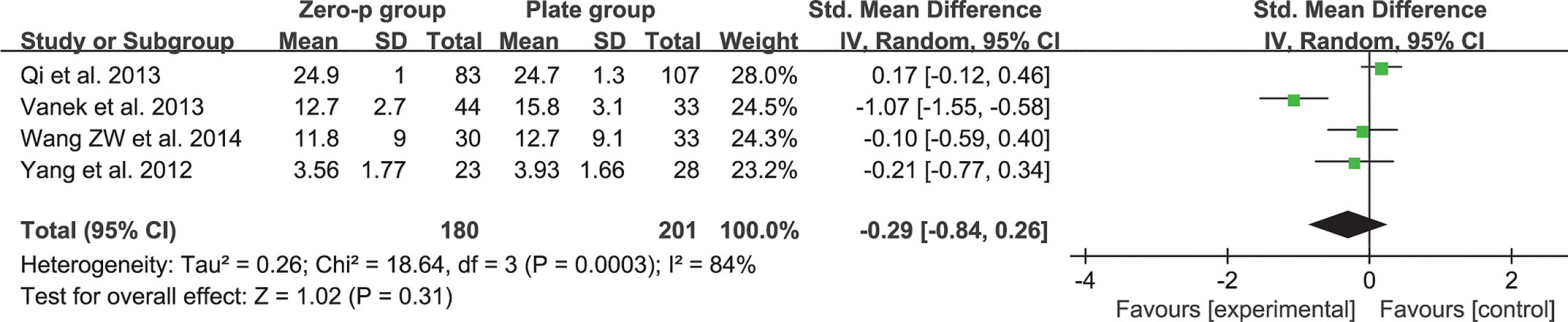
Standardized mean difference (SMD) estimate for postoperative NDI.

Four studies consisting of 381 patients reported the preoperative and postoperative neck NDI (Zero-P group, 180; Plate group, 201)[17, 19, 20, 27]. The fixed-effects model was applied to compare the preoperative or postoperative NDI between the two groups but was accompanied by high heterogeneity (preoperative, I^2^ = 94%; postoperative, I^2^ = 84%). The cause of heterogeneity was investigated by subgroup analysis and sensitivity analysis but could not be ascertained. Finally, a random-effects model was applied. Pooled data of preoperative NDI from the relevant studies revealed no significant difference between the two groups (SMD = −0.20, 95% CI (−1.1 to 0.7), p = 0.66 > 0.05) and high heterogeneity: I^2^ = 94% **(Fig 6)**. Pooled data of postoperative NDI did not reveal any significant difference (SMD = −0.29, 95% CI (−0.84 to 0.26), p = 0.31 > 0.05) and high heterogeneity: I^2^ = 84% (**Fig 7**).

### VAS

**Fig 8:**
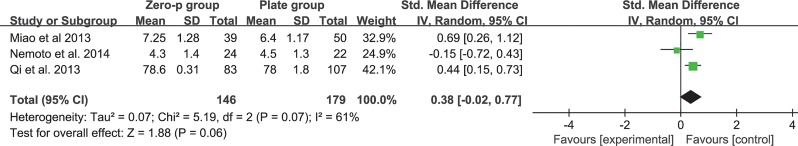
Standardized mean difference (SMD) estimate for preoperative VAS.

**Fig 9:**
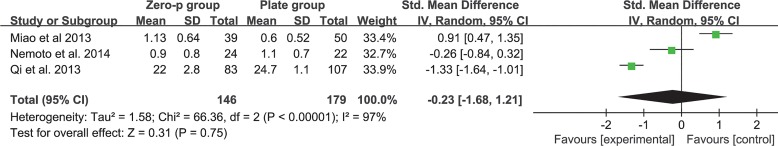
Standardized mean difference (SMD) estimate for postoperative VAS.

Three studies consisting of 325 patients reported preoperative and postoperative neck VAS scores (Zero-P group, 146; Plate group, 179)[16, 19, 25]. The fixed-effects model was applied to compare the preoperative and postoperative VAS between the two groups. High heterogeneity was detected (preoperative, I^2^ = 61%; postoperative, I^2^ = 97%). The cause of heterogeneity could not be determined by subgroup analysis or sensitivity analysis. Finally, a random-effects model was applied. Pooled data of preoperative VAS from the three relevant studies revealed no significant difference (SMD = 0.38, 95% CI (−0.20 to 0.77), p = 0.06 > 0.05) and high heterogeneity: I^2^ = 61% (**Fig 8**). Pooled data of postoperative VAS revealed no significant difference (SMD = 0.38, 95% CI (−0.02 to −0.77), p = 0.75 > 0.05) and significant heterogeneity: I^2^ = 97% (**Fig 9**).

### Postoperative segmental Cobb’s angle

**Fig 10:**
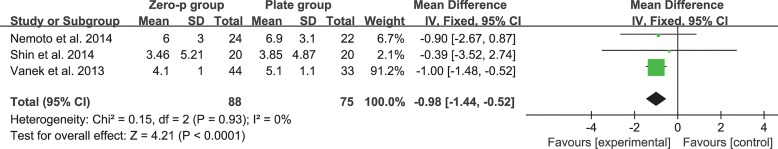
Mean difference (MD) estimate for postoperative segmental Cobb’s angle.

Three studies reported a postoperative segmental Cobb’s angle in 163 patients (Zero-P group, 88; Plate group, 75)[16, 26, 27]. The fixed-effects model was applied to compare the postoperative segmental Cobb’s angle between the two groups. Pooled data from the three relevant studies revealed a significant difference (MD = −0.98, 95% CI (−1.44 to −0.52), p < 0.01) without heterogeneity: I^2^ = 0% (**Fig 10**).

### Postoperative cervical Cobb’s angle

**Fig 11:**
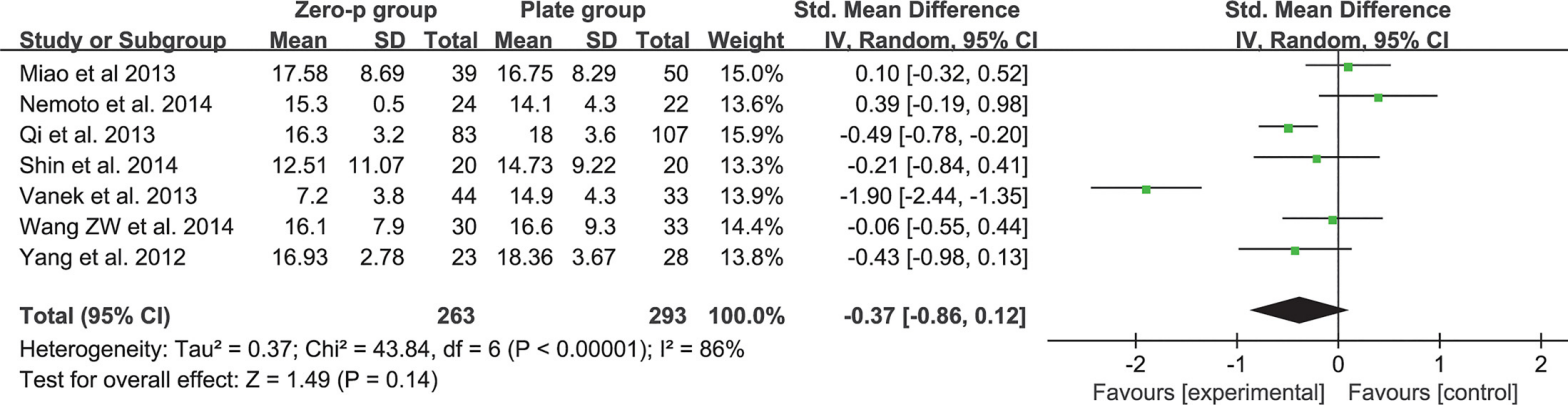
Standardized mean difference (SMD) estimate for postoperative cervical Cobb’s angle.

Seven studies consisting of 556 patients reported postoperative cervical Cobb’s angle (Zero-P group, 263; Plate group, 293)[16, 17, 19, 20, 25–27]. The heterogeneity in comparing the postoperative cervical Cobb’s angle between the two groups was significant in the fixed-effects model (I^2^ = 86%). Subgroup analysis according to race or sensitivity analysis did not reveal the cause. Pooled data of the random-effects model from the relevant studies revealed no significant difference (SMD = −0.37, 95% CI (−0.86 to 0.120), p = 0.14 > 0.05) and significant heterogeneity: I^2^ = 86% (**Fig 11**).

### Fusion rate

**Fig 12:**
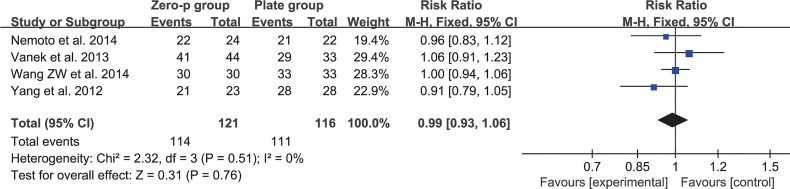
Risk ratio (RR) estimate for fusion rate.

Four studies consisting of 237 patients reported the fusion rate at the end of follow-up (Zero-P group, 121; Plate group, 116)[16, 17, 20, 27]. The fixed-effects model was applied to compare dysphagia rate at the end of follow-up between the two groups. This comparison did not show any significant difference between the two groups (RR = 0.99, 95% CI (0.93 to 1.06), p = 0.76 > 0.05) and no heterogeneity: I^2^ = 0% (**Fig 12**).

### Dysphagia within six weeks

**Fig 13:**
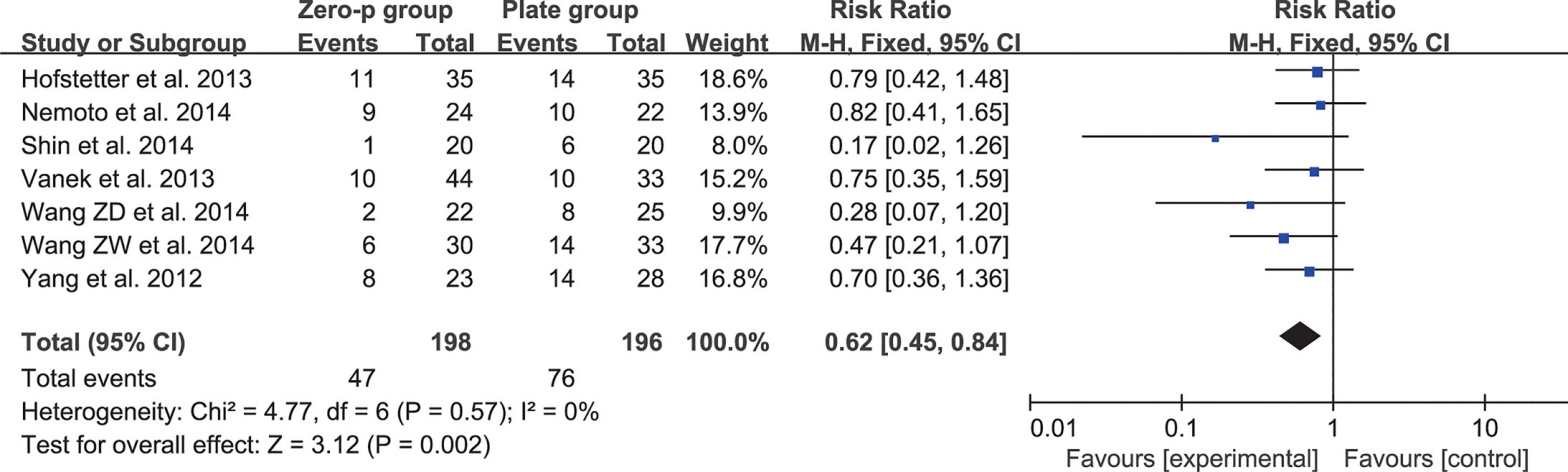
Risk ratio (RR) estimate for postoperative dysphagia at early stage.

**Fig 14:**
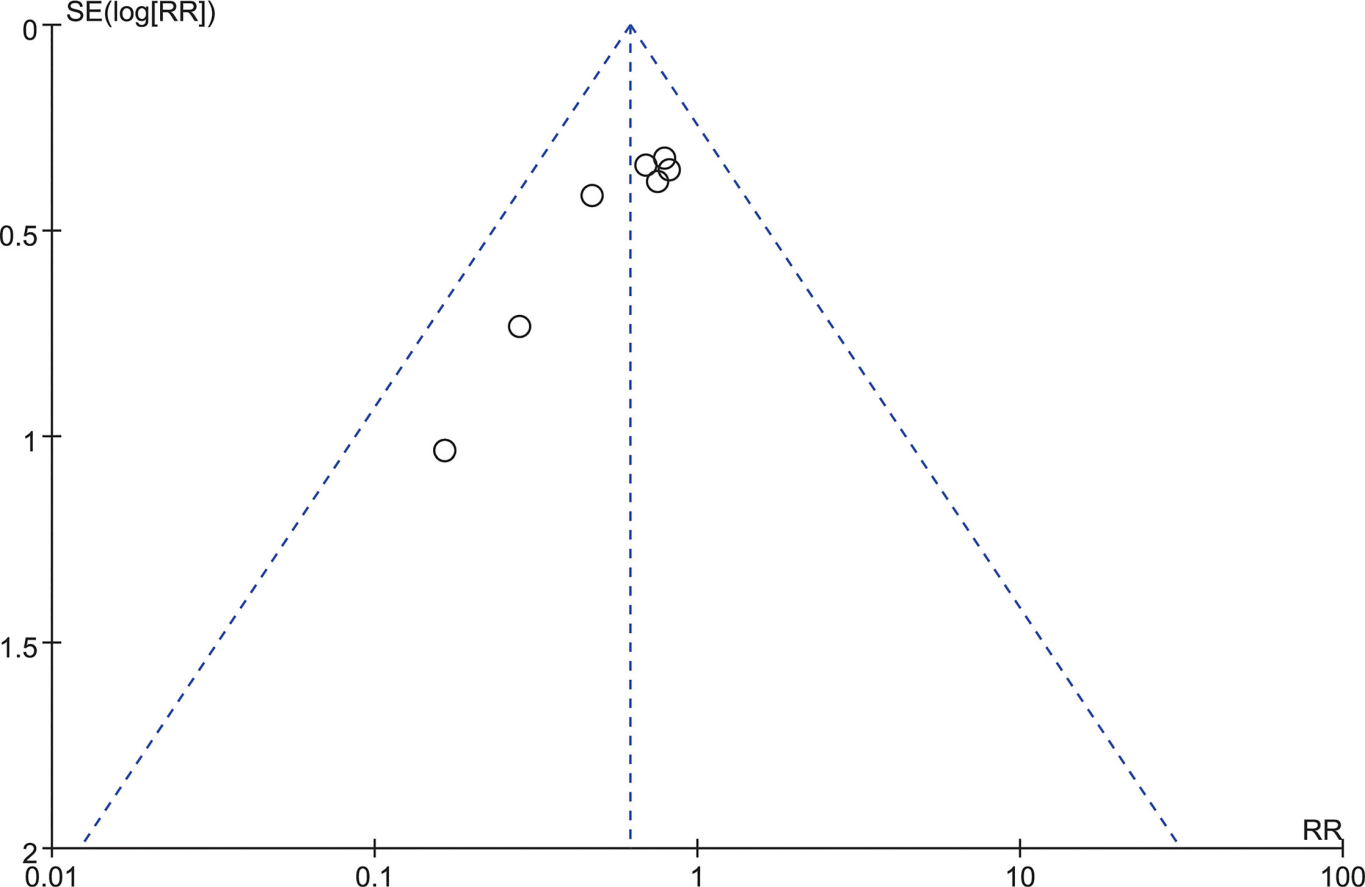
Funnel plot for publication bias of dysphagia within six weeks.

Seven studies consisting of 394 patients reported dysphagia rate within six weeks (Zero-P group, 198; Plate group, 196)[16–18, 20, 26–28]. The fixed-effects model was applied to compare the dysphagia rate within six weeks between the two groups. This comparison showed a significant difference between the two groups (RR = 0.62, 95% CI (0.45 to 0.84), p < 0.01) with no heterogeneity: I^2^ = 0% (**Fig 13**). The funnel plot for publication bias is shown in **Fig 14**.

### Dysphagia at the end of follow-up

**Fig 15:**
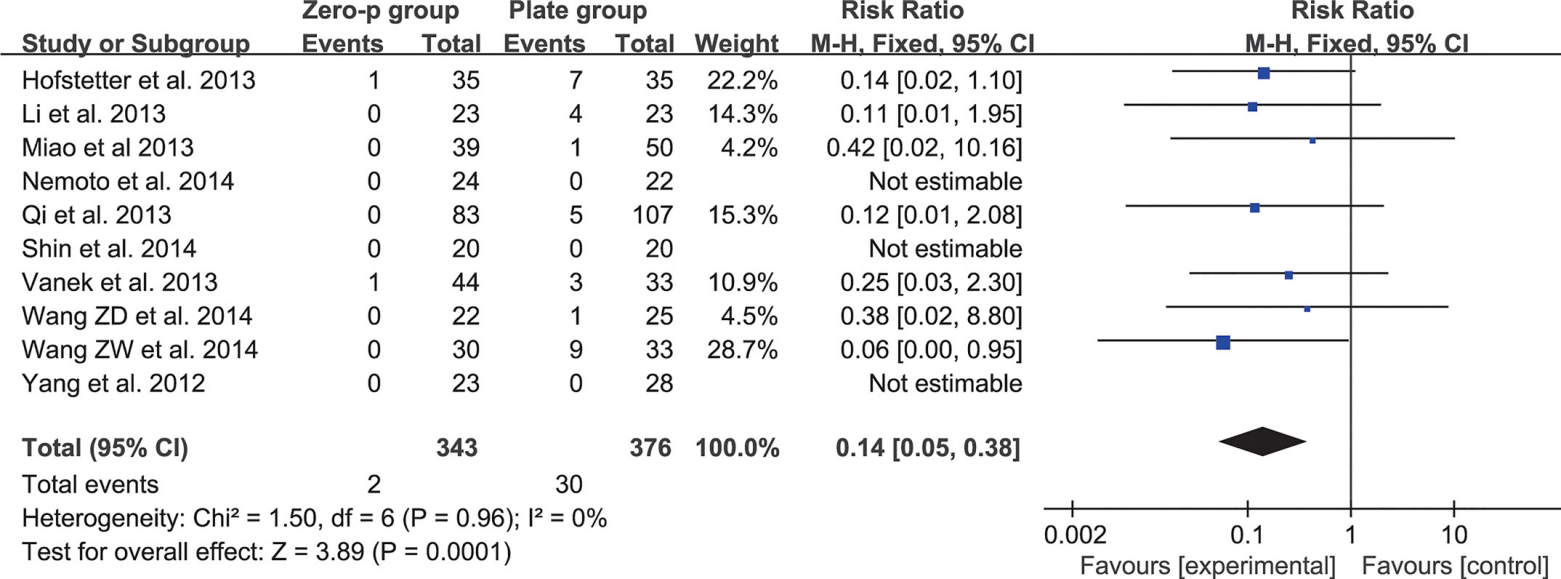
Risk ratio (RR) estimate for postoperative dysphagia at last follow-up.

**Fig 16:**
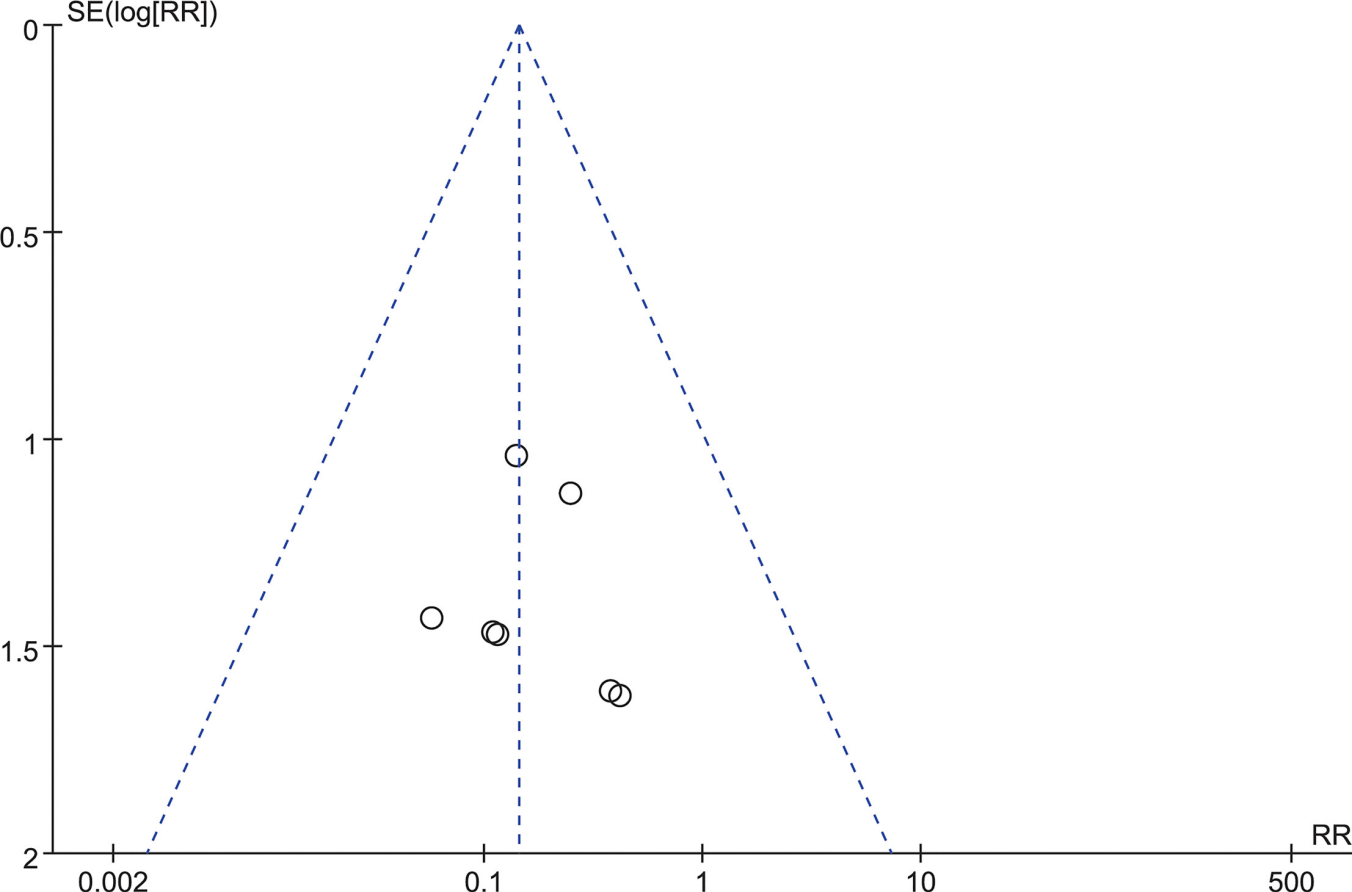
Funnel plot for publication bias of dysphagia at last follow-up.

Ten studies consisting of 719 patients reported the dysphagia rate at the end of follow-up (Zero-P group, 343; Plate group, 376)[16–20, 24–28]. Three studies were excluded because there were no events data[16, 17, 26]. The fixed-effects model was applied to compare the dysphagia rate at the end of follow-up between the two groups. This comparison showed a significant difference between the two groups (RR = 0.15, 95% CI (0.05 to 0.39), p < 0.01) and no heterogeneity: I^2^ = 0% (**Fig 15**). The funnel plot for publication bias is shown in **Fig 16**.

## Discussion

ACDF has been widely used in the treatment of cervical spondylosis[2]. During this procedure, an anterior cervical plate is usually applied to enhance the cervical stability, increase the interbody fusion rate and prevent graft dislocation or subsidence[3, 29]. However, several postoperative complications are closely associated with the anterior plate, as the plate can lead to the compression or stimulation of vital structures[27]. Zero-P is a stand-alone anchored spacer designed to reduce the profile of the anterior plate in cervical fusion and to simultaneously provide the spine a stable biomechanical environment[12, 13]. Several relevant clinical studies have compared Zero-P and anterior plate techniques following ACDF[16–20, 24–28]; however, the evidence regarding whether Zero-P is superior to anterior plate is insufficient. Therefore, we conducted a meta-analysis to evaluate which device is the optimal implant for cervical fusion.

This meta-analysis, consisting of 2 RCTs, 3 prospective and 5 retrospective studies including 719 patients, compared the outcomes of Zero-P and anterior plate fusion and showed that Zero-P was safer and more effective, with significantly reduced operation time and blood loss, a significant improvement of postoperative JOA score, and a similar reduction of postoperative NDI and VAS score as compared with anterior plate. Zero-P also had a lower incidence of postoperative dysphagia at early stage as well as at last follow-up. These two procedures had a similar fusion rate. However, the anterior plate group had a greater postoperative segmental and cervical Cobb’s angle than the Zero-P group.

### Safety

The pooled data of postoperative outcomes suggested that Zero-P is safe for cervical spondylosis. Compared with the plate group, the operating time and blood loss in the Zero-P group were lower (p < 0.01). It is noteworthy that the heterogeneity was extremely high if the study of Li et al. was included in the analysis of operating time. High heterogeneity was also observed in the analysis of blood loss. However, no remarkable differences could be detected in the detailed assessment of these two studies. The broad inclusion criteria may explain this phenomenon. Considering that the operative levels in these two studies were not clearly described, multilevel fusion may be included. In this condition, a longer operating time is required, theoretically leading to a higher volume of blood loss than single-level fusion. Therefore, the reliability of these results was investigated following sensitivity analysis. No heterogeneity existed after excluding the study of Li et al. (**Fig 2**). The heterogeneity in the blood loss comparison was low (I^2^ = 22%) (**Fig 3**).

### Efficacy

The efficacy of the cervical-spine operation was often evaluated by objective or subjective scales, such as JOA score, NDI and VAS. A higher postoperative JOA score and lower NDI or VAS suggested better clinical outcome. Our study revealed that there was a significant difference in the preoperative JOA scores (**Fig 4**). However, there was no significant difference in the postoperative JOA scores (**Fig 5**). This finding indicated that the improvement in the JOA score was significantly higher in the Zero-P group than in the anterior plate group. However, a significant difference was not observed either in preoperative and postoperative NDI (**Fig 6 and Fig 7**) or in preoperative and postoperative VAS (**Fig 8 and Fig 9**), indicating that the decrease in NDI and VAS in the Zero-P group was similar to that in the anterior plate group. Although the heterogeneities were extremely high in the comparison of NDI and VAS between groups, the subgroup and sensitivity analyses did not find the cause, which may be attributed to the subjectivity of the two scales. Overall, the Zero-P group exhibited a higher JOA score improvement and similar NDI and VAS reduction in comparison with the anterior plate group. However, due to the high heterogeneity in the pooled data of NDI or VAS, additional studies are needed.

### Radiological outcome

The anterior plate group had significantly greater postoperative segmental and cervical Cobb’s angle than the Zero-P group at last follow-up. These results may be related to the following factor: the Zero-P technique fuses two vertebral bodies using an interbody device, in which case the segmental sagittal alignment is not changed substantially, whereas the anterior plate fusion remodels the stable cervical spine with a plate that is longer than the interbody space, which can greatly affect the sagittal alignment. However, the cause of the heterogeneity of the postoperative cervical Cobb’s angle could not be determined. The finding that the anterior plate technique produced a greater postoperative cervical Cobb’s angle should be accepted with caution. Both the Zero-P device and the anterior plate can provide a stable biomechanical environment that dramatically promotes vertebral body fusion[12, 13]. This similarity may explain why the fusion rates in two groups were similar.

### Complications

Complications are a very important factor that is closely associated with the outcome of an operation. The most concerning postoperative complication after anterior plate fusion was postoperative dysphagia[6, 10, 15]. The etiology of this symptom may be multifactorial, including the contact of the plate with the esophagus[6]. In addition, the risk of postoperative dysphagia increases with the number of fused vertebrae[27]. Our study suggested that the Zero-P device could significantly reduce the rate of dysphagia within six weeks and at last follow-up. These results were surprising, as Zero-P requires a very small area, making it potentially more challenging for spinal surgeons. Considering that this low-profile device provided the same biomechanics for the cervical spine as the anterior plate, its lower incidence of earlier and later dysphagia is an advantage. However, it is worth noting that the two RCTs reported no significant difference in the later-stage dysphagia rate in the Zero-P group and anterior plate group. This lack of a detectable difference may have arisen because of the small sample size of the two studies.

### Limitations

The limitations of this study must be acknowledged. First, among the ten enrolled studies, there were only two RCTs, and the sample size of the Zero-P group and the control group was small. Including only ten studies limited the reliability of our meta-regression; thus, the potential risk factors could not be effectively investigated by meta-regression. Second, different segmental fusion techniques were performed, which could affect the outcome of operation time and blood loss. Multiple-segmental fusion requires a greater operation time and results in more blood loss. Our study revealed low heterogeneities in the primary end points and most of the secondary end points, and the sensitivity analysis using the random-effects model suggested no significant differences (**S7 Table**), however, clinical and methodological heterogeneity could not be completely eliminated due to the inclusion of cohort studies. Third, the broad inclusion criteria and different levels of surgical technologies may contribute to clinical heterogeneity. Fourth, the limitation of publication bias should be recognized in this study. Generally, a test for funnel plot asymmetry is recommended when there are at least 10 studies included in a meta-analysis. In our study, fewer than ten studies were included in the analysis of primary or secondary end points; thus, the power of the tests was too low to distinguish chance from real symmetry or asymmetry.

## Conclusion

This meta-analysis compared two fusion procedures, Zero-P and anterior plate, in anterior cervical fusion. Our results suggest that Zero-P is a safe and effective procedure with a similar fusion rate and lower incidence of postoperative dysphagia than the use of an anterior plate. However, the results of this meta-analysis should be accepted with caution because of the limitations of the study. Further evaluation and large-sample RCTs are required to confirm and update the results of this study.

## Supporting Information

S1 Fig The PubMed search form. (JPG)

S2 Fig The Embase search form. (JPG)

S3 Fig The Ovid search form. (JPG)

S4 Fig The Web of Science search form. (JPG)

S5 Fig The China Biology Medicine search form. (JPG)

S1 Table PRISMA check list. (DOC)

S2 Table Data-combining formula. (DOCX)

S3 Table Method for assessing the quality of a randomized control trail by Chalmers et al. (DOCX)

S4 Table The revised and validated version of MINORS. (DOCX)

S5 Table Quality assessment of RCT. (DOCX)

S6 Table Quality assessment of non-randomized studies. (DOCX)

S7 Table Comparison of two types of models. (DOCX)

S1 File Five full-text excluded studies and reasons for their exclusion. (ZIP)

## References

[1] Ghogawala Z, Benzel EC, Heary RF, Riew KD, Albert TJ, Butler WE, et al (2014) Cervical spondylotic myelopathy surgical trial: randomized, controlled trial design and rationale. Neurosurgery. 75(4):334–46. 10.1227/neu.0000000000000479 . 24991714 PMC4633023

[2] Smith GW, Robinson RA. (1958) The treatment of certain cervical-spine disorders by anterior removal of the intervertebral disc and interbody fusion. J Bone Joint Surg Am. 40-a(3):607–24. . 13539086

[3] Karikari IO, Jain D, Owens TR, Gottfried O, Hodges TR, Nimjee SM, et al (2014) Impact of subsidence on clinical outcomes and radiographic fusion rates in anterior cervical discectomy and fusion: a systematic review. J Spinal Disord Tech. 27(1):1–10. 10.1097/BSD.0b013e31825bd26d . 24441059

[4] Park Y, Maeda T, Cho W, Riew KD. (2010) Comparison of anterior cervical fusion after two-level discectomy or single-level corpectomy: sagittal alignment, cervical lordosis, graft collapse, and adjacent-level ossification. Spine J. 10(3):193–9. 10.1016/j.spinee.2009.09.006 . 19850532

[5] Buerba RA, Giles E, Webb ML, Fu MC, Gvozdyev B, Grauer JN. (2014) Increased Risk of Complications After ACDF in the Elderly: An Analysis of 6,253 Patients in the ACS-NSQIP Database. Spine (Phila Pa 1976). 39(25):2062–9. 10.1097/brs.0000000000000606 . 25271519

[6] Chen CJ, Saulle D, Fu KM, Smith JS, Shaffrey CI. (2013) Dysphagia following combined anterior-posterior cervical spine surgeries. J Neurosurg Spine. 19(3):279–87. 10.3171/2013.6.spine121134 . 23848353

[7] Rihn JA, Kane J, Albert TJ, Vaccaro AR, Hilibrand AS. (2011) What is the incidence and severity of dysphagia after anterior cervical surgery? Clin Orthop Relat Res. 469(3):658–65. 10.1007/s11999-010-1731-8 . 21140251 PMC3032867

[8] Anderson KK, Arnold PM. (2013) Oropharyngeal Dysphagia after anterior cervical spine surgery: a review. Global Spine J. 3(4):273–86. 10.1055/s-0033-1354253 . 24436882 PMC3854602

[9] Bazaz R, Lee MJ, Yoo JU. (2002) Incidence of dysphagia after anterior cervical spine surgery: a prospective study. Spine (Phila Pa 1976). 27(22):2453–8. 10.1097/01.brs.0000031407.52778.4b . 12435974

[10] Riley LH 3rd, Vaccaro AR, Dettori JR, Hashimoto R. (2010) Postoperative dysphagia in anterior cervical spine surgery. Spine (Phila Pa 1976). 35(9 Suppl):S76–85. 10.1097/BRS.0b013e3181d81a96 . 20407354

[11] Lee MJ, Bazaz R, Furey CG, Yoo J. (2005) Influence of anterior cervical plate design on Dysphagia: a 2-year prospective longitudinal follow-up study. J Spinal Disord Tech. 18(5):406–9. . 16189451 10.1097/01.bsd.0000177211.44960.71

[12] Scholz M, Reyes PM, Schleicher P, Sawa AG, Baek S, Kandziora F, et al (2009) A new stand-alone cervical anterior interbody fusion device: biomechanical comparison with established anterior cervical fixation devices. Spine (Phila Pa 1976). 34(2):156–60. 10.1097/BRS.0b013e31818ff9c4 . 19139665

[13] Schollhorn B, Burki A, Stahl C, Howard J, Forterre F. (2013) Comparison of the biomechanical properties of a ventral cervical intervertebral anchored fusion device with locking plate fixation applied to cadaveric canine cervical spines. Vet Surg. 42(7):825–31. 10.1111/j.1532-950X.2013.12044.x . 24033669

[14] Khaki F, Zusman NL, Nemecek AN, Ching AC, Hart RA, Yoo JU. (2013) Postoperative prevertebral soft tissue swelling does not affect the development of chronic dysphagia following anterior cervical spine surgery. Spine (Phila Pa 1976). 38(9):E528–32. 10.1097/BRS.0b013e31828a2992 . 23380821

[15] Kepler CK, Rihn JA, Bennett JD, Anderson DG, Vaccaro AR, Albert TJ, et al (2012) Dysphagia and soft-tissue swelling after anterior cervical surgery: a radiographic analysis. Spine J. 12(8):639–44. 10.1016/j.spinee.2012.03.024 . 22561176

[16] Nemoto O, Kitada A, Naitou S, Tachibana A, Ito Y, Fujikawa A. (2014) Stand-alone anchored cage versus cage with plating for single-level anterior cervical discectomy and fusion: a prospective, randomized, controlled study with a 2-year follow-up. Eur J Orthop Surg Traumatol. 10.1007/s00590-014-1547-4 . 25283362

[17] Yang L, Gu Y, Liang L, Gao R, Shi S, Shi J, et al (2012) Stand-alone anchored spacer versus anterior plate for multilevel anterior cervical diskectomy and fusion. Orthopedics. 35(10):e1503–10. 10.3928/01477447-20120919-20 . 23027488

[18] Hofstetter CC, Kesavabhotla K, Boockvar JA. (2013) Zero-profile Anchored Spacer Reduces Rate of Dysphagia Compared to ACDF With Anterior Plating. J Spinal Disord Tech. 10.1097/BSD.0b013e31828873ed . 23429316

[19] Qi M, Chen H, Liu Y, Zhang Y, Liang L, Yuan W. (2013) The use of a zero-profile device compared with an anterior plate and cage in the treatment of patients with symptomatic cervical spondylosis: A preliminary clinical investigation. Bone Joint J. 95 B(4):543–7. 10.1302/0301-620X.95B4.30992 . 23539708

[20] Wang Z, Jiang W, Li X, Wang H, Shi J, Chen J, et al (2015) The application of zero-profile anchored spacer in anterior cervical discectomy and fusion. Eur Spine J. 24(1):148–54. 10.1007/s00586-014-3628-9 . 25337859

[21] Moher D, Liberati A, Tetzlaff J, Altman DG. (2010) Preferred reporting items for systematic reviews and meta-analyses: The PRISMA statement. Int J Surg. 8(5):336–41. 10.1016/j.ijsu.2010.02.007 . 20171303

[22] Chalmers TC, Smith H Jr., Blackburn B, Silverman B, Schroeder B, Reitman D, et al (1981) A method for assessing the quality of a randomized control trial. Control Clin Trials. 2(1):31–49. . 7261638 10.1016/0197-2456(81)90056-8

[23] Slim K, Nini E, Forestier D, Kwiatkowski F, Panis Y, Chipponi J. (2003) Methodological index for non-randomized studies (minors): development and validation of a new instrument. ANZ J Surg. 73(9):712–6. . 12956787 10.1046/j.1445-2197.2003.02748.x

[24] Li Y, Hao D, He B, Wang X, Yan L. (2013) The Efficiency of Zero-Profile Implant in Anterior Cervical Discectomy Fusion: A Prospective Controlled Long-term Follow-up Study. J Spinal Disord Tech. 10.1097/bsd.0000000000000032 . 24136051

[25] Miao J, Shen Y, Kuang Y, Yang L, Wang X, Chen Y, et al (2013) Early follow-up outcomes of a new zero-profile implant used in anterior cervical discectomy and fusion. J Spinal Disord Tech. 26(5):E193–7. 10.1097/BSD.0b013e31827a2812 . 23168389

[26] Shin JS, Oh SH, Cho PG. (2014) Surgical Outcome of a Zero-profile Device Comparing with Stand-alone Cage and Anterior Cervical Plate with Iliac Bone Graft in the Anterior Cervical Discectomy and Fusion. Korean J Spine. 11(3):169–77. 10.14245/kjs.2014.11.3.169 . 25346764 PMC4206955

[27] Vanek P, Bradac O, Delacy P, Lacman J, Benes V. (2013) Anterior interbody fusion of the cervical spine with Zero-P spacer: prospective comparative study-clinical and radiological results at a minimum 2 years after surgery. Spine (Phila Pa 1976). 38(13):E792–7. 10.1097/BRS.0b013e3182913400 . 23524869

[28] Wang ZD, Zhu RF, Yang HL, Gan MF, Zhang SK, Shen MJ, et al (2014) The application of a zero-profile implant in anterior cervical discectomy and fusion. J Clin Neurosci. 21(3):462–6. 10.1016/j.jocn.2013.05.019 . 24262773

[29] Kaiser MG, Haid RW Jr, Subach BR, Barnes B, Rodts GE Jr. (2002) Anterior cervical plating enhances arthrodesis after discectomy and fusion with cortical allograft. Neurosurgery. 50(2):229–36; discussion 36–8. . 11844257 10.1097/00006123-200202000-00001

