# Supplementary figures and images for: Meta-Analysis Comparing Zero-Profile Spacer and Anterior Plate in Anterior Cervical Fusion
Source: PLoS One. 2015 Jun 11;10(6):e0130223. doi: 10.1371/journal.pone.0130223 (PMC4466022; doi:10.1371/journal.pone.0130223)

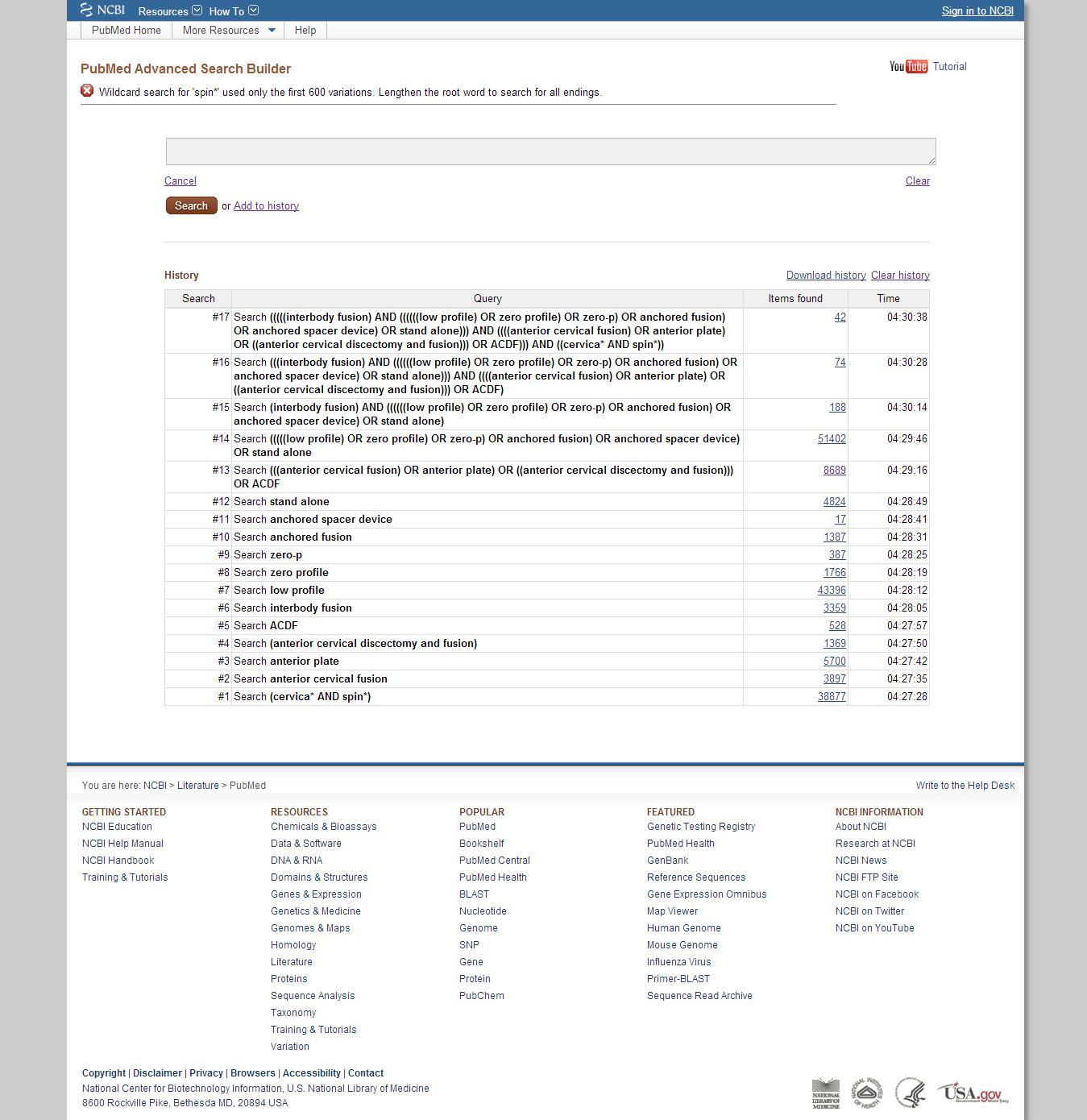

Supplement: S1 Fig — (JPG) [file pone.0130223.s001.jpg]

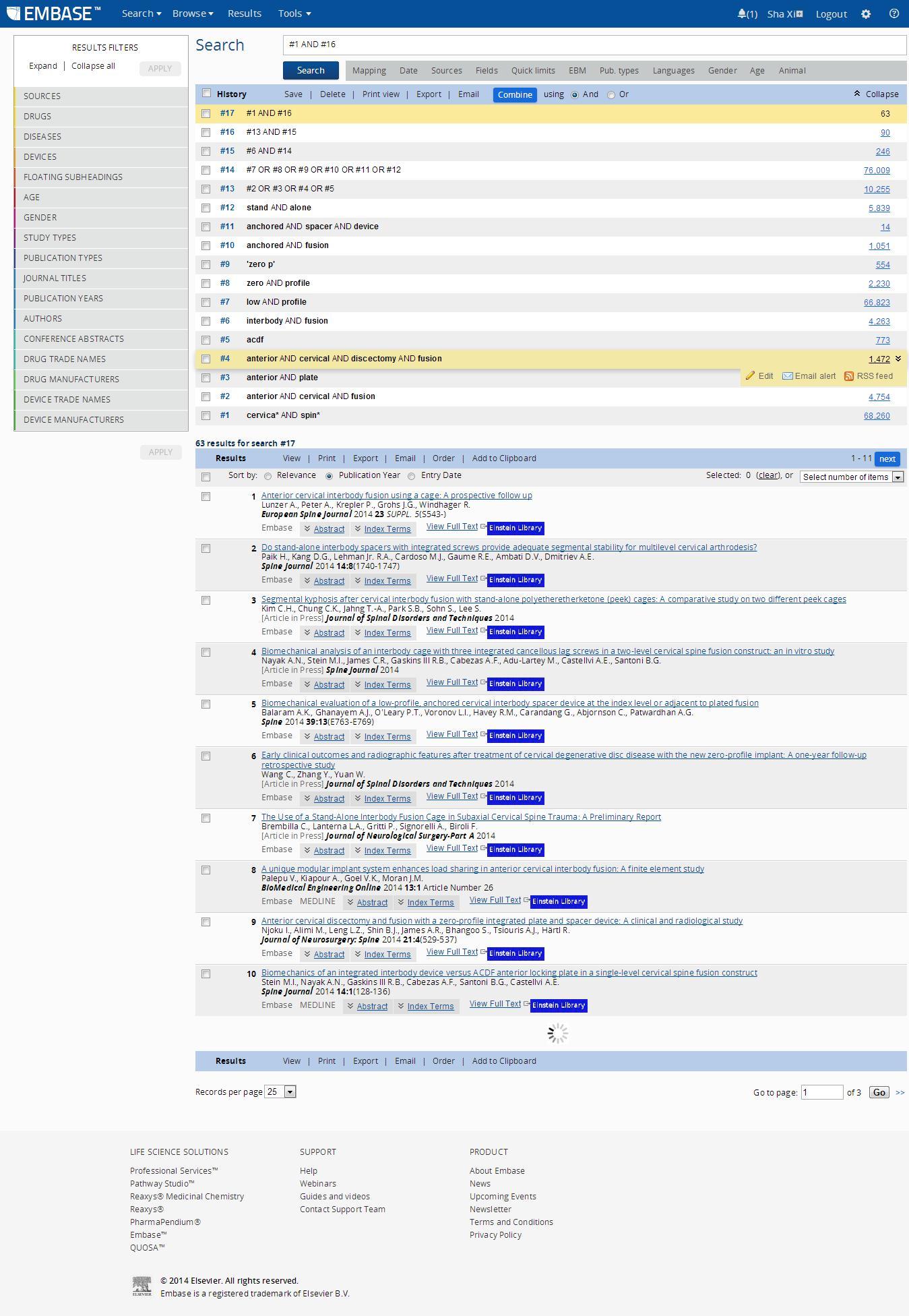

Supplement: S2 Fig — (JPG) [file pone.0130223.s002.jpg]

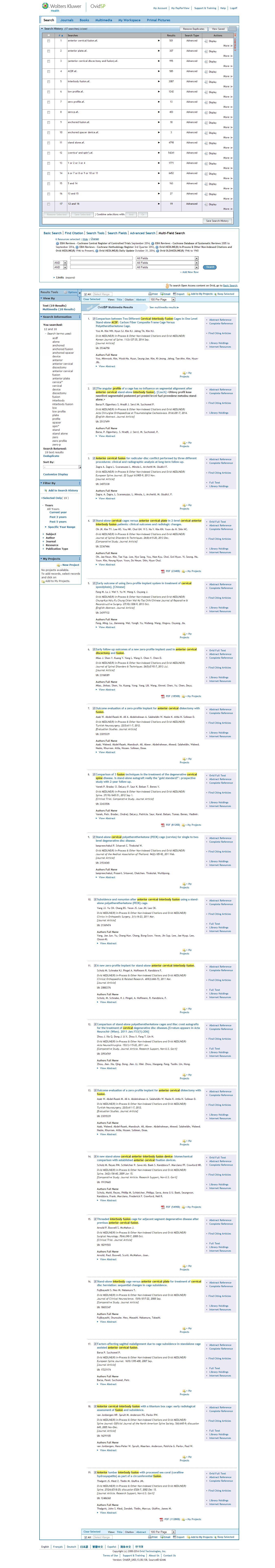

Supplement: S3 Fig — (JPG) [file pone.0130223.s003.jpg]

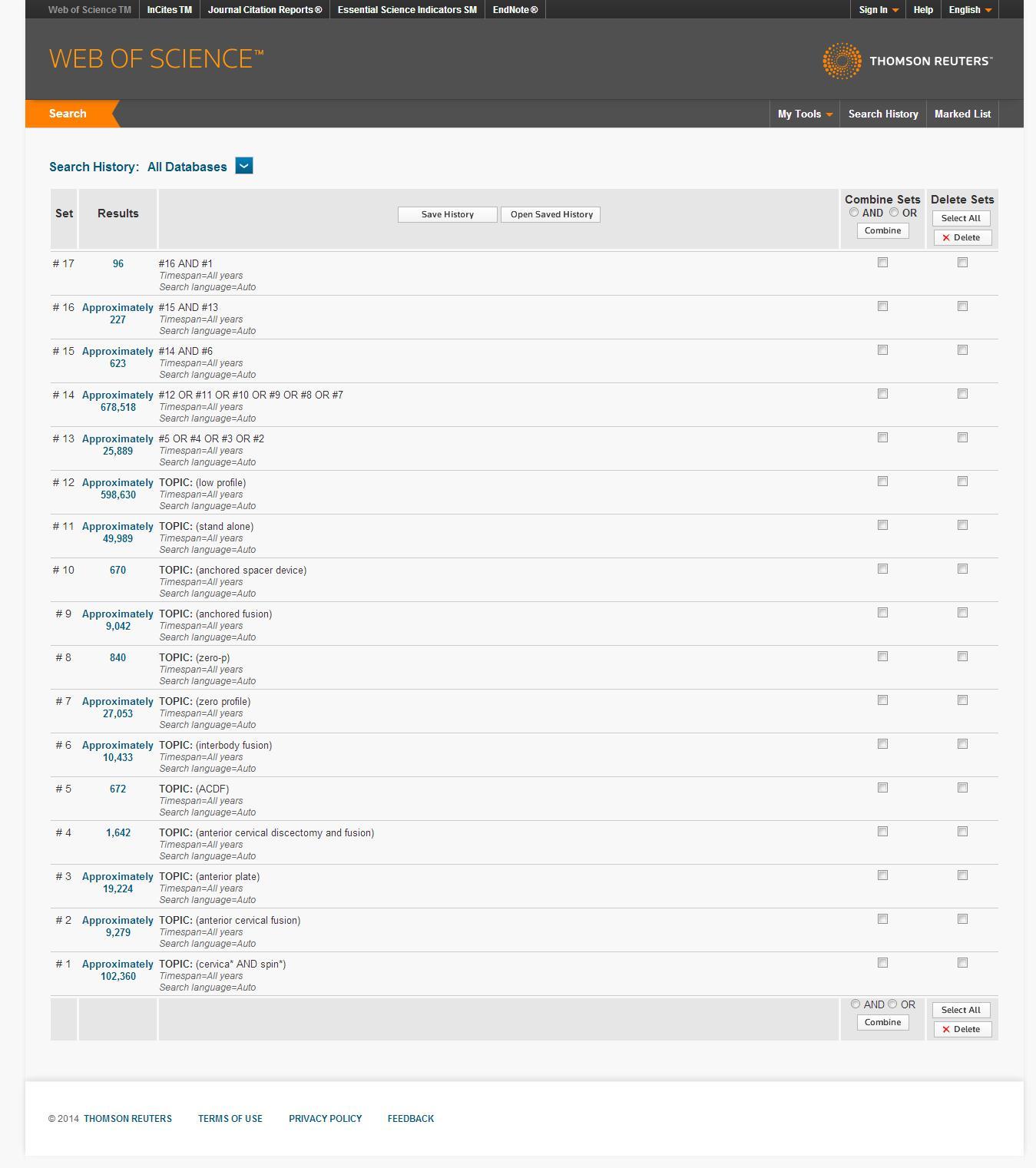

Supplement: S4 Fig — (JPG) [file pone.0130223.s004.jpg]

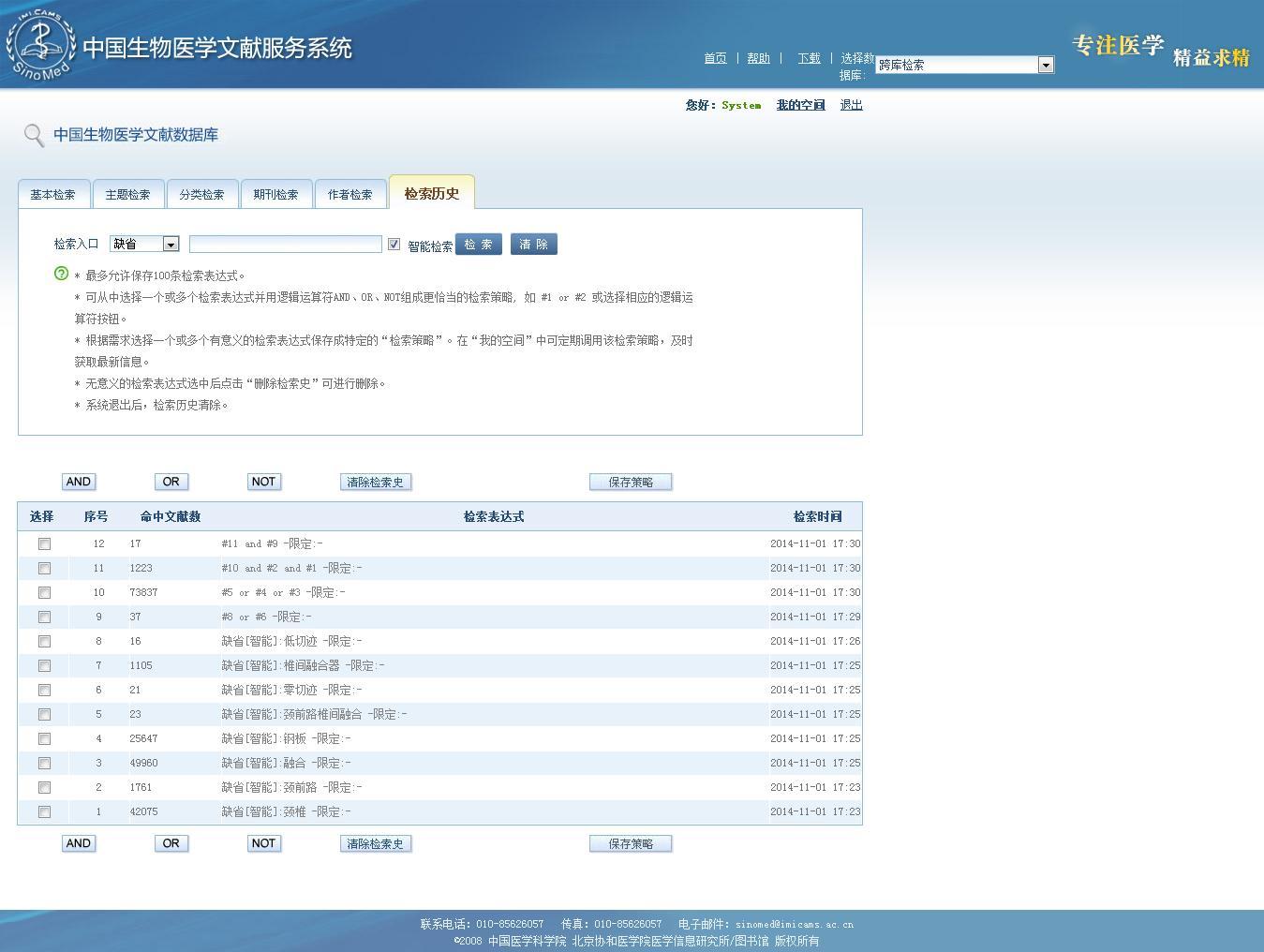

Supplement: S5 Fig — (JPG) [file pone.0130223.s005.jpg]
