# Supplementary material for: Meta-Analysis Comparing Zero-Profile Spacer and Anterior Plate in Anterior Cervical Fusion
Source: PLoS One. 2015 Jun 11;10(6):e0130223. doi: 10.1371/journal.pone.0130223 (PMC4466022; doi:10.1371/journal.pone.0130223)
Supplement: S2 Table — (DOCX) [file pone.0130223.s007.docx]

**S1 Table. Data combining formula**

| Parameter | Group 1 | Group 2 | Combined Data |
| --- | --- | --- | --- |
| Sample size | $n_{1}$ | $n_{2}$ | $n_{1}+n_{2}$ |
| Mean | $\bar{x}_{1}$ | $\bar{x}_{2}$ | $\frac{n_{1}\bar{x}_{1}+n_{2}\bar{x}_{2}}{n_{1}+n_{2}}$ |
| Standard Deviation | SD_1_ | SD_2_ | $\sqrt{\frac{\left( n_{1}-1 \right){SD}^{2}+\left( n_{2}-1 \right){SD}^{2}+\frac{n_{1}n_{2}}{n_{1}+n_{2}}({\bar{x}_{1}}^{2}+{\bar{x}_{2}}^{2}-2\bar{x}_{1}\bar{x}_{2})}{n_{1}+n_{2}-1}}$ |
