# Supplementary material for: Meta-Analysis Comparing Zero-Profile Spacer and Anterior Plate in Anterior Cervical Fusion
Source: PLoS One. 2015 Jun 11;10(6):e0130223. doi: 10.1371/journal.pone.0130223 (PMC4466022; doi:10.1371/journal.pone.0130223)
Supplement: S3 Table — (DOCX) [file pone.0130223.s008.docx]

**S2 Table.** **A method for assessing the quality of a randomlized control trail by Chalmers et al.**

| Quality assessment question | Answer category | Scoring |
| --- | --- | --- |
| **Selection of subjects** |  |  |
| (1) Was there a specific defintion of the diagnosis of this disease in the article? | Yes | 1 star |
|  | No | 0 star |
| (2) Were the selection criteria for the patients in the study specifically described? | Yes | 1 star |
|  | No | 0 star |
| (3) How representative was the control group with respect to the source population of cases enrolled? | Good | 1 star |
|  | Poor | 0 star |
| **Comparability between groups** |  |  |
| (4) Were the groups comparable for…? | Age | 1 star |
|  | Sex | 1 star |
|  | Living area | 1 star |
|  | Ethnicity | 1 star |
| **Outcome presented** |  |  |
| (5) Were all the study results clearly presented? | Yes | 2 stars |
|  | No | 1 star |
