# Supplementary material for: Meta-Analysis Comparing Zero-Profile Spacer and Anterior Plate in Anterior Cervical Fusion
Source: PLoS One. 2015 Jun 11;10(6):e0130223. doi: 10.1371/journal.pone.0130223 (PMC4466022; doi:10.1371/journal.pone.0130223)
Supplement: S4 Table — (DOCX) [file pone.0130223.s009.docx]

**S3 Table.** **The revised and validated version of MINORS**

| Methodological items for non-randomized studies |
| --- |
| 1. **A clearly stated aim**: the question addressed should be precise and relevant in the light of available literature. |
| 2. **Inclusion of consecutive patients**: all patients potentially ﬁt for inclusion (satisfying the criteria for inclusion) have been included in the study during the study period (no exclusion or details about the reasons for exclusion). |
| 3. **Prospective collection of data**: data were collected according to a protocol established before the beginning of the study. |
| 4. **Endpoints appropriate to the aim of the study**: unambiguous explanation of the criteria used to evaluate the main outcome which should be in accordance with the question addressed by the study. Also, the endpoints should be assessed on an intention-to-treat basis. |
| 5. **Unbiased assessment of the study endpoint**: blind evaluation of objective endpoints and double-blind evaluation of subjective endpoints. Otherwise the reasons for not blinding should be stated. |
| 6. **Follow-up period appropriate to the aim of the study**: the follow-up should be sufﬁciently long to allow the assessment of the main endpoint and possible adverse events. |
| 7. **Loss to follow up less than 5%**: all patients should be included in the follow up. Otherwise, the proportion lost to follow up should not exceed the proportion experiencing the major endpoint. |
| 8. **Prospective calculation of the study size**: information of the size of detectable difference of interest with a calculation of 95% conﬁdence interval, according to the expected incidence of the outcome event, and information about the level for statistical signiﬁcance and estimates of power when comparing the outcomes. |
| *Additional criteria in the case of comparative study* |
| 9. **An adequate control group**: having a gold standard diagnostic test or therapeutic intervention recognized as the optimal intervention according to the available published data. |
| 10. **Contemporary groups**: control and studied group should be managed during the same time period (no historical comparison) |
| 11. **Baseline equivalence of groups**: the groups should be similar regarding the criteria other than the studied endpoints of confounding factors that could bias the interpretation of the results. |
| 12. **Adequate statistical analyses**: whether the statistics were in accordance with the type of study with calculation of conﬁdence  intervals or relative risk. |

* The items are scored 0 (not reported), 1 (reported but inadequate) or 2 (reported and adequate). The global ideal score being 16 for non-comparative studies and 24 for comparative studies
