# Supplementary material for: Meta-Analysis Comparing Zero-Profile Spacer and Anterior Plate in Anterior Cervical Fusion
Source: PLoS One. 2015 Jun 11;10(6):e0130223. doi: 10.1371/journal.pone.0130223 (PMC4466022; doi:10.1371/journal.pone.0130223)
Supplement: S5 Table — (DOCX) [file pone.0130223.s010.docx]

**S4 Table. Quality assessment of RCT.**

| Study | **Selection of subjects** | | | **Comparability between groups** | | | | **Outcome presented** | **Score** |
| --- | --- | --- | --- | --- | --- | --- | --- | --- | --- |
|  | (1) | (2) | (3) | Age | Sex | Living area | Ethnicity |  |  |
| **16** | * | * | * | * | * | * | * | ** | 9 |
| **24** | * | * | * | * | * | * | * | ** | 9 |

NOTE: (1) Was there a specific defintion of the diagnosis of this disease in the article? (2) Were the selection criteria for the patients in the study specifically described? (3) How representative was the control group with respect to the source population of cases enrolled?
