# Supplementary material for: Meta-Analysis Comparing Zero-Profile Spacer and Anterior Plate in Anterior Cervical Fusion
Source: PLoS One. 2015 Jun 11;10(6):e0130223. doi: 10.1371/journal.pone.0130223 (PMC4466022; doi:10.1371/journal.pone.0130223)
Supplement: S6 Table — (DOCX) [file pone.0130223.s011.docx]

**S5 Table. Quality assessment of non-randomised studies**

| **Methodological items** | **Study** | | | | | | | |
| --- | --- | --- | --- | --- | --- | --- | --- | --- |
|  | **18** | **17** | **25** | **19** | **26** | **27** | **20** | **28** |
| 1. **A clearly stated aim** | 2 | 2 | 2 | 2 | 2 | 2 | 2 | 2 |
| 2. **Inclusion of consecutive patients** | 1 | 2 | 2 | 2 | 2 | 2 | 2 | 2 |
| 3. **Prospective collection of data** | 0 | 2 | 2 | 0 | 0 | 2 | 0 | 0 |
| 4. **Endpoints appropriate to the aim of the study** | 1 | 2 | 2 | 2 | 2 | 2 | 2 | 2 |
| 5. **Unbiased assessment of the study endpoint** | 0 | 0 | 0 | 0 | 0 | 0 | 0 | 1 |
| 6. **Follow-up period appropriate to the aim of the study** | 2 | 2 | 2 | 2 | 2 | 2 | 2 | 2 |
| 7. **Loss to follow up less than 5%** | 2 | 2 | 2 | 2 | 2 | 2 | 2 | 2 |
| 8. **Prospective calculation of the study size** | 0 | 0 | 0 | 0 | 0 | 0 | 0 | 0 |
| 9. **An adequate control group** | 2 | 2 | 2 | 2 | 2 | 2 | 2 | 2 |
| 10. **Contemporary groups** | 2 | 2 | 2 | 2 | 2 | 2 | 2 | 2 |
| 11. **Baseline equivalence of groups** | 2 | 2 | 1 | 2 | 2 | 2 | 2 | 2 |
| 12. **Adequate statistical analyses** | 2 | 2 | 2 | 2 | 2 | 2 | 2 | 2 |
