# Supplementary material for: Meta-Analysis Comparing Zero-Profile Spacer and Anterior Plate in Anterior Cervical Fusion
Source: PLoS One. 2015 Jun 11;10(6):e0130223. doi: 10.1371/journal.pone.0130223 (PMC4466022; doi:10.1371/journal.pone.0130223)
Supplement: S7 Table — (DOCX) [file pone.0130223.s012.docx]

**S7 Table. Comparison of two models in this manuscript**

| Characteristic | Heterogeneity | | | | Overall effect | |
| --- | --- | --- | --- | --- | --- | --- |
|  | I^2^ (%) | | P value | | P value | |
|  | F | R | F | R | F | R |
| Operation Time | **0** | **0** | **=0.97** | **=0.97** | **<0.00001** | **<0.00001** |
| Blood Loss | **22** | **22** | **=0.27** | **=0.27** | **<0.0001** | **=0.0003** |
| Preoperative JOA Score | **0** | **0** | **=0.79** | **=0.79** | **=0.03** | **=0.03** |
| Postoperative JOA Score | **14** | **14** | **=0.32** | **=0.32** | **=0.11** | **=0.14** |
| Preoperative NDI | **94** | **94** | **<0.00001** | **<0.00001** | **<0.00001** | **0.66** |
| Postoperative NDI | **84** | **84** | **=0.0003** | **=0.0003** | **=0.15** | **=0.31** |
| Preoperative VAS | **61** | **61** | **=0.07** | **=0.07** | **=0.0002** | **=0.06** |
| Postoperative VAS | **97** | **97** | **<0.00001** | **<0.00001** | **<0.0001** | **0.75** |
| Postoperative Segmental Cobb’s Angel | **0** | **0** | **=0.93** | **=0.93** | **<0.0001** | **<0.0001** |
| Postoperative Cervical Cobb’s Angel | **86** | **86** | **<0.00001** | **<0.00001** | **<0.0001** | **0.14** |
| Fusion rate | **0** | **0** | **=0.51** | **=0.51** | **=0.76** | **=0.73** |
| Dysphagia within Six Weeks | **0** | **0** | **0.57** | **0.57** | **=0.002** | **=0.007** |
| Dysphagia at last follow-up | **0** | **0** | **=0.97** | **=0.97** | **=0.0001** | **=0.0004** |

R: Random-effect model; F: Fixed –effect model;
