# Supplementary material for: Meta-Analysis Comparing Zero-Profile Spacer and Anterior Plate in Anterior Cervical Fusion
Source: PLoS One. 2015 Jun 11;10(6):e0130223. doi: 10.1371/journal.pone.0130223 (PMC4466022; doi:10.1371/journal.pone.0130223)
Supplement: S1 File — (ZIP) [file pone.0130223.s013.zip › S8_ZIP. Fve full-text excluded studies and reasons for exclusion/Excluded Study_1.pdf]

Eur Spine J (2013) 22 (Suppl 6):S868–S878
DOI 10.1007/s00586-013-3005-0

REVIEW ARTICLE

Zero-P: a new zero-profile cage-plate device for single and multilevel ACDF. A single Institution series with four years maximum follow-up and review of the literature on zero-profile devices

Giuseppe M. V. Barbagallo · Dario Romano · Francesco Certo · Pietro Milone · Vincenzo Albanese

Received: 14 August 2013 / Revised: 8 September 2013 / Accepted: 8 September 2013 / Published online: 24 September 2013
© Springer-Verlag Berlin Heidelberg 2013

Abstract
Purpose To analyze the prospectively collected data in a series of patients treated with single- or multilevel ACDF with a stand-alone, zero-profile device, focusing on clinico-radiological outcome, complications and technical hints, and to review the literature on such new devices.
Methods Eighty-five patients harboring symptomatic DDD underwent ACDF with the Zero-P cage-plate: 29 at 1-level and 56 at 2–4 levels (total 162 devices). In the multilevel group, 9 patients received a combination of Zero-P and stand-alone cages (hybrid implants). This study focuses on 32 patients with follow-up ranging from 20 to 48 months. NDI, SF-36 and arm pain VAS scores were registered preoperatively and at follow-up visits. Dysphagia was assessed using the Bazaz score. Imaging included X-rays, CT and MRI, also to assess the presence of vertebral body fractures in multilevel cases. Paired Student *t* test was used for statistical analysis.
Results SF-36 and NDI showed a statistically significant improvement ($p < 0.01$) and mean arm pain VAS score decreased from 79 to 41. X-rays and CT demonstrated, respectively, a 94.5 % and a 92 % fusion rate. Three patients complained of moderate and two of mild transient dysphagia (15.5 %). No device-related complications occurred and no fractures, secondary to four screws insertion in one vertebral body (i.e., swiss cheese effect), were detected in multilevel cases. In patients with extensive anterior osteophytes only a "focal spondylectomy" was required.
Conclusion The Zero-P device is safe and efficient, even in multilevel cases. Dysphagia is minimal, extensive anterior osteophytectomy is unnecessary and technical hints may ease the surgical workflow. This is the largest series, with the longest follow-up, reported.

Keywords ACDF · Cage · Cervical spine · Complication · Plate · Zero-P · Zero profile

Introduction

Anterior cervical discectomy and fusion (ACDF) is still regarded as the gold-standard procedure in the treatment of single- and multiple-level cervical disc disease [19, 21, 23] not suitable for artificial disc replacement [34]. Following the long-lasting use of autografts and allografts [2, 7, 8, 12, 15, 24, 36, 39], intervertebral cages, without or with additional anterior cervical plates, became the most commonly used intervertebral devices [1, 14, 25, 27, 30, 39].

Anterior cervical plates may increase interbody fusion rates [6, 10, 16, 35] and stability [16], maintain or improve cervical sagittal alignment [18, 28] and prevent interbody graft dislocation or subsidence [28], particularly in multiple-level ACDFs; however, anterior plating may also be associated with potential disadvantages and complications [17], including increased dysphagia rates [5, 29, 37, 41], tracheoesophageal lesions [31], plate malposition and accelerated adjacent disc degeneration [31], even when low-profile plates are used. Furthermore, in patients harboring spondylotic alterations, such as anterior endplates osteophytes or extensive anterior bony ossification bridging several vertebral bodies, a careful surgical preparation of

G. M. V. Barbagallo (✉) · D. Romano · F. Certo · P. Milone · V. Albanese
Neurosurgery and Radiology Departments, Policlinico "G. Rodolico" University Hospital, Viale XX Settembre 45, 95129 Catania, Italy
e-mail: giuseppebarbagal@hotmail.com

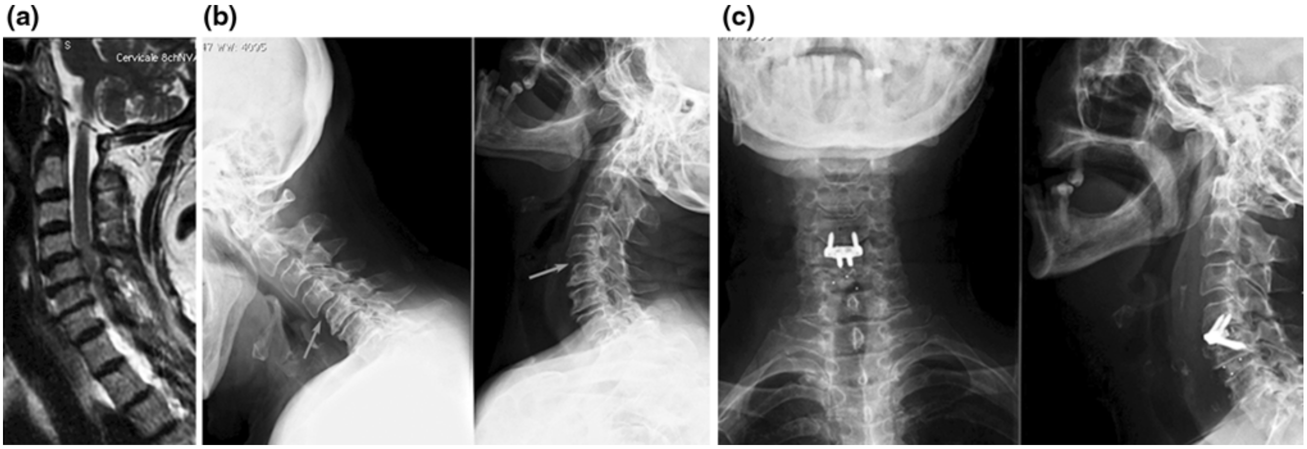

Fig. 1 **a** Sagittal, T2-weighted MRI scan showing degenerative disc disease and spinal cord compression at C4–C5 and C5–C6; signs of instability at C4–C5 are also seen. **b** Lateral flexion–extension X-ray confirming instability at C4–C5. **c** Postoperative X-ray showing a hybrid construct with a Zero-P device at C4–C5 (the unstable level) and a stand-alone CFRP cage at C–C6 level

the anterior surface of the cervical spine, i.e., adequate "flattening" of the bony anterior cervical surface, is required to position the plate.

In the past few years, a new zero-profile, stand-alone device (Zero-P, Synthes GmbH, Switzerland) for ACDF has been developed, with the aim to reduce the morbidity associated with traditional cervical anterior plating, while maintaining the benefits of interbody cages with anterior plating.

We report our experience on a prospectively collected series of patients treated with the Zero-P device, at single or multiple levels, and followed up to 47 months after surgery; indications, tips and tricks related to such new device are described. To our knowledge this is the largest series of patients, with the longest available follow-up, reported in the literature.

Materials and methods

Eighty-five patients (48 males and 37 females), ranging in age from 30 to 74 years (mean age 57 years) and suffering from degenerative disc disease, causing radiculopathy and/or myelopathy unresponsive to conservative treatment, were prospectively enrolled to undergo ACDF with the Zero-P device (DePuy Synthes). One disc level was treated in 29 patients, whereas 56 patients were operated at multiple levels (two to four), for a total of 162 devices implanted. Nine multilevel patients received a hybrid implant, i.e., a combination of Zero-P and stand-alone cages (carbon fiber reinforced polymer-CFRP-cages, DePuy Spine). Degenerative instability was also a suitable indication for surgery with the Zero-P device (Fig. 1).

Table 1 number of disc levels treated per patient

Total number of patients treated	Total levels treated
85	162
Number of patients included in the study	
32	77
1 level	7
2 levels	12
3 levels	6
4 levels	7

Zero-P and CFRP cages were filled with bone substitute, either Actifuse ABX (Baxter) or DBX putty (DePuy Synthes), to enhance intervertebral fusion.

This study analyses a cohort of 32-consecutive patients with the longest follow-up (from 20 to 48 months). Out of these, 25 patients underwent a multilevel procedure, while 7 patients had only a single-level treatment (Table 1). In the multilevel group we included 7 patients treated at 4 levels (C3–C4, C4–C5, C5–C6, C6–C7), 6 at 3 levels (C3–C4, C4–C5, C5–C6), 12 at 2 levels (C4–C5, C5–C6 in 6 patients, C5–C6, C6–C7 in 4 patients, C2–C3 and C3–C4 in 1 patient, C3–C4, C4–C5 in 1 patient) (Fig. 2). In the single-level group we included 4 patients treated at C5–C6, 2 patients at C4–C5 and 1 patient at C3–C4. In the multilevel group, four patients (two operated at two levels and the other two at three levels, respectively) were treated combining Zero-P devices with stand-alone cages: one Zero-P and one stand-alone cage in the two double-level cases and two Zero-P and one stand-alone cage in the other two patients (Fig. 1).

The stand-alone, Zero-P cage-plate is a zero profile, radiolucent PEEK cage integrated with an anterior titanium plate containing four holes with screw treads: the two

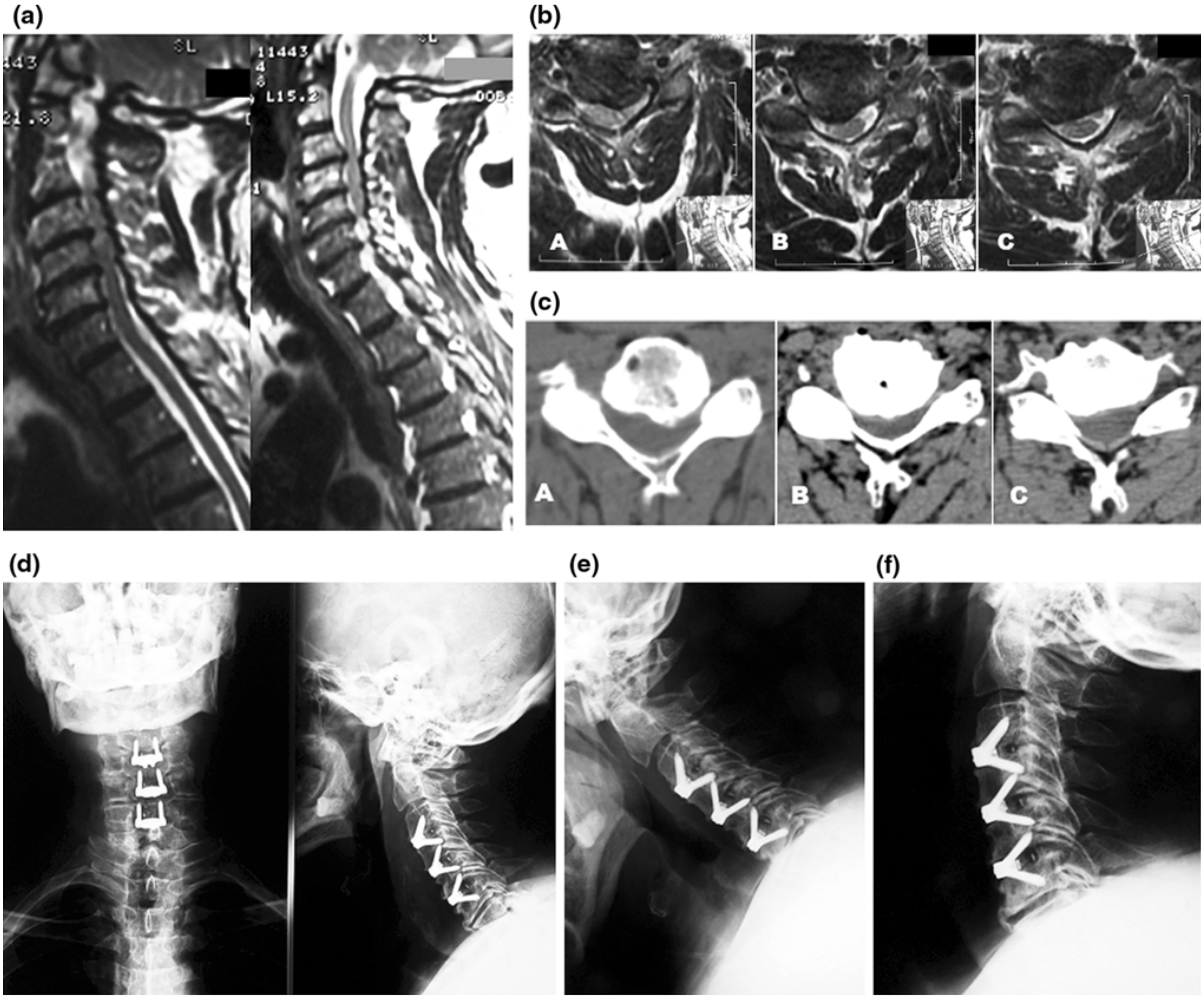

Fig. 2 **a** Sagittal, T2-weighted MRI scan showing degenerative disc disease and spinal cord compression at C3–C4, C4–C5 and C5–C6. Axial MRI (**b**) and CT (**c**) scans demonstrating degenerative changes and neural structures compression, respectively, at C3–C4, C4–C5 and C5–C6. **d** Postoperative AP and lateral X-ray showing a 3-level implant, with good sagittal alignment and satisfactory stability on flexion–extension views (**e**, **f**)

medial ones are designed to place two inferiorly directed screws whereas the lateral holes give passage to the cranial screws. The interface between cage and plate is flexible to allow a better anatomical fit to different anatomical conditions. Cages with different shapes of the superior surface (parallel, convex or lordotic) are available, to be adaptable to the different vertebral body shapes. Tantalum markers are embedded in the cage to check their position with fluoroscopy or X-rays. Dedicated instruments, either straight or angled in shape, are used to insert the cages. In particular, the angled ones are designed to facilitate the screw insertion at the most cranial or caudal (i.e., C2–C3, C3–C4 or C6–C7) levels, or when peculiar anatomical features are present (i.e., patients with short neck, with a mandible hiding the higher disc levels or with a thorax making angulation to access the C6–C7 disc space awkward). Among the instruments, the aiming device should be used to insert the first screw (Fig. 3).

Biomechanically the Zero-P implant is equivalent to a standard cage with plate construct [33].

Inclusion and exclusion criteria were the same commonly accepted for ACDF with interbody cages and anterior cervical plates.

Short-Form (SF-36) and Neck Disability Index (NDI) questionnaires were administered for functional evaluation, and the Visual Analog Scale (VAS) to assess the arm pain. The incidence of dysphagia was recorded using the system proposed by Bazaz et al. [5] (Table 2).

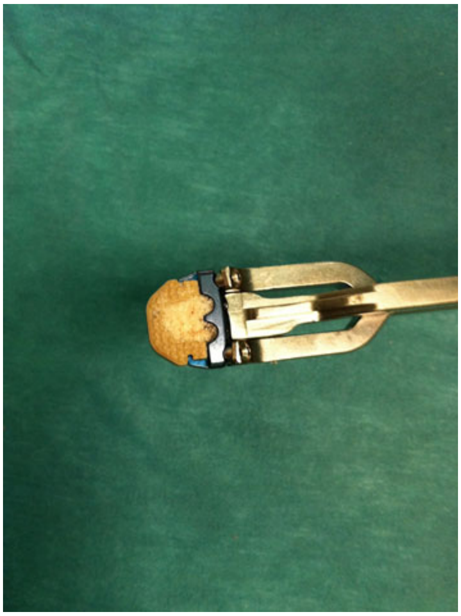

Fig. 3 **a** Zero-P cage-plate device, filled with bone substitute, hold by the aiming device instrument

Table 2 Bazaz grading system for dysphagia

Severity	Liquid	Solid
0-None	None	None
1-Mild	None	Rare
2-Moderate	None or rare	Occasionally
3-Severe	None or rare	Frequent

Preoperative imaging included anterior-posterior (AP) and lateral X-rays, with flexion–extension views, computed tomography (CT) and magnetic resonance imaging (MRI).

All patients were operated by the two senior surgeons (GMVB and VA) using a standard, right-sided, Smith-Robinson technique. After carrying out the microdiscectomy, neural decompression and drilling of the posterior osteophytes compressing the neural structures, resection of the posterior longitudinal ligament was performed. The cartilaginous endplate was thoroughly scraped off with a periosteal elevator to enhance the intervertebral fusion process. The right device was then selected after trialing the most appropriate spacer in size and shape (i.e., convex versus lordotic cage endplate). Fluoroscopy was used intraoperatively to check the correct device's placement (i.e., the appropriate depth on lateral view) and screws insertion angle.

Postoperatively, X-rays were obtained at each follow-up visit to assess the position of the implanted devices and the fusion rate. Radiographic interbody fusion was accepted if no radiolucencies could be detected in the graft-endplate area and bridging trabeculation was seen [28, 34].

Table 3 Epidemiological data of studied patients

Patients	Age	Diagnosis	Treated levels
1	74	Myeloradiculopathy	C3–C4, C4–C5, C5–C6
2	70	Myeloradiculopathy	C3–C4, C4–C5, C5–C6
3	30	Radiculopathy	C5–C6
4	44	Radiculopathy	C4–C5
5	76	Myeloradiculopathy	C4–C5, C5–C6
6	54	Myeloradiculopathy	C3–C4, C4–C5, C5–C6, C6–C7
7	69	Myeloradiculopathy	C4–C5, C5–C6
8	45	Myeloradiculopathy	C5–C6, C6–C7
9	74	Myeloradiculopathy	C3–C4, C4–C5, C5–C6, C6–C7
10	70	Myeloradiculopathy	C3–C4, C4–C5, C5–C6
11	50	Myeloradiculopathy	C4–C5, C5–C6
12	66	Myeloradiculopathy	C4–C5, C5–C6
13	42	Radiculopathy	C5–C6
14	50	Myeloradiculopathy	C3–C4, C4–C5, C5–C6, C6–C7
15	54	Myeloradiculopathy	C3–C4, C4–C5, C5–C6, C6–C7
16	52	Myeloradiculopathy	C5–C6, C6–C7
17	51	Myeloradiculopathy	C4–C5, C5–C6
18	40	Radiculopathy	C5–C6
19	70	Myeloradiculopathy	C3–C4, C4–C5, C5–C6
20	55	Myeloradiculopathy	C3–C4, C4–C5, C5–C6, C6–C7
21	62	Myeloradiculopathy	C3–C4
22	75	Myeloradiculopathy	C2–C3, C3–C4
23	44	Myeloradiculopathy	C3–C4, C4–C5
24	46	Myeloradiculopathy	C3–C4, C4–C5, C5–C6
25	70	Myeloradiculopathy	C4–C5
26	35	Myeloradiculopathy	C5–C6, C6–C7
27	48	Myeloradiculopathy	C4–C5, C5–C6
28	42	Myeloradiculopathy	C5–C6, C6–C7
29	71	Myelopathy	C5–C6
30	54	Myeloradiculopathy	C3–C4, C4–C5, C5–C6, C6–C7
31	69	Myeloradiculopathy	C3–C4, C4–C5, C5–C6
32	63	Myeloradiculopathy	C3–C4, C4–C5, C5–C6, C6–C7

CT scans were performed after surgery with the aim to confirm neural decompression and to specifically rule out in multilevel cases vertebral body fractures or significant bony alterations secondary to the insertion of four screws (two belonging to the cranial Zero-P and two to the caudal device) in the intervening vertebra.

Postoperative MRI scans documented the neural decompression and also the quality of images in patients treated with the Zero-P device.

Functional and radiological evaluations, respectively, were performed independently by two neurosurgeons (DR, FC) and one radiologist (PM). Statistical analysis was performed using the paired Student t test and a p value of <0.05 was accepted as statistically significant.

After surgery, patients were assessed at 6 weeks, 3, 6, 12 months and then yearly.

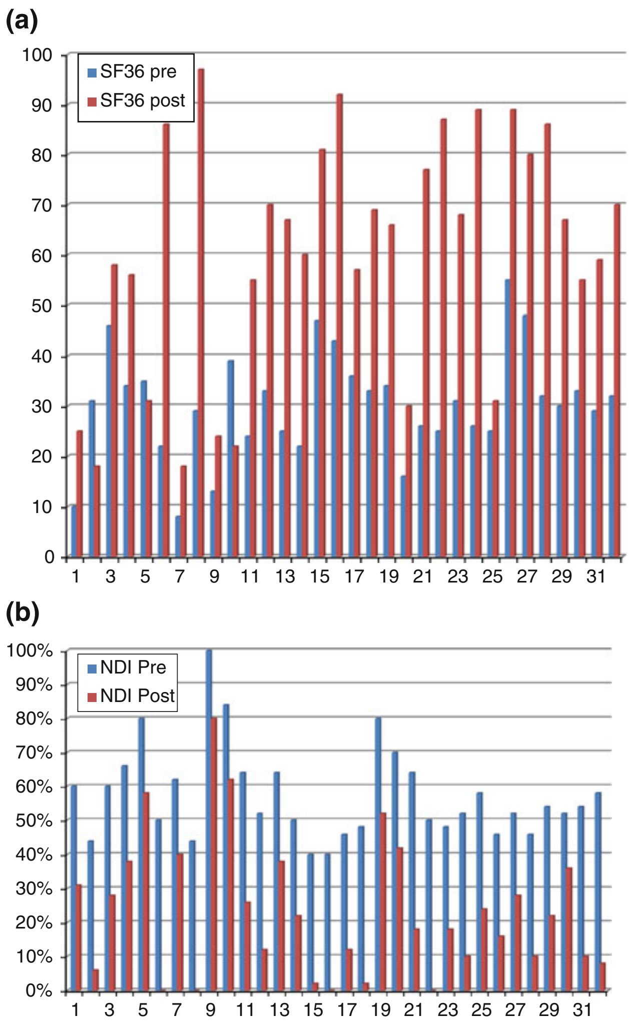

Fig. 4 **a** Comparison between pre- and postoperative (last follow-up) SF-36 mean score. **b** Comparison between pre- and postoperative (last follow-up) NDI mean score

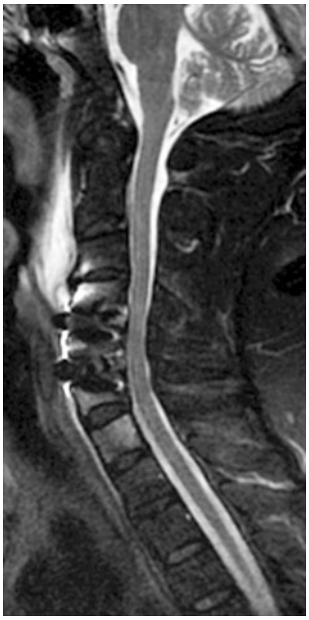

Fig. 5 Postoperative, sagittal T2-weighted MRI showing the lack of artefacts and a nice view of the spinal cord with a CSF film around it

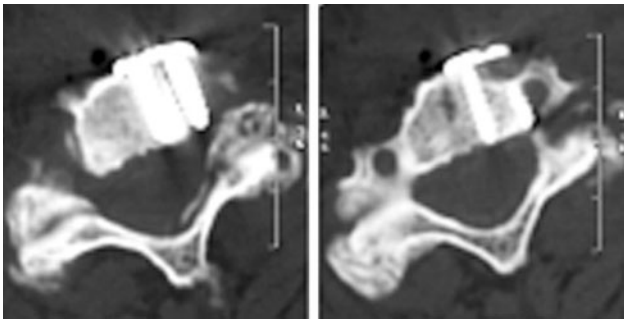

Fig. 6 An off-side positioned Zero-P device, with one screw encroaching the foramen transversarium

Results

In the selected cohort of 32 patients maximum follow-up ranges from 20 to 48 months (mean 27.3 months). One disc level was treated in 7 patients, two disc levels in 12 patients, three levels in 6 cases and four levels in 7 patients (Table 3).

The mean SF-36 score ranges from 30.37 % before surgery to 60.62 % at latest follow-up. Mean NDI improved from 57 % preoperatively to 23 % at last follow-up (Fig. 4a, b). According to such values, both SF-36 and NDI postoperative scores are statistically significant ($p < 0.01$).

Mean arm pain VAS score decreased from 79, before surgery, to 41 postoperatively, and such positive trend was maintained at following follow-up visits.

AP and lateral postoperative X-rays were available in all patients, whereas CT and MRI studies were only performed in 24 and 20 patients, respectively, who consented such additional imaging follow-up. On X-rays a 94.5 % fusion rate was achieved at last follow-up, whereas on thin-cut slice, reformatted sagittal CT scans a 92 % fusion rate was detected.

Interestingly, on MRI the Zero-P device did not cause significant image artifacts and allowed a satisfactory evaluation of the achieved neural decompression (Fig. 5).

No device-related complications were encountered; however, the following complications occurred: one post-operative hematoma requiring surgical evacuation; one screw displacement 1 month after surgery in a patient treated for many years with steroids for a renal disease and with poor bone quality; one case of device malposition with a screw encroaching the lateral surface of the cranial

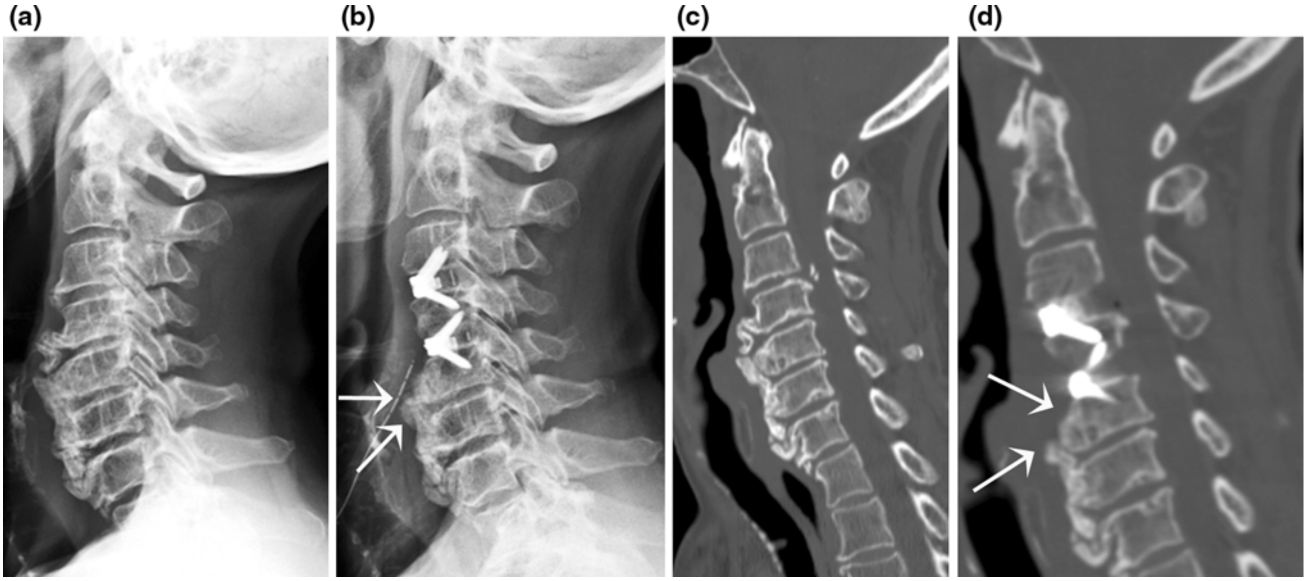

Fig. 7 Extensive anterior osteophytes are present at all spinal levels on lateral X-ray (**a**), mostly at C4–C5, C5–C6 and C6–C7. The Zero-P device allows a "focal spondylectomy" (**b**), which is required to enter the disc level and also to insert screws through the Zero-P plate using dedicated instruments with the right angulation (*cranial arrow*). Sagittal, reconstructed CT scan (**c**, **d**) demonstrate the resected osteophyte (*cranial arrow*) and the remaining anterior osteophyte (*caudal arrow*), which would prevent the implantation of an anterior cervical plate

vertebral body towards the foramen transversarium (Fig. 6).

Postoperatively, according to the Bazaz criteria, a moderate dysphagia was reported in three patients (9.3 %), two operated at one level and one at four levels, and a mild dysphagia in other two cases (6.2 %). Nonetheless, in all patients dysphagia was transient, improved spontaneously and was no longer present at the first follow-up appointment.

It should be highlighted that in patients showing anterior vertebral osteophytes, we performed only a "focal spondylectomy". This is a partial and focal osteophyte removal, either on the anterior cranial or caudal vertebral body surface, needed to properly angulate the instruments (awl, tap, screwdriver) used to prepare the screws site and then insert them.

Postoperatively, patients did not wear any cervical collar.

Discussion

Over the last 20 years, an increasingly growing experience with anterior cervical plates in patients undergoing ACDF has led to the acknowledgment of either several advantages or complications. The former include a higher fusion rate, particularly in multilevel cases, a better alignment of the cervical spine, with restoration or maintenance of lordosis, and the prevention of interbody graft/cage dislocation or subsidence [28]. Among the latter, dysphagia and tracheoesophageal lesions are the most frequently reported [9, 31]. Yet, asymmetry of the anterior surface of the cervical spine, related to the presence of osteophytes in patients with spondylosis or to minor spondylolisthesis secondary to degenerative changes or trauma, is another condition requiring careful consideration before implanting a plate (Figs. 1, 7).

Song et al. [35] compared two groups of patients treated by ACDF, respectively, with cage alone and with cage and plate. They found that in 1- and 2-level ACDF the use of a plate was associated with a better sagittal alignment, higher fusion rate and lower cage subsidence and complication rates; however, the authors did not report significant differences in clinical outcome between the two groups.

Pitzen et al. [28] analyzed the differences in fusion rates, complications and changes in segmental lordosis in patients treated with dynamic plates versus rigid ones. They found that when a dynamic plate is used the fusion process is faster and the implant-related complications are lower. These advantages, however, are counterbalanced by a greater loss of segmental lordosis at 2-year follow-up than that observed in patients treated with a rigid plate. Because such difference in segmental lordosis is not associated with significant clinical differences, and the risk of dynamic plate-related complications is lower, they suggested that a dynamic plate should be considered the first-choice device.

In the present series, the clinical and neurological improvement reported at 6-week and 6-month follow-up is

maintained at the last visit, and this compares favorably with data reported in the literature on zero-profile devices [13, 22, 34].

The radiological evaluation of all imaging studies (X-rays with flexion–extension views, CT and MRI scans) confirmed a good sagittal alignment, a 94.5 % X-ray fusion rate at last follow-up and a satisfactory neural decompression. Interestingly, no patients treated with Zero-P at more than one level showed on postoperative CT scans signs of vertebral fractures due to the insertion of four screws in one single vertebra.

Moreover, anterior vertebral osteophytes did not require an extensive preparation of the anterior surface of the vertebral bodies, with bony spurs removal or flattening as it is the case with cervical plates, but rather a "focal spondylectomy" just along the trajectory needed to insert the instruments in the cage's anterior holes and fix the Zero-P device to the adjacent vertebral bodies (Fig. 7). And this appears as an obvious technical advantage, with reduced surgical time.

Another advantage associated with Zero-P is the reduced risk of inducing adjacent disc level degeneration and spondylotic changes; it has been shown that cervical plates reaching the adjacent disc levels can induce and accelerate disc degeneration and osteophyte formation, leading to future complications [26]. Conversely, Zero-P minimizes such risk as it remains within the index disc space, far from adjacent-level disc spaces; and correct plate position (i.e., not reaching the adjacent disc space) has been shown to be associated with a lower incidence of adjacent-level degeneration [40]. Nonetheless, Miao et al. [22] pointed out that a longer observation is necessary to determine whether or not these devices can also reduce the rate of adjacent segment degeneration.

Postoperative dysphagia is a common event after ACDF with and without plating, occurring with a frequency ranging from 2 to 67 % [5, 37]. In our experience the Zero-P device was associated with a reduced rate of dysphagia (only two cases of mild and three of moderate dysphagia) in the postoperative course (15.5 %); indeed, the Zero-P is contained within the disc space and does not protrude past the anterior wall of the vertebral body, avoiding a direct contact with the tracheoesophageal soft tissues. In particular, the zero profile of the device is fundamental to avoid a mechanical irritation of the esophagus, one of the known main device-related causes of chronic dysphagia [20]. It should be pointed out that in our series the three patients suffering from postoperative moderate dysphagia had undergone surgery at C4–C5 or C5–C6, either as single- or multiple-level surgery. Such experience is similar to that reported by Tortolani et al. [37], although other studies suggest that the more cranial the operated disc level (i.e., C3–C4) the higher is the risk of postoperative dysphagia, or just fail to demonstrate any correlation with the level of surgery [5, 11, 41]. Even the thickness of plates has been correlated with the dysphagia rate, this being lower when thin, low-profile plates are used [20].

Indeed, such reasoning on the role of anterior plates as a main cause of dysphagia should be interpreted cautiously, as other well-known factors, like esophageal retraction or edema, injury of the esophageal nerve plexus or of the superior laryngeal nerve, prevertebral soft-tissues swelling and graft protrusion may also play a role in inducing swallowing difficulties [37].

Yue et al. analyzed the occurrence of persistent voice problems and dysphagia in a cohort of 74 patients who underwent ACDF with allograft and anterior plating. They observed that at a mean 7-year follow-up (ranging from 5 to 11 years), dysphagia was present in 35.1 % of patients. However, no correlation between onset of dysphagia and sex, age, smoking status, duration of surgery, number of levels, plating system implanted or previous anterior neck surgery was disclosed [41]. Such experience is clearly different from ours and others' [13]. Either our five patients who suffered from postoperative dysphagia or the ones in Hofstetter et al.'s [13] series, who were operated using an LDR device, reported improvement of their swallowing difficulties at last follow-up.

A temporal relation between surgery and improvement of dysphagia as reported by patients has been also documented by other authors: Bazaz et al. [5] described a 50 % incidence of dysphagia at 1 month postoperatively but only 12.5 % of their patients were still symptomatic at 1-year follow-up. Conversely, 21.3 % of patients in Riley's et al. [29] series still reported dysphagia at 24-month follow-up.

The common experience of postoperative dysphagia, albeit of different severity, and the high incidence rate reported by several authors may lead to the hypothesis that minor postoperative swallowing difficulties, secondary to different but not obvious etiological mechanisms, could also be considered as a so-called "irritative" non-pathological phenomenon associated with anterior cervical spine surgery, provided that it settles down by 6 weeks or, at latest, 3 months after surgery.

The new zero-profile devices allow a reduced rate of dysphagia, as we could experience and is reported by other authors [13, 22, 34]. The Zero-P implant, whose biomechanical features appear to be similar to those of the cage plus plate system [34], has also been associated with similar clinical and radiological results compared to those of classical ACDF with cage and plating [13].

We have implanted the Zero-P in single and multilevel surgeries. In the latter group, we also used a "hybrid" technique in 9 patients: 7 treated for a two-level disease and 2 patients for a three-level surgery. In these patients we combined one Zero-P and one CFRP cage in the two-level

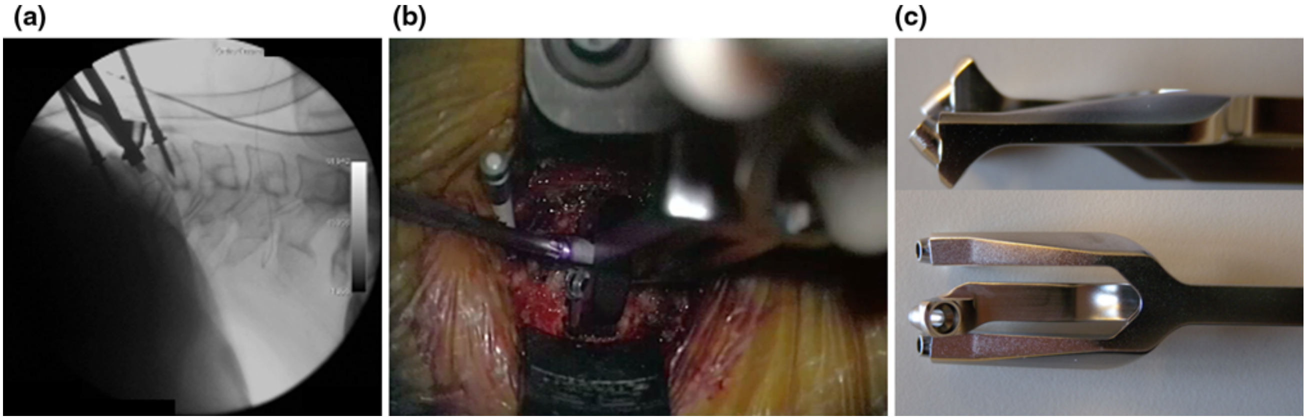

Fig. 8 **a** Fluoroscopic picture showing a conflict between the vertebral body distractor's pin and the screwdriver inserted through the aiming device instrument. This is shown also with an intraoperative picture (**b**); a custom-made aiming device, with a smaller "nose" (**c**) can be helpful to reduce such technical problem

cases or two Zero-P and one CFRP cage in the three-level cases. CFRP cages were implanted in those disc spaces which were less easily accessible (i.e., C3−C4 or C6−C7) because of specific patients' anatomic peculiarities, despite the availability of angled instruments, or when the surgeon deemed not necessary to enhance cage fixation with screws at all treated levels; indeed, in two-level surgeries with only one level unstable on flexion–extension X-ray because of degenerative changes, the Zero-P was used at that level and a CFRP cage in the adjacent and stable level (Fig. 1). Interestingly, in the selected cohort of patients herein analyzed only 4 patients underwent such treatment (2 double level and 2 three levels) and follow-up X-rays evaluation did not show differences in fusion rate between the two different devices used in the same patient and also in comparison to other patients treated with Zero-P only devices. Yet, combination of different devices in a hybrid construct in the cervical spine can be a safe and effective solution, as we also reported in the association of arthrodesis and arthroplasty [4].

The experience with multilevel cases allowed us to understand the following technical hints:

- the vertebral body distractor pins should be placed offset, to avoid any mechanical conflict with the aiming device instrument (Fig. 8). In single-level cases, after inserting the device, the pin can be removed should any difficulty with awl, tap or screwdriver arise; differently, in multilevel procedures if the pin is removed because of the above difficulties with instruments, microdiscectomy and neural decompression at the next adjacent level would require its re-positioning to properly open up the intervertebral space. Alternatively, a customized aiming device, with a shorter "nose" (Fig. 8), can be used to avoid a mechanical conflict with the distractor pins;
- in cases of extensive anterior spondylosis or diffuse idiopathic skeletal hyperostosis (DISH), with large osteophytes arising from the anterior endplates and covering the disc space, it is not required a thorough removal of osteophytes, but rather a "focal spondylectomy". This is necessary either to access the disc space or to insert the instruments with the right angulation into the plate holes (Figs. 7, 9);
- the cage is available only in one size in width. Nonetheless, if it is placed between the bases of the two uncus it will be centered in line with the vertebral body's midline. In particular, if one uncinate process needs a more extensive removal to carry out an adequate foraminal nerve root decompression, it is important to align the Zero-P with the contralateral uncus. Such technical hint, which has been proven to be reliable also when using other devices [3], is helpful to avoid misplacing one of the lateral screws towards the vertebral artery, as we experienced in one case (Fig. 6). Yet, even if it is possible to check the mediolateral rotation of the device on AP fluoroscopy, usually this is not needed and a minor rotation does not seem to cause any problem;
- the screws augmentation prevented any cage's subsidence and the zero profile did not induce soft-tissues irritation.

Regardless of the number of operated levels, we did not encounter implant-related complications during follow-up. Such finding is significantly different from the literature data, which report a failure rate of up to 71 %, particularly for multilevel plate-augmented cervical reconstructions [32]. Indeed, Vaccaro et al. [38] reported an incidence of screw and plate loosening reaching 15.4 %, screw and plate breakage rates, respectively, up to 13.3 and 6.7 %, a

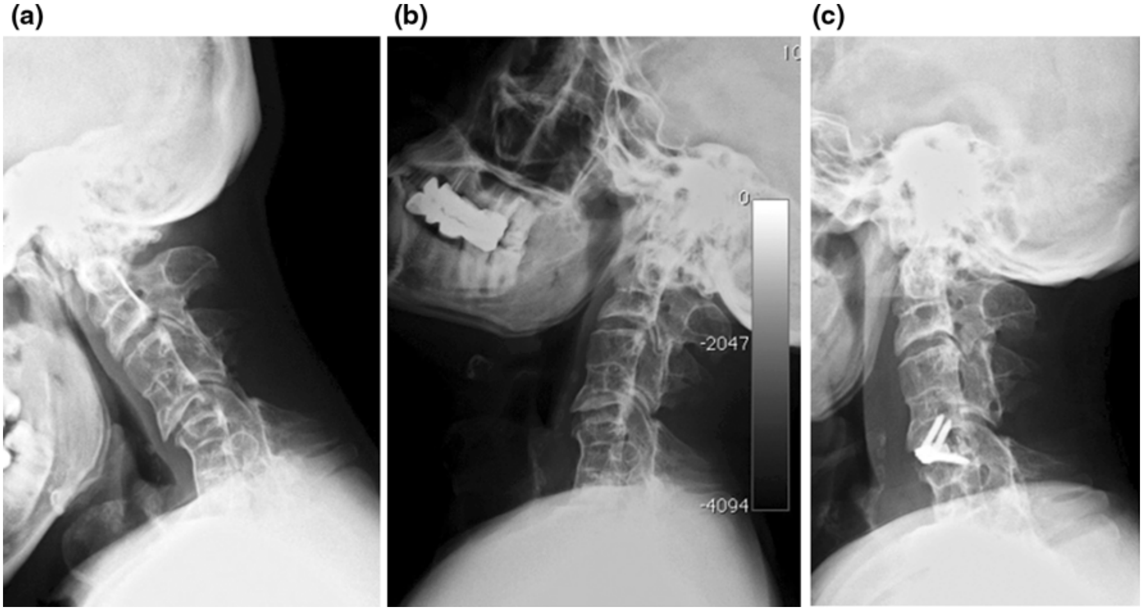

Fig. 9 **a** Flexion–extension lateral X-ray showing a C4–C5 instability in a patient with DISH. **b** A Zero-P device has been used to fix the unstable segment

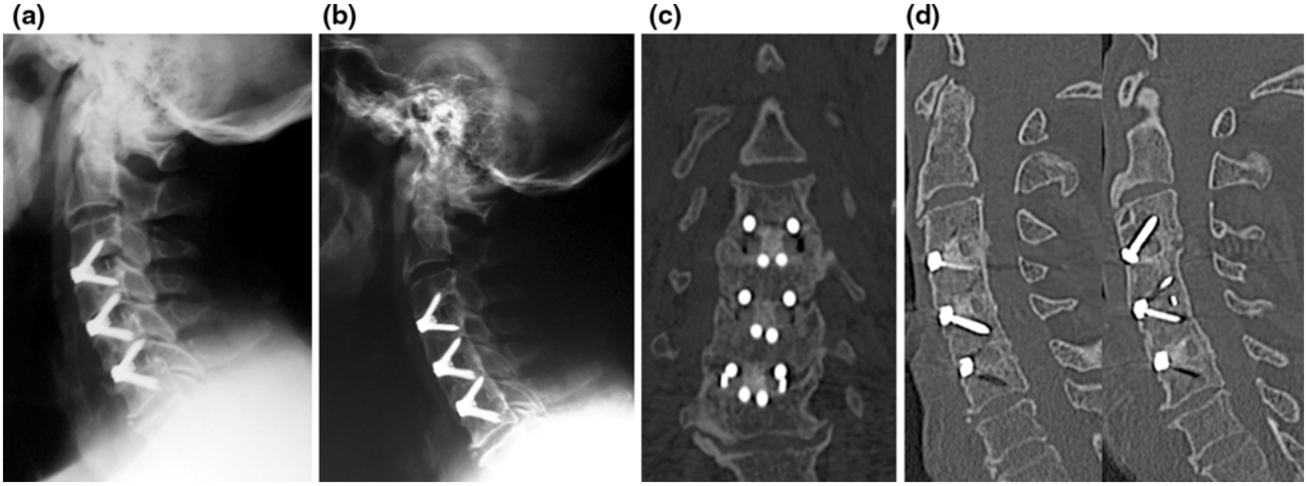

Fig. 10 **a** Lateral X-ray highlighting a 3-level fixation. 2-year follow-up X-ray. **b** Showing a solid fusion through the Zero-P cages, as also seen on reconstructed coronal (**c**) and sagittal (**d**) CT scan

plate and graft displacement rate scaling up to 21.4 % and an incidence of implant malposition from 0 to 12.5 % for long-segment plates.

Moreover, the present study reports our experience in anterior three- and four-level surgeries performed in six and seven patients, respectively, confirming the efficacy of the Zero-P device either in improving clinical symptoms or in maintaining or restoring the cervical lordosis, and the lack of those specifically device-related complications allegedly associated with multilevel cases and currently available zero-profile devices (i.e., Swiss cheese effect in the intervening vertebrae) (Fig. 10).

In conclusion, we are aware of the inherent limitations of our study: no control group (graft/cage and plate) has been included for comparison, the number of evaluated patients is small and a longer follow-up is advisable. Nonetheless, this study demonstrates the safety and efficacy of the Zero-P device in a subgroup of the largest series of patients treated at single and multiple levels and with the longest available follow-up.

Acknowledgments The authors declare that no funding has been or will be received from any grant-giving organization in conjunction with the generation of this manuscript.

Conflict of interest None.

References

1. Agrillo U, Mastronardi L, Puzzilli F (2002) Anterior cervical fusion with carbon fiber cage containing coralline hydroxyapatite: preliminary observations in 45 consecutive cases of soft-disc herniation. J Neurosurg 96(3 Suppl):273–276
2. Bagby GW (1995) Cages mètalliques intersomatiques filatèes pour arthrodesis rachidiennes. In: Duparc J, Schreiber A, Troisier O (eds) Instabilitès vertèbrales lombaires. Expansion Scientifique Francaise, Paris, pp 199–213
3. Barbagallo GM, Corbino LA, Papavero L, Fritzsche E, Albanese V (2009) Anatomic midline marking during cervical arthroplasty with ProDisc-C: an alternative, simple, and reliable method: technical note. J Spinal Disord Tech 22(8):610–614. doi:10.1097/BSD.0b013e31819b0a6f
4. Barbagallo GM, Assietti R, Corbino L, Olindo G, Foti PV, Russo V, Albanese V (2009) Early results and review of the literature of a novel hybrid surgical technique combining cervical arthrodesis and disc arthroplasty for treating multilevel degenerative disc disease: opposite or complementary techniques? Eur Spine J 18(Suppl 1):29–39. doi:10.1007/s00586-009-0978-9
5. Bazaz R Lee MJ, Yoo JU (2002) Incidence of dysphagia after anterior cervical spine surgery: a prospective study. Spine (Phila Pa 1976*)* 27:2453-2458
6. Bohler J, Gaudernak T (1980) Anterior plate stabilization for fracture-dislocation of the lower cervical spine. J Trauma 20:203–205
7. Epstein NE (2012) Iliac crest autograft versus alternative constructs for anterior cervical spine surgery: pros, cons, and costs. Surg Neurol Int 3(Suppl 3):S143–S156. doi:10.4103/2152-7806.98575
8. Fernyhough JC, White JI, LaRocca H (1991) Fusion rates in multilevel cervical spondylosis comparing allofraft fibula with autograft fibula in 126 patients. Spine 16:561–565
9. Fountas KN, Kapsalaki EZ, Nikolakakos LG, Smisson HF, Johnston KW, Grigorian AA, Lee GP, Robinson JS Jr (2007) Anterior cervical discectomy and fusion associated complications. Spine (Phila Pa 1976) 32:2310–2317
10. Fraser JF, Härtl R (2007) Anterior approaches to fusion of the cervical spine: a metaanalysis of fusion rates. J Neurosurg Spine 6(4):298–303
11. Frempong-Boadu A, Houten JK, Osborn B, Opulencia J, Kells L, Guida DD, Le Roux PD (2002) Swallowing and speech dysfunction in patients undergoing anterior cervical discectomy and fusion: a prospective, objective preoperative and postoperative assessment. J Spinal Disord Tech 15(5):362–368
12. Friedlaender GE, Huo M (1991) Bone graft and bone substitutes. In: Frymoyer JW (ed) The adult spine: principles and practice. Raven, New York, pp 565–754
13. Hofstetter C, Kesavabhotla K, Boockvar JA (2013) Zero-Profile anchored spacer reduces rate of dysphagia compared to ACDF with anterior plating. J Spinal Disord Tech. [Epub ahead of print]
14. Hwang SL, Lin CL, Lieu AS, Lee KS, Kuo TH, Hwang YF, Su YF, Howng SL (2004) Three-level and four-level anterior cervical discectomies and titanium cage-augmented fusion with and without plate fixation. J Neurosurg Spine 1(2):160–167
15. Jacobs W, Willems PC, Kruyt M, van Limbeek J, Anderson PG, Pavlov P, Bartels R, Oner C (2011) Systematic review of anterior interbody fusion techniques for single- and double-level cervical degenerative disc disease. Spine (Phila Pa 1976) 36(14):E950–E960. doi:10.1097/BRS.0b013e31821cbba5
16. Kaiser MG, Haid RW Jr, Subach BR, Barnes B, Rodts GE Jr (2002) Anterior cervical plating enhances arthrodesis after discectomy and fusion with cortical allograft. Neurosurgery 50:229–236
17. Kasimatis GB, Panagiotopoulos E, Gliatis J, Tyllianakis M, Zouboulis P, Lambiris E (2009) Complications of anterior surgery in cervical spine trauma: an overview. Clin Neurol Neurosurg 111:18–27
18. Kim SW, Limson MA, Kim SB, Arbatin JJ, Chang KY, Park MS, Shin JH, Ju YS (2009) Comparison of radiographic changes after ACDF versus Bryan disc arthroplasty in single and bi-level cases. Eur Spine J 18:218–231
19. Korinth MC (2008) Treatment of cervical degenerative disc disease: current status and trends. Zentralbl Neurochir 69:113–124
20. Lee MJ, Bazaz R, Furey CG, Yoo J (2005) Influence of anterior cervical plate design on Dysphagia: a 2-year prospective longitudinal follow-up study. J Spinal Disord Tech 18(5):406–409
21. Matz PG, Ryken TC, Groff MW, Vresilovic EJ, Anderson PA, Heary RF, Holly LT, Kaiser MG, Mummaneni PV, Choudhri TF, Resnick DK, Joint Section on Disorders of the Spine and Peripheral Nerves of the American Association of Neurological Surgeons and Congress of Neurological Surgeons (2009) Technique for anterior cervical decompression for radiculopathy. J. Neurosurg Spine 11(2):183–197
22. Miao J, Shen Y, Kuang Y, Yang L, Wang X, Chen Y, Chen D (2012) Early follow-up outcomes of a new zero-profile implant used in anterior cervical discectomy and fusion. J Spinal Disord Tech. [Epub ahead of print]
23. Mummaneni PV, Kaiser MG, Matz PG, Anderson PA, Groff MW, Heary RF, Holly LT, Ryken TC, Choudhri TF, Vresilovic EJ, Resnick DK, Joint Section on Disorders of the Spine and Peripheral Nerves of the American Association of Neurological Surgeons and Congress of Neurological Surgeons (2009) Cervical surgical techniques for the treatment of cervical spondylotic myelopathy. J Neurosurg Spine 11(2):130–141
24. Muthukumar N (2012) Surgical management of cervical spondylotic myelopathy. Neurol India 60(2):201–209. doi:10.4103/0028-3886.96402
25. Niu CC, Liao JC, Chen WJ, Chen LH (2010) Outcomes of interbody fusion cages used in 1 and 2-levels anterior cervical discectomy and fusion: titanium cages versus polyetheretherketone (PEEK) cages. J Spinal Disord Tech 23(5):310–316. doi:10.1097/BSD.0b013e3181af3a84
26. Park JB, Cho YS, Riew KD (2005) Development of adjacent-level ossification in patients with an anterior cervical plate. J Bone Joint Surg Am 87:558–563
27. Peolsson A (2007) Investigation of clinically important benefit of anterior cervical decompression and fusion. Eur Spine J 16(4):507–514
28. Pitzen TR, Chrobok J, Stulik J, Ruffing S, Drumm J, Sova L, Kucera R, Vyskocil T, Steudel WI (2009) Implant complications, fusion, loss of lordosis, and outcome after anterior cervical plating with dynamic or rigid plates: two year result of a multicentric, randomized, controlled study. Spine (Phila Pa 1976) 34(7):641–646
29. Riley LH 3rd, Skolasky RL, Albert TJ, Vaccaro AR, Heller JG (2005) Dysphagia after anterior cervical decompression and fusion. Spine 30(22):2564–2569
30. Ryken TC, Heary RF, Matz PG, Anderson PA, Groff MW, Holly LT, Kaiser MG, Mummaneni PV, Choudhri TF, Vresilovic EJ,

Resnick DKJ, Joint Section on Disorders of the Spine and Peripheral Nerves of the American Association of Neurological Surgeons and Congress of Neurological Surgeons (2009) Techniques for cervical interbody grafting. J Neurosurg Spine 11(2):203–220
31. Sahjpaul RL (2007) Esophageal perforation from anterior cervical screw migration. Surg Neurol 68(2):205–209
32. Sasso RC, Ruggiero RA Jr, Reilly TM, Hall PV (2003) Early reconstruction failures after multilevel cervical corpectomy. Spine (Phila Pa 1976) 28:140–142
33. Scholz M, Reyes PM, Schleicher P, Sawa AG, Baek S, Kandziora F, Marciano FF, Crawford NR (2009) A new stand-alone cervical anterior interbody fusion device. Spine 34(2):156–160
34. Scholz M, Schnake KJ, Pingel A, Hoffmann R, Kandziora F (2011) A new zero-profile implant for stand-alone anterior cervical interbody fusion. Clin Orthop Relat Res 469:666–673
35. Song KJ, Taghavi CE, Lee KB, Song JH, Eun JP (2009) The efficacy of plate construct augmentation versus cage alone in anterior cervical fusion. Spine (Phila Pa 1976) 34:2886-2892
36. Thalgott JS, Fritts K, Giuffre JM, Timlin M (1999) Anterior interbody fusion of the cervical spine with coralline hydroxyapatite. Spine 24:1295–1299
37. Tortolani PJ, Cunningham BW, Vigna F, Hu N, Zorn CM, McAfee PC (2006) A comparison of retraction pressure during anterior cervical plate surgery and cervical disc replacement: a cadaveric study. J Spinal Disord Tech 19:312–317
38. Vaccaro AR, Falatyn SP, Scuderi GJ, Eismont FJ, McGuire RA, Singh K, Garfin SR (1998) Early failure of a long segment anterior cervical plate fixation. J Spinal Disord 11:410–415
39. Vavruch L, Hedlund R, Javid D, Leszniewski W, Shalabi A (2002) A prospective randomized comparison between the Cloward procedure and a carbon fiber cage in the cervical spine: a clinical and radiological study. Spine 27:1694–1701
40. Yang JY, Song HS, Lee M, Bohlman HH, Riew KD (2009) Adjacent level ossification development after anterior cervical fusion without plate fixation. Spine (Phila Pa 1976). 34(1):30-33
41. Wm Yue, Brodner W, Highland TR (2005) Persistent swallowing and voice problems after anterior cervical discectomy and fusion with allograft and plating: a 5 to 11 year follow-up study. Eur Spine J 14:677–682
