# Supplementary material for: Meta-Analysis Comparing Zero-Profile Spacer and Anterior Plate in Anterior Cervical Fusion
Source: PLoS One. 2015 Jun 11;10(6):e0130223. doi: 10.1371/journal.pone.0130223 (PMC4466022; doi:10.1371/journal.pone.0130223)
Supplement: S1 File — (ZIP) [file pone.0130223.s013.zip › S8_ZIP. Fve full-text excluded studies and reasons for exclusion/Excluded Study_2.pdf]

Stand-alone Cervical Cages Versus Anterior Cervical Plate in 2-Level Cervical Anterior Interbody Fusion Patients

Clinical Outcomes and Radiologic Changes

Jae Keun Oh, MD, Tae Yup Kim, MD,†‡ Hyo Sang Lee, MD,†‡ Nam Kyu You, MD,†‡ Gwi Hyun Choi, MD,†‡ Seong Yi, MD, PhD,†‡ Yoon Ha, MD, PhD,†‡ Keung Nyun Kim, MD, PhD,†‡ Do Heum Yoon, MD, PhD,†‡ and Hyun Chul Shin, MD, PhD§*

Study Design: Retrospective study.

Objectives: To compare the efficacy of 2-level anterior cervical discectomy and fusion with cage alone (ACDF-CA) and with cage and plate construct (ACDF-CPC) with regard to clinical outcome and radiologic changes.

Summary of Background Data: The use of stand-alone cervical interbody cages in ACDF has become popular, but high subsidence rates have been reported in the literature.

Methods: A total of 54 consecutive patients who underwent 2-level ACDF-CA or ACDF-CPC after suffering from cervical radiculopathy were divided into 2 groups: group A (n = 28) underwent ACDF-CA, group B (n = 26) underwent ACDF-CPC. Fusion rate, global and segmental kyphosis, disk height, and subsidence rate were assessed by radiolographs. Clinical outcomes were assessed using Robinson's criteria.

Results: Solid fusion was achieved in 96.43% (27/28) in group A and in 96.15% (25/26) in group B. Fusion segmental kyphosis of > 5 degrees occurred in 14.29% (4/28) of group A and in 7.69% (2/26) of group B; however, there was no statistical difference between the 2 groups ($P > 0.05$). Subsidence occurred in 35.71% (10/28) of group A as compared with 11.54% (3/26) of group B ($P < 0.05$). Clinical outcomes were similar in the 2 treatment groups.

Conclusions: The use of cage and plate construct in 2-level ACDF results in a shorter fusion duration and a lower subsidence rate than that of cage alone; however, there is no significant difference in the postoperative global and segmental alignment and clinical outcomes between groups.

Key Words: anterior cervical fusion, PEEK cage, plate construct, subsidence, fusion

(*J Spinal Disord Tech* 2013;26:415–420)

Anterior cervical discectomy and fusion (ACDF) has become a standard surgical procedure for treating degenerative disk disease associated with radiculopathy or myelopathy.[1,2] Many modifications of this technique have been reported since its original description by Smith and Robinson and the later report by Cloward.[1,3] Although ACDF for the treatment of degenerative cervical disease is a highly successful procedure, its success rates decline in multilevel discectomies as the number of levels increases.[4,5]

Relatively good results have been reported when performing ACDF with cage alone (ACDF-CA)[6]; however, controversy remains regarding the high incidence of complications such as cage subsidence, kyphotic deformity, and pseudoarthrosis.[7,8] Long-term clinical and radiologic outcomes of 2-level contiguous ACDF operations using polyetheretherketone (PEEK) cages have been examined in several previous studies.[9,10] We compared and analyzed the radiologic and clinical outcomes of ACDF-CA and ACDF with cage and plate construct (ACDF-CPC) for the surgical treatment of 2 contiguous-level degenerative disk disease of the cervical spine to evaluate the efficacy of metal plate augmentation.

MATERIALS AND METHODS

Materials

Patients who presented at our hospital between March 2007 and March 2009 with degenerative cervical radiculopathy resistant to conservative treatment with no history of prior cervical surgery were included. Patients with trauma, infection, or neoplasms were excluded. Indications for surgery included intractable radiculopathy and myelopathy, or a combination of the 2, due to nerve root or spinal cord compression and compatible magnetic

Received for publication May 30, 2011; accepted January 23, 2012.
From the *Department of Neurosurgery, Spine Center, Hallym University Sacred Heart Hospital; †Department of Neurosurgery, College of Medicine, Yonsei University; ‡Spine and Spinal Cord Research Institute, College of Medicine, Yonsei University; and §Department of Neurosurgery, Kangbuk Samsung Hospital, College of Medicine, Sungkyunkwan University, Seoul, Korea.
The authors declare no conflict of interest.
Reprints: Keung Nyun Kim, MD, PhD, Department of Neurosurgery, College of Medicine, Yonsei University, 250 Seonanno, Seodaemun-gu, Seoul 120-752, Korea (e-mail: knkim@yuhs.ac).
Copyright © 2012 by Lippincott Williams & Wilkins

TABLE 1. Patients' Demographic Data

	Group A	Group B
Cases	28	26
Mean age (range; y)	57.9 (43–72)	54.3 (31–71)
Male	13	20
Female	15	6
Mean F/U (range; mo)	23.4 (12–46)	20.6 (12–36)

resonance imaging findings. ACDF-CA was primarily performed during the first half of the study, and ACDF-CPC was primarily performed during the latter half. No specific guidelines or indications were used in dividing the patients into these 2 groups. Because there were some cage subsidence cases in ACDF-CA, we changed our practice from ACDF-CA to ACDF-CPC. Group A consists of 28 subjects who underwent ACDF-CA. Group B consists of 26 subjects who underwent ACDF-CPC. Regarding surgical levels, in group A, there were 2 patients at levels C3–C5, 15 at C4–C6, and 11 at C5–C7. In group B, there were 3 patients at levels C3–C5, 9 at C4–C6, and 14 at C5–C7. The mean follow-up period was 23.4 months (12–46 mo) for group A and 20.6 months (12–36 mo) for group B. The mean age was 57.9 years (43–72 y) in group A and 54.3 years (31–71 y) in group B. Group A consisted of 13 men and 15 women, and group B consisted of 20 men and 6 women (Table 1).

Surgical Methods

The surgical procedure was performed as described by Smith and Robinson.[3] After discectomy was performed, the posterior longitudinal ligament with posterior bony spur or uncovertebral joint was removed when the surgical microscope revealed cervical spondylosis or foraminal pathology. The endplate cartilage was also removed using a curette. The upper and lower endplates were prepared by removing the overlying cartilage, preserving the hardest subchondral bone. Vertebral bodies were distracted with a Caspar distractor and an optimal PEEK cage was selected (Solis cage, Stryker Spine). The cage was horseshoe shaped and contained a hollow cylinder that was filled with a bone graft. The cage also had retention teeth and bilateral titanium spikes on the superior and inferior surfaces. A PEEK cage packed with demineralised bone matrix was inserted into the disk space. In cases with plate augmentation, we used an anterior cervical plate (Zephir anterior cervical system, Medronic Sofamor Danek). All patients in both groups used a Philadelphia collar for 4 weeks.

Methods

Clinical and radiologic follow-up was performed immediately after surgery, and then 1, 3, 6, 12, and 24 months after surgery. Spinal alignment and fusion status were assessed with anteroposterior and lateral

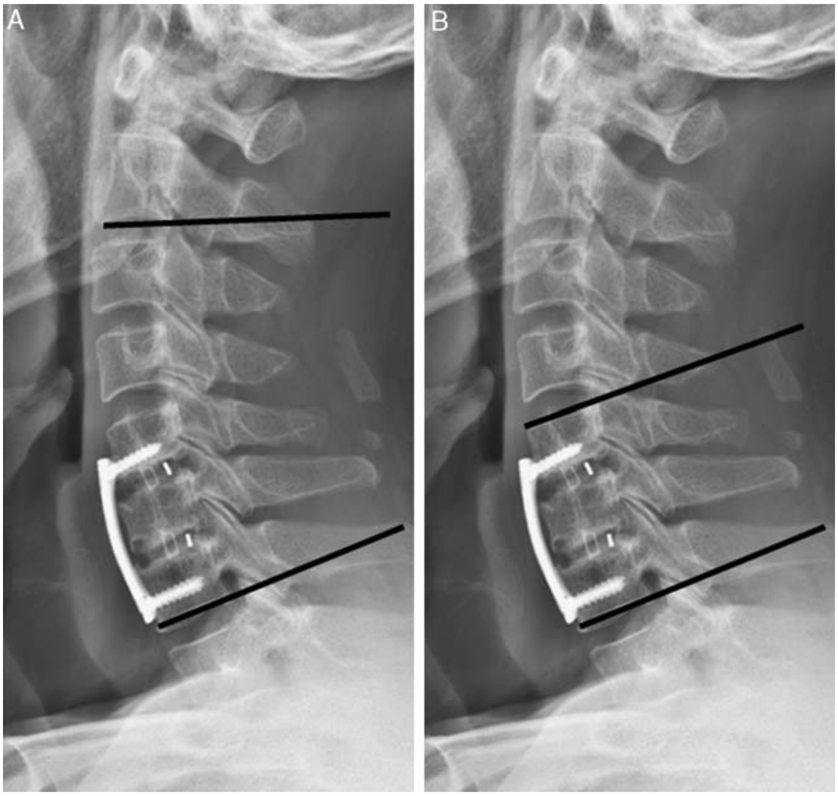

FIGURE 1. A, Global alignment was measured by Cobb's angle between the inferior endplate of the C2 vertebral body and the inferior endplate of the C7 vertebral body. B, Regional alignment was measured using Cobb's angle between the upper endplate of the most cranial vertebral body and the lower endplate of the most caudal vertebral body.

© 2012 Lippincott Williams & Wilkins

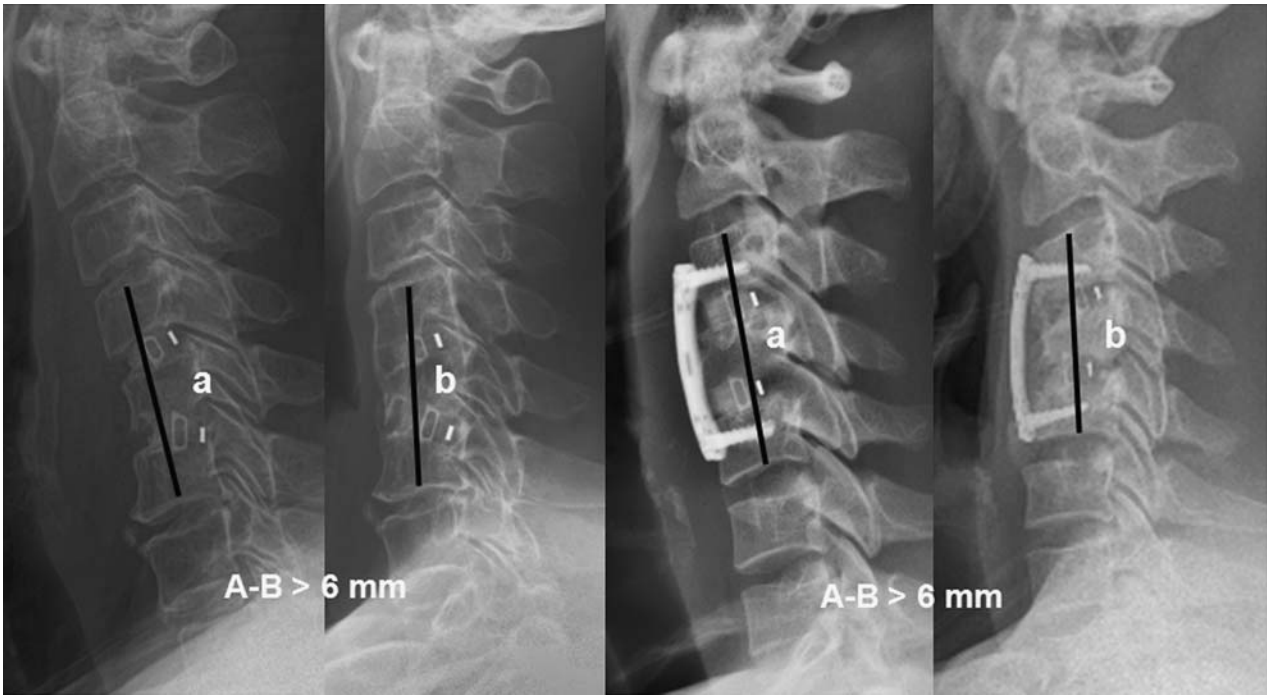

FIGURE 2. Cage subsidence was defined as >6 mm of distance between the midpoint of the upper margin of the upper vertebral body and the lower margin of the lower vertebral body.

(neutral, flexion, and extension) radiographs. An independent radiologist evaluated the radiographs without knowledge of the clinical outcome.

Fusion was defined as < 2-degree movement on lateral flexion/extension views, the presence of bridging trabecular bone between the endplates on anteroposterior/lateral views, the lack of implant failure signs of the anterior plate system, and < 50% radiolucency in the perimeter surrounding the cage.

Cervical lordosis was measured using Cobb's angle. Global alignment was measured by Cobb's angle between the inferior endplate of the C2 vertebral body and the inferior endplate of the C7 vertebral body. Regional alignment was measured using Cobb's angle between the upper endplate of the most cranial vertebral body and the lower endplate of the most caudal vertebral body (Fig. 1).

Cage subsidence was defined as > 6 mm distance between the midpoint of the upper margin of the upper vertebral body and the lower margin of the lower vertebral body (Fig. 2).

TABLE 2. Robinson's Criteria

Outcome	Pain	Medication	Activity	Work Status
Excellent	None	None	Normal	Normal
Good	Mild	Occasional NSAIDs	Normal	Normal
Fair	Moderate	Frequent NSAIDs	Restricted	Limited
Poor	Severe	Oral narcotics	Incapacitated	Disabled

NSAID indicates nonsteroidal anti-inflammatory drug.

Clinical outcomes were assessed using Robinson's criteria (Table 2).[11]

RESULTS

Patients Population

In group A, there were 13 men and 15 women. In group B, there were 20 male patients and 6 female patients. There was a statistically significant difference between groups A and B with regard to sex (Table 1, Fisher exact test, $P = 0.022$). However, it was not different statistically with regard to age (Mann-Whitney test, $P = 0.308$) and surgery level (Fisher exact test, $P = 0.413$).

Radiologic Results

At the final follow-up, the fusion rates were 96.43% (27/28) in group A and 96.15% (25/26) in group B. However, the mean fusion time was 7.39 ± 2.67 in group A and 4.54 ± 2.86 in group B. This difference was statistically significant (Table 3, Mann-Whitney test, $P < 0.001$).

In group A, the global lordotic angle was 11.99 ± 12.00 before the operation, 7.80 ± 10.95 immediately after the operation, and 8.74 ± 11.19 at the final follow-up. The lordotic angle of the fusion segment in group A

TABLE 3. Fusion Rate and Mean Fusion Time

Group	A	B	*P*
Fusion rate	27/28 (96.43%)	25/26 (96.15%)	1.000
Mean fusion time (mo)	7.39 ± 2.67	4.54 ± 2.86	< 0.001

© 2012 Lippincott Williams & Wilkins

TABLE 4. Mean Lordotic Angle of the Global and Fusion Segments

	A		B	
Group	Global	Segment	Global	Segment
Preop*	11.99 ± 12.00	1.59 ± 8.81	14.44 ± 8.29	1.98 ± 8.90
Postop immediately†	7.80 ± 10.95	2.04 ± 8.87	10.53 ± 6.50	4.19 ± 6.29
Postop final	8.74 ± 11.19	−0.09 ± 8.26	9.61 ± 8.91	1.50 ± 6.66
Kyphosis > 5 degrees	3/28 (10.71%)	4/28 (14.29%)	5/26 (19.23%)	2/26 (7.69%)

*Preoperative mean lordotic angle (degrees).
†Postoperative mean lordotic angle (degrees).

was 1.59 ± 8.81 before the operation, 2.04 ± 8.87 immediately after the operation, and −0.09 ± 8.26 at the final follow-up. In group B, the global lordotic angle was 14.44 ± 8.29 before the operation, 10.53 ± 6.50 immediately after the operation, and 9.61 ± 8.91 at the final follow-up. The lordotic angle of the fusion segment in group B was 1.98 ± 8.90 before the operation, 4.19 ± 6.29 immediately after the operation, and 1.50 ± 6.66 at the final follow-up. However, there are not enough valid cases to perform a statistical analysis to explain why the lordotic angle was changed (Table 4).

The degree of change in disk height of the fusion segments between the final disk height and the measurements taken immediately after the operation were 4.34 ± 2.49 in group A and 4.16 ± 2.24 in group B. Cage subsidence was observed in 10 cases in group A and in 3 cases in group B. There was a statistically significant difference not in disk height change but in cage subsidence between groups A and B (Table 5, Mann-Whitney test, $P = 0.687$, $P = 0.038$).

Clinical Outcomes

According to the method described by Riley et al,[11] 82.14% (23/28) of the cases in group A and 96.15% (25/26) in group B yielded outcomes with a grade higher than "good" (Table 6, Fisher exact test, $P = 0.2670$).

Complication

There was only 1 case with a plate-related complication such as screw back-out or plate bending (Fig. 3). However, revision surgery was not indicated because the patient was asymptomatic. This case showed evidence of fusion in subsequent visits.

TABLE 5. Change in Disk Height and Subsidence

	A	B	P
Postop disk height*	54.15 ± 4.30	57.02 ± 3.98	—
Final disk height†	49.81 ± 4.20	52.95 ± 3.45	—
Disk height change	4.34 ± 2.49	4.16 ± 2.24	0.687
Cage subsidence‡	10/28 (35.71%)	3/26 (11.54%)	0.038

*Immediate postoperative fusion segment height (mm).
†Final fusion segment height (mm).
‡Number.

TABLE 6. Robinson's Clinical Outcome Assessment

	A	B	P
Excellent	15/28 (53.57%)	21/26 (80.77%)	—
Good	8/28 (28.57%)	4/26 (15.38%)	—
Fair	4/28 (14.29%)	1/26 (3.85%)	—
Poor	1/28 (3.57%)	0/26 (0%)	—
> Good	23/28 (82.14%)	25/26 (96.15%)	0.2670

In group A, there was 1 revision surgery because of disease in the adjacent level. An upper-level arthroplasty was performed 36 months after the first operation.

DISCUSSION

ACDF has been widely used as a surgical treatment for cervical spinal disorders, including spondylosis, myelopathy, herniated disks, trauma, and degenerative disk disease. The consensus holds that the success of this procedure relies on thorough decompression and development of solid osseous fusion. According to the literature, the use of autologous bone grafts, allografts, bone substitutes, internal fixation, or any graft remains controversial.[12–14]

We compared and analyzed the radiologic and clinical outcomes of ACDF-CA and ACDF-CPC for the surgical treatment of 2 contiguous-level degenerative disk diseases of the cervical spine. In our study, no specific guidelines or indications were used to divide the patients among the 2 groups.

Some authors have reported that ACDF-CA has the advantages of shorter operation time, minimal blood loss, and relative simplicity as compared with ACDF-CPC. The plate complication rate varies from 2.2% to 24.0%, including screw pullout; screw breakage; injury to the laryngeal nerve, esophagus, spinal cord, or root; vertebral artery injury; and wound infection.[4,15] In our study, there was 1 plate-related complication among the 26 cases (3.8%). In that case, revision surgery was not indicated because the patient was asymptomatic. This case showed evidence of fusion in subsequent visits.

Some articles have shown that the use of a cage and plate construct in ACDF results in more lordotic alignment, increased disk height, higher fusion rates, lower subsidence rates, and lower complication rates than that of cage alone; however, there is no significant difference in clinical outcomes between groups.[16] The results of our study showed that ACDF-CPC had a shorter fusion time and less subsidence than ACDF-CA. However, there were no significant differences in the global and segment alignment and in the clinical outcomes between the groups.

Initial stability is also important for reducing cage subsidence. In an ex vivo study using human cadaveric spine segments, the biomechanical results for the 3 cages and bone cement were compared.[17] The 3 cages acted as an alternative to bone cement in providing the primary stabilizing effect of cervical interbody fusion. In that study, the initial stability of stand-alone interbody cages was compared with that of the cortical bone or the plate system. In flexion and extension, the plate had a

© 2012 Lippincott Williams & Wilkins

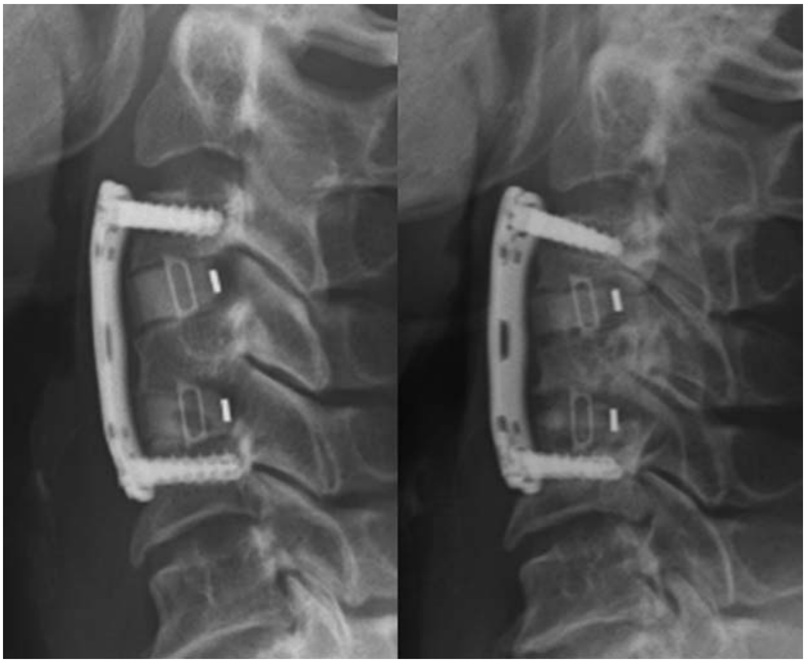

FIGURE 3. A case with a plate-related complication such as screw back-out or plate bending.

significantly smaller range of motion than the cage and the autograft, and the cage had a significantly greater range of motion than the intact spine. The results of another biomechanical study suggested that the cervical interbody cage should be supplemented with additional external or internal support to prevent excessive motion in flexion and extension.[18]

Maintenance of cervical alignment is an important factor, particularly in multilevel disease, because misalignment after spinal fusion promotes degenerative changes in the intervertebral levels adjacent to the fused segment.[19–22] Katsuura et al[21] reported that local kyphosis at the fused segment was observed in only 13% of patients with single-level intervertebral fusion but in 53% of patients with multiple-level fusion. They also noted that the use of an anterior plate was effective in maintaining local lordosis in patients with anterior multiple-level fusion for degenerative disorders.[20] Wang et al[23] reported significantly less disk space collapse and kyphotic deformity with plated fusion than with nonplated fusion. Fujibayashi and colleagues showed that 44% of the patients treated with a stand-alone cage exhibited a loss of lordotic alignment of >5 degrees. However, other groups have reported preservation of preoperative lordosis at the final follow-up in patients who underwent multilevel ACDF-CA.[4,24,25] In our study, however, there was no significant difference in the postoperative global and segmental alignment between the 2 groups. In both groups, the final postoperative kyphosis was higher than it was immediately after the operation. We believe that the reason for the increasing kyphosis is the natural aging process.

Many studies have corroborated the trend toward earlier development of adjacent segment disease with instrumentation after lumbar or lumbosacral fusion. The immediate rigidity produced by instrumentation causes greater stress, leading to accelerated degeneration in the neighboring levels.[26,27] In cervical fusion, we thought the same situation was occurring after ACDF. Thus, in the future, it will be necessary to study adjacent segment disease between ACDF-CA and ACDF-CPC.

The short follow-up period of this study does not allow for a thorough evaluation of the development of adjacent-level degeneration. Therefore, long-term follow-up studies are necessary, which we plan to pursue in the future. Another limitation of this study is that it is retrospective. The sex distribution was different between groups A and B. There was a lack of medical records of immediate postoperative complication such as dysphagia and hoarseness. However, there was no case of persisting dysphagia or hoarseness after operation. Thus, a prospective randomized study will be required in the future.

CONCLUSIONS

The use of a cage and plate construct in 2-level ACDF results in a shorter fusion duration and a lower subsidence rate than cage alone; however, there is no significant difference in the postoperative global and segmental alignment and in the clinical outcomes between the groups.

REFERENCES

1. Cloward RB. The anterior approach for removal of ruptured cervical disks. *J Neurosurg*. 1958;15:602–617.
2. Gore DR, Sepic SB. Anterior cervical fusion for degenerated or protruded discs. A review of one hundred forty-six patients. *Spine (Phila Pa 1976)*. 1984;9:667–671.

© 2012 Lippincott Williams & Wilkins

3. Smith GW, Robinson RA. The treatment of certain cervical-spine disorders by anterior removal of the intervertebral disc and interbody fusion. *J Bone Joint Surg Am*. 1958;40-A:607–624.
4. Demircan MN, Kutlay AM, Colak A, et al. Multilevel cervical fusion without plates, screws or autogenous iliac crest bone graft. *J Clin Neurosci*. 2007;14:723–728.
5. Heidecke V, Rainov NG, Marx T, et al. Outcome in Cloward anterior fusion for degenerative cervical spinal disease. *Acta Neurochir (Wien)*. 2000;142:283–291.
6. Vavruch L, Hedlund R, Javid D, et al. A prospective randomized comparison between the cloward procedure and a carbon fiber cage in the cervical spine: a clinical and radiologic study. *Spine (Phila Pa 1976)*. 2002;27:1694–1701.
7. Hacker RJ. Threaded cages for degenerative cervical disease. *Clin Orthop Relat Res*. 2002;39–46.
8. Majd ME, Vadhva M, Holt RT. Anterior cervical reconstruction using titanium cages with anterior plating. *Spine (Phila Pa 1976)*. 1999;24:1604–1610.
9. Barsa P, Suchomel P. Factors affecting sagittal malalignment due to cage subsidence in standalone cage assisted anterior cervical fusion. *Eur Spine J*. 2007;16:1395–1400.
10. Kulkarni AG, Hee HT, Wong HK. Solis cage (PEEK) for anterior cervical fusion: preliminary radiological results with emphasis on fusion and subsidence. *Spine J*. 2007;7:205–209.
11. Riley LH Jr, Robinson RA, Johnson KA, et al. The results of anterior interbody fusion of the cervical spine. Review of ninety-three consecutive cases. *J Neurosurg*. 1969;30:127–133.
12. Walker AE, Robinson RA. Anterior cervical fusion. *Dia Med*. 1962;34(special):894–900.
13. Savolainen S, Rinne J, Hernesniemi J. A prospective randomized study of anterior single-level cervical disc operations with long-term follow-up: surgical fusion is unnecessary. *Neurosurgery*. 1998;43:51–55.
14. Thalgott JS, Fritts K, Giuffre JM, et al. Anterior interbody fusion of the cervical spine with coralline hydroxyapatite. *Spine (Phila Pa 1976)*. 1999;24:1295–1299.
15. Kaiser MG, Haid RW Jr, Subach BR, et al. Anterior cervical plating enhances arthrodesis after discectomy and fusion with cortical allograft. *Neurosurgery*. 2002;50:229–236; discussion 236–228.
16. Song KJ, Taghavi CE, Lee KB, et al. The efficacy of plate construct augmentation versus cage alone in anterior cervical fusion. *Spine (Phila Pa 1976)*. 2009;34:2886–2892.
17. Wilke HJ, Kettler A, Claes L. Primary stabilizing effect of interbody fusion devices for the cervical spine: an in vitro comparison between three different cage types and bone cement. *Eur Spine J*. 2000;9:410–416.
18. Shimamoto N, Cunningham BW, Dmitriev AE, et al. Biomechanical evaluation of stand-alone interbody fusion cages in the cervical spine. *Spine (Phila Pa 1976)*. 2001;26:E432–E436.
19. Jackson RP, McManus AC. Radiographic analysis of sagittal plane alignment and balance in standing volunteers and patients with low back pain matched for age, sex, and size. A prospective controlled clinical study. *Spine (Phila Pa 1976)*. 1994;19:1611–1618.
20. Katsuura A, Hukuda S, Imanaka T, et al. Anterior cervical plate used in degenerative disease can maintain cervical lordosis. *J Spinal Disord*. 1996;9:470–476.
21. Katsuura A, Hukuda S, Saruhashi Y, et al. Kyphotic malalignment after anterior cervical fusion is one of the factors promoting the degenerative process in adjacent intervertebral levels. *Eur Spine J*. 2001;10:320–324.
22. Oda I, Cunningham BW, Buckley RA, et al. Does spinal kyphotic deformity influence the biomechanical characteristics of the adjacent motion segments? An in vivo animal model. *Spine (Phila Pa 1976)*. 1999;24:2139–2146.
23. Wang JC, McDonough PW, Endow KK, et al. Increased fusion rates with cervical plating for two-level anterior cervical discectomy and fusion. *Spine (Phila Pa 1976)*. 2000;25:41–45.
24. Cho DY, Lee WY, Sheu PC. Treatment of multilevel cervical fusion with cages. *Surg Neurol*. 2004;62:378–385; discussion 385–376.
25. Topuz K, Colak A, Kaya S, et al. Two-level contiguous cervical disc disease treated with peek cages packed with demineralized bone matrix: results of 3-year follow-up. *Eur Spine J*. 2009;18:238–243.
26. Etebar S, Cahill DW. Risk factors for adjacent-segment failure following lumbar fixation with rigid instrumentation for degenerative instability. *J Neurosurg*. 1999;90:163–169.
27. Kumar MN, Baklanov A, Chopin D. Correlation between sagittal plane changes and adjacent segment degeneration following lumbar spine fusion. *Eur Spine J*. 2001;10:314–319.

 © 2012 Lippincott Williams & Wilkins
