# Supplementary material for: Meta-Analysis Comparing Zero-Profile Spacer and Anterior Plate in Anterior Cervical Fusion
Source: PLoS One. 2015 Jun 11;10(6):e0130223. doi: 10.1371/journal.pone.0130223 (PMC4466022; doi:10.1371/journal.pone.0130223)
Supplement: S1 File — (ZIP) [file pone.0130223.s013.zip › S8_ZIP. Fve full-text excluded studies and reasons for exclusion/Excluded Study_3.pdf]

SPINE Volume 34, Number 2, pp 156–160
©2009, Lippincott Williams & Wilkins

A New Stand-Alone Cervical Anterior Interbody Fusion Device

Biomechanical Comparison With Established Anterior Cervical Fixation Devices

Matti Scholz, MD,* Phillip M. Reyes, BSE,† Philipp Schleicher, MD,* Anna G. U. Sawa, MS,† Seungwon Baek, MS,† Frank Kandziora, MD,* Frederick F. Marciano, MD, PhD,‡ and Neil R. Crawford, PhD†

Study Design. A new anchored spacer—a low-profile cervical interbody fusion cage with integrated anterior fixation—was compared biomechanically to established anterior cervical devices.

Objective. To evaluate the fixation properties of the new stand-alone device and compare these properties with established fixation methods. The hypothesis is that the new device will provide stability comparable to that provided by an anterior cervical cage when supplemented with an anterior plate.

Summary of Background Data. It is accepted that the use of anterior cervical plating increases the chance of achieving a solid fusion. However, its use may be associated with an increase in operation time and a higher postoperative morbidity caused by a larger anterior approach and disruption of the anterior musculature. This dilemma has led to the development of a new, low profile stand-alone cervical anterior cage device with integrated screw fixation.

Methods. Twenty-four human cadaveric C4–C7 cervical spines were loaded nondestructively with pure moments in a nonconstraining testing apparatus to induce flexion, extension, lateral bending, and axial rotation while angular motion was measured optoelectronically. The specimens were tested:

1. Intact (N = 24).
2. After discectomy and anterior stabilization.
a. Interbody cage + locking plate (N = 8).
b. Interbody cage + dynamic plate (N = 8).
c. Anchored spacer (N = 8).
3. After ventral plate removal of group 2a and 2b (N = 16).

Results. All fixation techniques decreased range of motion (ROM) and lax zone (LZ) ($P < 0.05$) in all test modes compared with the intact motion segment and cage-only group. There were no significant differences between the anchored spacer and cage + locking plate or cage + dynamic plate.

Conclusion. The anchored spacer provided a similar biomechanical stability to that of the established anterior fusion technique using an anterior plate plus cage and has a potentially lower perioperative and postoperative morbidity. These results support progression to clinical trials using the cervical anchored spacer as a stand-alone implant.

Key words: anterior cervical fusion, stand alone, cervical fixation, biomechanical evaluation. **Spine 2009;34: 156–160**

Degenerative conditions of the cervical spine, such as degenerative disc disease, cervical spondylotic myelopathy, and cervical disc prolapse are commonly treated by discectomy and subsequent interbody fusion. One common fixation method is the implantation of an interbody cage to restore disc and foraminal height and to stabilize the segment until bony fusion has occurred. The interbody cage provides stability only through tensioning of the remaining ligaments. Thus, it offers little stabilization during extension because the anterior ligamentous structures are absent after discectomy. Some surgeons therefore prefer to add an anterior plate to enhance stabilizing properties.[1]

Although the profile of current anterior plates is lower than that of earlier designs, they are still somewhat bulky and might lead to postoperative complications.[2] Furthermore, the application of these plates is a time-consuming surgical procedure, during which the vital structures on the anterior aspect of the cervical spine, such as the trachea, carotid arteries, and esophagus are endangered.[3]

Based on the SynFix-LR,[4] which is designed for anterior stand-alone stabilization of the lumbar spine motion segment, a similar but smaller implant for the cervical spine has been developed. The Zero-P, from this point referred to as the "anchored spacer," has a lower profile than a standard cage with an additional anterior plate. Because the design of the cervical implant and the biomechanics of the cervical spine are somewhat different from the lumbar implant and the lumbar spine, the biomechanical properties of this construct remain unclear.

This study compared the stabilizing effects of the anchored spacer after anterior cervical discectomy in human cadaveric spine specimens to the stabilizing effects

From the *Center for Spinesurgery and Neurotraumatology, Trauma Clinic Frankfurt/Main, Germany; †Spinal Biomechanics Research Laboratory, and ‡Division of Neurological Surgery, Barrow Neurological Institute, Phoenix, AZ.
Acknowledgment date: May 14, 2008. First revision date: August 20, 2008. Acceptance date: 25, 2008.
The device(s)/drug(s) FDA-approved or approved by corresponding national agency for this indication.
Corporate/Industry funds were received in support of this work. One or more author(s) has/have received benefits for personal or professional use from a commercial party related directly or indirectly to the subject of this manuscript: *e.g.*, honoraria, gifts, consultancies.
Address correspondence and reprint requests to Matti Scholz, MD, Center for Spinesurgery and Neurotraumatology, Trauma Clinic Frankfurt/Main, Friedberger Landstraße 430, 60389 Frankfurt/Main, Germany; E-mail: matti.scholz@bgu-frankfurt.de

Copyright © Lippincott Williams & Wilkins. Unauthorized reproduction of this article is prohibited.

of 2 other common surgical constructs: (1) cage plus anterior rigid screw plate, and (2) cage plus anterior semiconstrained (translating) screw-plate. In addition, the specimens were retested with the plate removed as a stand-alone cage construct. We hypothesized that the anchored spacer would provide significantly better stability than a stand-alone cage and would provide stability approaching that of the cage/plate constructs.

■ Materials and Methods

Specimen Preparation

Twenty-four fresh-frozen human cadaveric spines (C4–C7) were studied using standard flexibility testing methods. Specimens were harvested from 9 men and 15 women donors (mean age, 61 years; range, 31–82 years). The medical history of each donor was reviewed to exclude trauma, malignancy, or metabolic disease that might otherwise compromise the mechanical properties of the cervical spine. Plain film radiographs were taken and specimens with any obvious radiographic or visible flaws (especially osteophytes, disc narrowing, or joint arthrosis) were excluded and replaced. Anteroposterior dual energy radiograph absorptiometry scans were performed at C6 on each specimen to assess bone mineral density (BMD). Specimens with scores indicating obvious osteoporosis were excluded and replaced. Specimens were separated into 3 groups of 8, and the equivalence of the groups was verified by comparing mean BMD values and ages.

All specimens were thawed using a bath of 0.9% saline solution at 30°C. Dissection was performed carefully so as to preserve all ligaments, joint capsules, discs, and osseous structures. The C4 vertebral body was potted using household wood screws and polymethylmethacrylate (PMMA) in a metal fixture. Similarly, the C7 vertebral body was potted in a metal fixture and attached to the base of the testing apparatus. To prevent dehydration, specimens were kept wrapped loosely in saline-soaked gauze during testing.

Implants

Specimens were tested in 3 conditions: (1) intact (N = 24), (2) after discectomy and group specific anterior stabilization (N = 8): (2a) interbody cage plus locking plate, (2b) interbody cage plus dynamic plate, (2c) stand-alone anchored spacer, and (3) after removal of locking plate or dynamic plate (N = 16).

The locking plate used was a rigid plating system (CSLP, Synthes), meaning that the screws do not move relative to the plate. The dynamic plate used was a translating plating system (Vectra-T, Synthes) that allows for postoperative subsidence. The translation is enabled through carriages that slide on the plate base. The test device was an anchored spacer (Zero-P, Synthes), which combines an interbody spacer with a rigid screw fixation mechanism that is contained within the excised disc space. The interbody cage used was a poly-ether-ether-ketone (PEEK) interbody spacer (Vertebral Spacer-CR, Synthes).

Surgical Procedures

After intact flexibility testing, specimens were positioned supine, gently clamped in a vice, and decompressed with a single-level discectomy of C5–C6. For discectomy, the disc material was removed using rongeurs and a curette after incision of the ventral annulus. The posterior longitudinal ligament was preserved in each specimen.

After discectomy, specimens in each group received hardware of an appropriate size as determined from individual anatomy. During plate insertion (groups 2a and 2b) or anchored spacer insertion (group 2c), lateral fluoroscopy was used to anatomically assess the size of the specimen so that screw length could be chosen to span approximately two-thirds of the anteroposterior vertebral body depth (Figure 1). After completing flexibility tests, the plates were removed from the specimens in groups 2a and 2b and the cage-only condition (N = 16) was tested, providing a negative control.

Biomechanical Testing

For flexibility tests, a nonconstraining, nondestructive pure moment was applied to each specimen through a system of cables and pulleys in conjunction with a standard servohydrau-

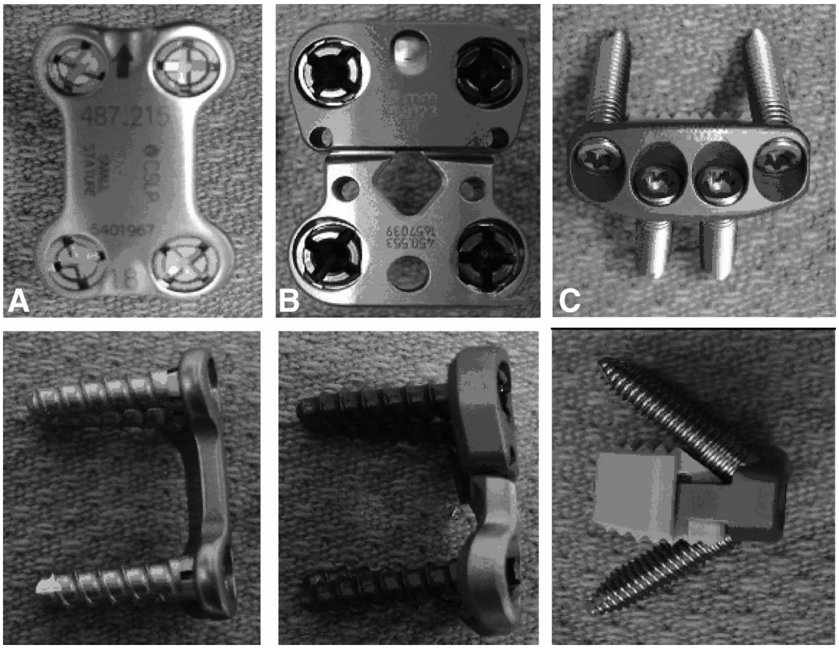

Figure 1. Devices studied. **A**, Locking titanium plate, which uses set screws within anchoring screws to create a rigid screw-plate interface; **B**, Dynamic titanium plate, with translation achieved by sliding interface between the two plate halves; and **C**, Anchored spacer, consisting of a poly-ether-ether-ketone spacer rigidly attached to a titanium anchoring piece through which anchoring screws are placed diagonally.

Copyright © Lippincott Williams & Wilkins. Unauthorized reproduction of this article is prohibited.

Table 1. Mean ROM and LZ (Degrees) ± Standard Deviation

Loading Mode and Parameter	Intact n = 24	Cage + Locking Plate n = 8	Cage + Dynamic Plate n = 8	Anchored Spacer n = 8	Cage-Only n = 16
Flexion					
ROM	6.5 ± 1.5	1.8 ± 0.8	1.8 ± 1.1	2.6 ± 1.5	5.1 ± 1.7
LZ	4.3 ± 1.1	0.9 ± 1.4	1.0 ± 1.0	1.7 ± 1.7	5.2 ± 2.7
Extension					
ROM	6.7 ± 1.5	1.4 ± 1.3	2.1 ± 1.3	2.4 ± 1.4	3.7 ± 1.7
LZ	4.3 ± 1.1	0.9 ± 1.4	1.0 ± 1.0	1.7 ± 1.7	5.2 ± 2.7
Bending					
ROM	4.6 ± 1.4	1.1 ± 0.5	1.3 ± 1.0	1.2 ± 0.5	2.7 ± 1.2
LZ	3.5 ± 1.4	0.3 ± 0.2	0.5 ± 0.8	0.3 ± 0.2	2.2 ± 1.9
Rotation					
ROM	4.5 ± 1.2	1.5 ± 0.7	1.6 ± 0.9	1.5 ± 0.5	3.5 ± 1.3
LZ	3.3 ± 1.0	0.8 ± 0.8	0.7 ± 0.9	0.6 ± 0.3	3.4 ± 2.4

lic test system (MTS, Minneapolis, MN), as described previously.[5] Loads were applied about the appropriate anatomic axes to induce flexion, extension, left and right lateral bending, and left and right axial rotation. In each loading mode, 3 preconditioning cycles of 1.5 Nm held for 60 seconds were applied before data collection. Then, after resting at 0 load for 60 seconds, loads were applied in 0.25-Nm increments (each increment held for 45 seconds) to a maximum of 1.5 Nm. Three-dimensional specimen motion in response to the loads was determined using the Optotrak 3020 system (Northern Digital, Waterloo, Ontario, Canada). This system measures the three-dimensional displacement of the infrared-emitting markers rigidly attached in a noncollinear arrangement to each vertebra. Custom software converted the marker coordinates to angles about each of the anatomic axes.[6,7] From the raw flexibility data, the angular range of motion (ROM) and lax zone (LZ, zone of ligamentous/hardware laxity) were calculated.[8]

Statistical Analysis

The data were analyzed in each loading mode using 1-way analysis of variance to determine whether angular ROM or LZ differed among locking plate, dynamic plate, and anchored spacer groups. Within each group, ROM and LZ in each instrumented condition were compared to normal ROM and LZ using a paired 2-tailed Student *t* test. Locking plate plus cage and dynamic plate plus cage were compared to cage only, using a paired 1-tailed Student *t* test; anchored spacer was compared to cage only using a nonpaired 1-tailed *t* test. *P* values less than 0.05 were considered significant.

Results

The mean spine BMD and ages were 0.54 g/cm^2 and 60.9 years for the cage plus locking plate group, 0.559 g/cm^2 and 61 years for the cage plus dynamic plate group, and 0.549 g/cm^2 and 60.8 years for the anchored spacer group. These values were not significantly different from each other (mean BMD, $P = 0.9$; mean age, $P = 0.9$). After testing, no bone fractures were found in any of the specimens, and none of the screws or plates showed signs of fracture, loosening, or breakage. In the intact test condition, no significant difference was found among test groups in any direction of loading in mean ROM ($P > 0.32$) or mean LZ ($P > 0.42$) (Tables 1–3, Figure 3).

Comparison Between Intact Motion Segment and Stabilization Techniques

All fixation techniques significantly ($P < 0.05$) decreased ROM and LZ in comparison to the intact motion segment in all test modes (Tables 2, 3, Figure 3).

Comparison Between Anchored Spacer and Plated Constructs (Locking Plate and Dynamic Plate)

During both flexion and extension, there was a slightly higher ROM in the anchored spacer group in comparison to the dynamic plate and rigid plate groups (Table 1, Figure 3). However, these differences were not statisti-

Table 2. *P* for Comparisons of Mean Range of Motion Among Groups/Conditions

Comparison	Flexion	Extension	Lateral Bending	Axial Rotation
Intact *vs.*				
Cage + locking plate	<0.001*	<0.001*	<0.001*	0.001*
Cage + dynamic plate	<0.001*	<0.001*	<0.001*	0.001*
Anchored spacer	<0.001*	<0.001*	<0.001*	<0.001*
Anchored spacer *vs.*				
Cage + dynamic plate	0.342	0.325	0.917	0.905
Cage + locking plate	0.342	0.325	0.917	0.905
Cage-only *vs.*				
Cage + locking plate	0.001*	0.012*	0.001*	0.001*
Cage + dynamic plate	<0.001*	0.018*	<0.001*	<0.001*
Anchored spacer	0.001*	0.035*	0.001*	<0.001*

*Statistically significant value ($P < 0.05$).

Table 3. *P* for Comparisons of Mean Lax Zone Among Groups/Conditions

Comparison	Flexion/Extension	Lateral Bending	Axial Rotation
Intact *vs.*			
Cage + locking plate	<0.001*	<0.001*	<0.001*
Cage + dynamic plate	<0.001*	<0.001*	<0.001*
Anchored spacer	<0.001*	0.001*	<0.001*
Anchored spacer *vs.*			
Cage + locking plate	0.421	0.598	0.825
Cage + dynamic plate	0.421	0.598	0.825
Cage-only *vs.*			
Cage + locking plate	0.003*	0.011*	0.003*
Cage + dynamic plate	<0.001*	0.009*	<0.001*
Anchored spacer	0.002*	0.005*	0.001*

*Statistically significant value ($P < 0.05$).

Copyright © Lippincott Williams & Wilkins. Unauthorized reproduction of this article is prohibited.

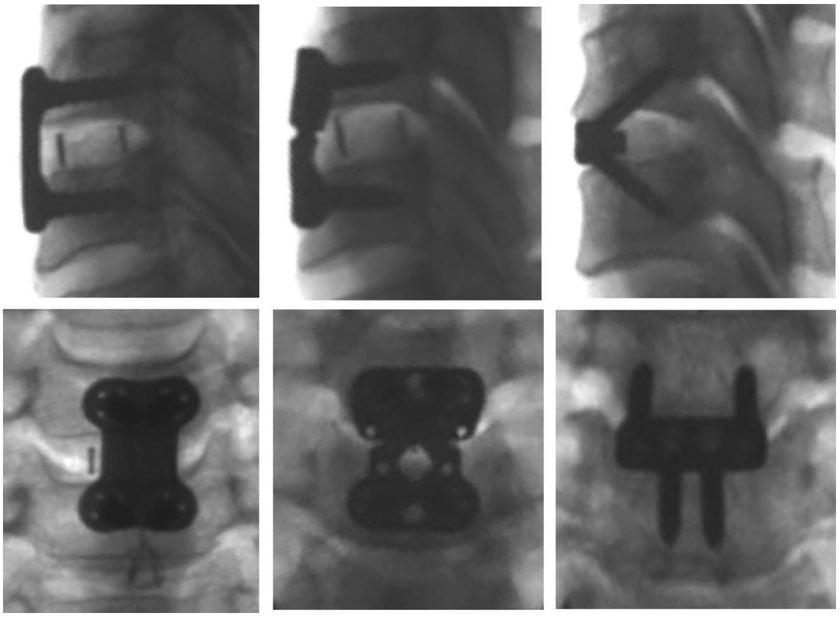

Figure 2. Plain x-rays of representative specimens from each test group demonstrating the prevertebral profile of the implants. **A**, Cage+Locking Plate; **B**, Cage+Dynamic Plate; and **C**, Anchored Spacer.

cally significant (Table 2). Additionally, there were no significant differences in ROM during lateral bending or axial rotation, or differences in LZ during any loading mode between the anchored spacer and plated constructs (Tables 2, 3).

Comparison Between Cage-Only and Stabilization Techniques

In comparison to the cage-only condition, all stabilization techniques decreased ROM and LZ (Tables 2, 3, Figure 3). These decreases were all statistically significant in all cases.

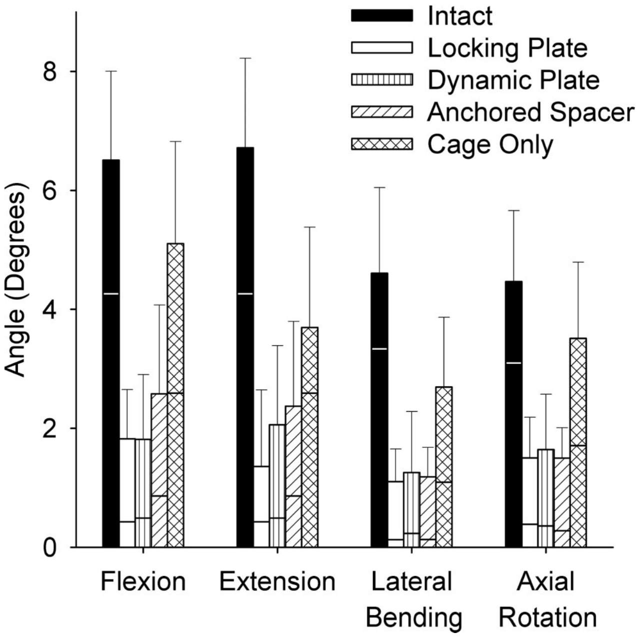

Figure 3. Graph showing range of motion (full bars) and lax zone (below horizontal dividing line) at C5–C6 during each loading mode and in each instrumented condition studied. Error bars show standard deviation of the range of motion.

■ Discussion

The current study was performed predominantly to evaluate the stability provided by a new cervical anterior interbody cage device incorporating integrated anterior fixation, and to compare the biomechanical characteristics of this implant to established cervical anterior fixation techniques.

The biomechanical advantages of restoring disc height and the integrity of the anterior column of the spine for treating discogenic pathology has been apparent for some time. In 1967, Bohler and Gaudernak[9] were among the first to describe anterior plating for the treatment of an acute cervical spinal fracture. Since that time, numerous reports have documented the effective use of anterior fixation in the treatment of spinal trauma. Early devices required penetration of the posterior cortex of the vertebral body (bicortical purchase) and thus there was a possibility of dural penetration and subsequent neurologic catastrophe. This drawback delayed the acceptance of these devices worldwide, particularly in the United States. The most popular, second generation systems (*e.g.*, CSLP from Synthes, Orion from Sofamor-Danek, Codman plate) featured screws fixed to the implant (angle stable screws) and permitted screw convergence on placement. The latest, third generation systems are dynamic semiconstrained plates that prevent stress shielding and allow subsidence. The use of all generations of these implants is associated with various intraoperative and postoperative complications. In their review of the literature, Coe and Vaccaro reported that the prevalence of screw and plate loosening was between 0% and 15.4%, of screw fracture between 0% and 13.3%, of plate fracture between 0% and 6.7%, of plate and graft displacement (with or without graft fracture) between 0% and 21.4%, and of implant malposition (screws in discs, plating of unfused segments, *etc.*) be-

Copyright © Lippincott Williams & Wilkins. Unauthorized reproduction of this article is prohibited.

tween 0% and 12.5%.[10] Newer implants are designed with a low profile to minimize the risk of dysphagia and esophageal perforation. Most modern plates also lock the screws to minimize implant loosening.

In their review of 109 patients with 3 different types of anterior cervical plates (Orozco, CSLP, and Orion), Lowery and McDonough found an overall failure rate of 35%, but combined failure for the locked systems (Orion and CSLP) of only 18%.[11] To avoid these implant related complications, a test device with a "zero profile" was developed. The profile and dimensions of this radiolucent anterior cervical interbody fusion device were based on the Syncage-C (Synthes GmbH, Switzerland) that has already demonstrated adequate biomechanical properties in previous studies.[12,13] As with the Synfix-LR,[4] the body of the anchored spacer is constructed of PEEK but incorporates a mechanically independent but integrated titanium plate into its anterior surface (Figure 3C).

In effect, integration of fixed angle anterior fixation screws directed toward the stronger lateral margin of the adjacent vertebra reconstructs the anterior tension band otherwise lost through resection of the anterior longitudinal ligament. The divergent orientation of these screws in combination with the locking mechanism of the screws into the plate was expected to provide additional stability in all planes.

Our results show that there are no statistical differences in the stability offered by this new stand-alone device as compared to that offered by any of the cage plus anterior plate constructs. The results of this study indicate that the stability of this new implant is, at the very least, equivalent to that provided by an anterior cage stabilized by additional anterior plating.

Study Limitations

In this *in vitro* investigation, isolated cadaveric spines with removed muscular tissue were studied. *In vivo*, muscle forces and the complex integration of neural feedback provide some additional control of segmental spinal motion and similarly contribute to the load that must be withstood by any instrumentation construct.[14,15] Therefore, this study did not include forces representing muscular interaction. When these findings are applied to the clinical scenario, the actual stability *in vivo* would likely be better than the one documented here.

The level of stiffness required to obtain long-term stability and fusion by this fixation method remains unanswered in the present study. However, the authors believe that there is sufficient supportive evidence to begin clinical trials and to evaluate the efficacy of this implant in prospective studies.

■ Conclusion

The anchored cervical interbody spacer provided similar biomechanical stability to the established anterior fusion technique (cage plus additional ventral plate) and has a potentially lower perioperative and postoperative morbidity. These results support progression to clinical trials using the anchored spacer as a stand-alone implant.

■ Key Points

- The anchored spacer and each of the plated constructs allowed significantly less motion than the normal intact condition in all directions of loading.
- The anchored spacer provided biomechanical stability equivalent to that of the established anterior fusion technique using an anterior plate plus cage.
- Clinical trials using the anchored spacer as a stand-alone implant should be performed to evaluate this implant *in vivo*.

Acknowledgments

The authors thank the staff of the Spinal Biomechanics Research Laboratory (Barrow Neurologic Institute, Phoenix, AZ) for conducting the testing and Synthes (Synthes GmbH, Switzerland) for supporting the study.

References

1. Kaiser MG, Haid RW Jr, Subach BR, et al. Anterior cervical plating enhances arthrodesis after discectomy and fusion with cortical allograft. *Neurosurgery* 2002;50:229–36.
2. Lee MJ, Bazaz R, Furey CG, et al. Influence of anterior cervical plate design on Dysphagia: a 2-year prospective longitudinal follow-up study. *J Spinal Disord Tech* 2005;18:406–9.
3. Sahjpaul RL. Esophageal perforation from anterior cervical screw migration. *Surg Neurol* 2007;68:205–9.
4. Cain CM, Schleicher P, Gerlach R, et al. A new stand-alone anterior lumbar interbody fusion device: biomechanical comparison with established fixation techniques. *Spine* 2005;30:2631–6.
5. Crawford NR, Brantley AG, Dickman CA, et al. An apparatus for applying pure nonconstraining moments to spine segments in vitro. *Spine* 1995;20: 2097–100.
6. Crawford NR, Dickman CA. Construction of local vertebral coordinate systems using a digitizing probe. Technical note. *Spine* 1997;22:559–63.
7. Crawford NR, Yamaguchi GT, Dickman CA. A new technique for determining 3-D joint angles: the tilt/twist method. *Clin Biomech* 1999;14:153–65.
8. Crawford NR, Peles JD, Dickman CA. The spinal lax zone and neutral zone: measurement techniques and parameter comparisons. *J Spinal Disord* 1998; 11:416–29.
9. Bohler J, Gaudernak T. Anterior plate stabilization for fracture dislocations of the lower cervical spine. *J Trauma* 1980;20:203–5.
10. Coe JD, Vaccaro AR. Complications of anterior cervical plating. In: Clark CR, Benzel EC, Currier BL, et al, eds. *The Cervical Spine*. 4th ed. Philadelphia: Lippincott Williams & Wilkins; 2005.
11. Lowery GL, McDonough RF. The significance of hardware failure in anterior cervical plate fixation. Patients with 2 to 7 year follow-up. *Spine* 1998; 23:181–6.
12. Gu YT, Jia LS, Chen TY. Biomechanical study of a hat type cervical intervertebral fusion cage. *Int Orthop* 2007;31:101–5. Erratum in: *Int Orthop* 2007;31:585.
13. Kandziora F, Ludwig K, Pflugmacher R, et al. Biomechanical comparison of cervical spine interbody fusion cages. *Spine* 2001;26:1850–7.
14. Panjabi MM. The stabilization system of the spine: II. Neutral zone and instability hypothesis. *J Spinal Disord* 1992;5:390–6.
15. Patwardhan AG, Havey RM, Ghanayem AJ, et al. Load-carrying capacity of the human cervical spine in compression is increased under follower load. *Spine* 2000;25:1548–54.

Copyright © Lippincott Williams & Wilkins. Unauthorized reproduction of this article is prohibited.
