# Supplementary material for: Meta-Analysis Comparing Zero-Profile Spacer and Anterior Plate in Anterior Cervical Fusion
Source: PLoS One. 2015 Jun 11;10(6):e0130223. doi: 10.1371/journal.pone.0130223 (PMC4466022; doi:10.1371/journal.pone.0130223)
Supplement: S1 File — (ZIP) [file pone.0130223.s013.zip › S8_ZIP. Fve full-text excluded studies and reasons for exclusion/Excluded Study_4.pdf]

J Neurosurg Spine 9:444–449, 2008

Anterior cervical fusion: a biomechanical comparison of 4 techniques

Laboratory investigation

Fabio Galbusera, M.S.Eng.,[1] Chiara M. Bellini, M.S.Eng.,[1] Francesco Costa, M.D.,[2] Roberto Assietti, M.D.,[3] and Maurizio Fornari, M.D.[2]

[1]LaBS, IRCCS Istituto Ortopedico Galeazzi; [2]Neurosurgery Operative Unit, IRCCS Istituto Ortopedico Galeazzi; and [3]Neurosurgery Operative Unit, Ospedale Fatebenefratelli e Oftalmico, Milan, Italy

Object. Cervical instrumented fusion is currently performed using several fixation methods. In the present paper, the authors compare the following 4 implantation methods: a stand-alone cage, a cage supplemented by an anterior locking plate, a cage supplemented by an anterior dynamic plate, and a dynamic combined plate–cage device.

Methods. Four finite element models of the C4–7 segments were built, each including a different instrumented fixation type at the C5–6 level. A compressive preload of 100 N combined with a pure moment of 2.5 Nm in flexion, extension, right lateral bending, and right axial rotation was applied to the 4 models. The segmental principal ranges of motion and the load shared by the interbody cage were obtained for each simulation.

Results. The stand-alone cage showed the lowest stabilization capability among the 4 configurations investigated, but it was still significant. The cage supplemented by the locking plate was very stiff in all directions. The 2 dynamic plate configurations reduced flexibility in all directions compared with the intact case, but they left significant mobility in the implanted segment. These configurations were able to share a significant part of the load (up to 40% for the combined plate–cage) through the posterior cage. The highest risk of subsidence was obtained with the model of the stand-alone cage.

Conclusions. Noticeable differences in the results were detected for the 4 configurations. The actual clinical relevance of these differences, currently considered not of critical importance, should be investigated by randomized clinical trials. *(DOI: 10.3171/SPI.2008.9.11.444)*

Key Words • anterior plate • cage • cervical fusion • dynamic plate • finite element

Cervical instrumented fusion is currently performed using several fixation methods, such as grafts, stand-alone cages, anterior and lateral mass plating, pedicular screw fixation, and laminar hooks. Anterior plating combined with an interbody device is one of the most common methods.[15,16] Some biomechanical investigations comparing these different approaches are currently available in the literature.[1,4,7,14] Locking versus dynamic plating appears to be a topic of intense research.[2,12] Comparisons between different types of interbody cages have also been documented.[10,12]

This paper is targeted to the biomechanical comparison of the following 4 methods currently used in the treatment of cervical instability: implantation of a stand-alone cage, of a cage supplemented by an anterior locking plate, of a cage supplemented by an anterior dynamic plate, and of a dynamic combined plate–cage device. Segmental ROMs, load sharing between the cage and the plate, and pressures at the endplates were evaluated using finite element models.

Abbreviation used in this paper: ROM = range of motion.

Materials

Four finite element models of the C4–7 segments were built, each including an instrumented fixation at C5–6. All models were based on a validated model of the intact C4–7 segment, described in detail in a previous paper.[6] The material properties assumed for the various components are reported in Table 1. The 4 models were designed to simulate the stage immediately postoperatively and thus did not take into account bone fusion.

TABLE 1
*Mechanical properties of the components of the finite element models**

Material	E (MPa)	Poisson Ratio (ν)
cancellous bone	100	0.3
cortical bone	12,000	0.3
nucleus pulposus	1	0.499
anulus fibrosus, matrix	2.5	0.45
anulus fibrosus, fibers†	500	0.35
titanium	110,000	0.3
PEEK	3.7	0.3

* PEEK = polyetheretherketone.
† There are 5160 fibers/disc with a cross-sectional area mm^2.

A stand-alone rectangular cage with a finned surface was implanted in the first model (Fig. 1a) after removal of the relevant intervertebral disc. The osseous endplates and the uncinate processes were largely preserved to simulate as closely as possible the surgical procedure. The appropriate cage size (anteroposterior length 15 mm, width 12 mm, and height 5–7 mm) was chosen by comparing the resulting model with lateral radiographs obtained in patients who had undergone implantation. A nonbonded contact was created between the superior and inferior surfaces of the cage and the confining vertebral surfaces. The ligaments at the C5–6 level were pretensioned to simulate a surgical distraction of 1 mm. The finned surface design of the cage was simulated by imposing an ideal rough behavior (infinite friction coefficient) to the contact pair, thus preventing slipping but allowing for separation. The cage material was assumed to be polyetheretherketone and modeled as linear elastic isotropic with an elastic modulus of 3.7 GPa.

The second model included the same cage as the first model but was supplemented by an anterior low profile plate fixed to the vertebral bodies with 4 monocortical screws (Fig. 1b). The plate was 27 mm long, 16 mm wide, and 1.5 mm thick. It was assumed to be made of titanium, having an elastic modulus of 110 GPa. A locking plate was considered in this model; to this purpose, shared nodes at the screw–plate interfaces were used, thus not allowing relative motion between the components. The screw–bone interfaces were implemented using a simplified approach. A fully bonded contact based on the multipoint constraint algorithm between the screw surface and the vertebral cortical shell was created for each screw, which had a diameter of 3 mm.

A third model including an interbody cage supplemented by a generic dynamic anterior plate was directly derived from the second model, by introducing nonbonded contact pairs at the screw–plate interfaces. Thus, screws were assumed to be able to slide into the plate holes. The clearance between the screws and the plate was assumed to be 0.1 mm. The inclination of the outer surface of the screw heads was 45° (Fig. 2).

The fourth model consisted of a combined plate–cage device resembling the PCB cage (Scient'X) (Fig. 1c). This device consisted of a 1.2-mm-thick anterior plate integrated with a hollow intradiscal cage, both made of titanium. The cage was the same size as those used in the previous models. As for the stand-alone cage, a nonbonded contact was created between the superior and inferior surfaces of the cage and the relevant vertebral surfaces. The shapes of the contact surfaces on the endplates were assumed to be the same as those of the models with the interbody cage. The contact friction coefficient was assumed to be 0.3, corresponding to a smooth metal–bone interface.[11] The plate was fixed to the vertebrae by using 2 monocortical screws and a fully bonded contact as in the second and third models. No screw locking on the plate was modeled, as was done for the PCB cage, by implementing the same screw–plate interface behavior of the model including the dynamic plate (Fig. 2).

The same boundary and loading conditions were ap-

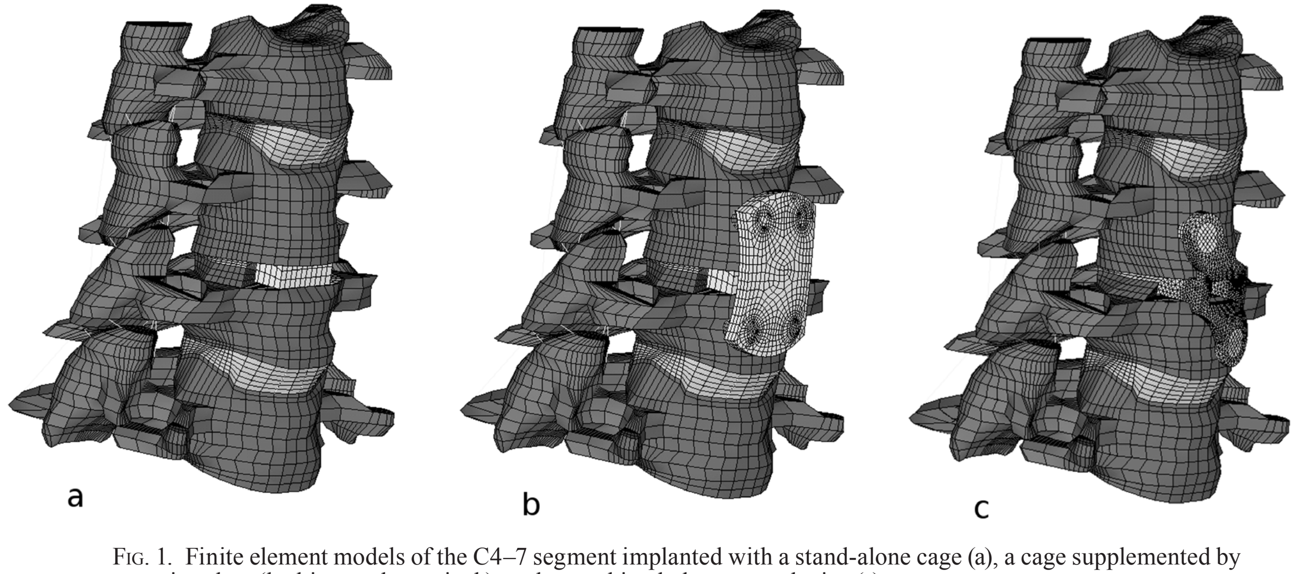

Fig. 1. Finite element models of the C4–7 segment implanted with a stand-alone cage (a), a cage supplemented by an anterior plate (locking or dynamic; b), and a combined plate–cage device (c).

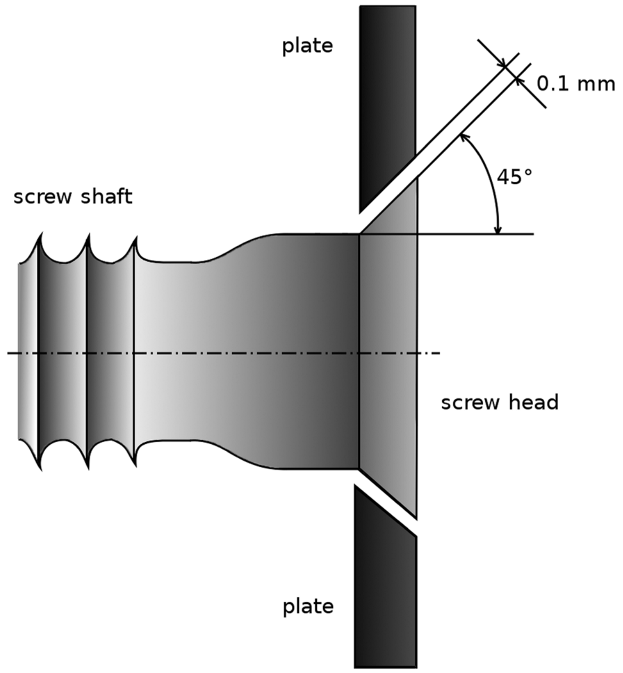

FIG. 2. Illustration demonstrating the geometry of the screw–plate interface in the models including the dynamic plate and the combined plate–cage device. The inclination of the screw head was assumed to be 45°. The clearance between the head and the plate was 0.1 mm.

plied to the 4 models. A compressive preload with 100 N was imposed on the upper endplate of C-4 in all simulations. Four simulations were run for each model by applying a pure moment of 2.5 Nm in different directions (flexion, extension, right lateral bending, and right axial rotation) to the upper endplate of C-4. The segmental principal ROMs were obtained for each simulation at the instrumented and adjacent segments. By analyzing the deformation of the devices, the fraction of load shared by the cage and plate were measured.

Results

Figure 3 shows the segmental ROMs at the C5–6 level obtained with the 4 models, together with the ROMs calculated with the model of the intact segment. The maximum ROMs for all the loading conditions were obtained with the stand-alone cage (up to 55% of the ROM of the intact segment). The cage supplemented by the locking plate was extremely effective in all directions. The 2 dynamic plate configurations were able to reduce the flexibility in all directions compared with the intact case as well as the stand-alone cage, but the plate configurations allowed significant mobility in the implanted segment. The combined plate–cage device allowed greater ROM with respect to the dynamic plate configuration, in particular in flexion and axial rotation.

The load fraction supported by the cage in the different loading conditions of the 4 models is shown in Fig. 4. The locking plate was found to bear a high fraction of the load (> 90%) in all simulations conducted with the relevant model. The dynamic plate configurations shared a greater portion of the load (up to 40% for the combined plate–cage device) through the posterior cage.

Figure 5 depicts a contour plot of the contact pressure at the cage–C6 upper endplate interface in extension. Significantly different peak pressure values for the 4 models were found. The highest pressure was found with the stand-alone cage (2.8 MPa) in the posterior endplate area. The 2 configurations that included a cage supplemented by an anterior plate induced a more uniform pressure distribution on the endplate, with similar peak stress values. Concerning the combined plate–cage model, the contact pressure distribution was asymmetric with respect to the sagittal plane and exhibited a peak pressure value of 1.2 MPa.

Discussion

In the present study, the calculated results are dependent on the actual simulated conditions and should be evaluated only from a comparison point of view. No pretensioning due to the surgical distraction of the surrounding soft tissues, except for the ligaments, was considered in the simulations, which is different from previous ex vivo studies.[17] Neither bone grafts nor newly formed bone was included. This worst-case scenario may lead to an overestimation of the ROM of the fused segment in all simulations. Despite this, a comparison between the different configurations is still possible. Another limitation of the present study pertains to the bone–screw interface, which was modeled as bonded, thus neglecting any possible micromotion. Furthermore, spine degeneration was not considered; thus the anatomy and the material properties were modeled with reference to the healthy condition. Also, the validation process was performed only in the intact spine model,[6] whereas the results obtained with the fixation device models were not validated against experimental data.

Significant differences in ROM were detected among the 4 configurations. The stand-alone cage showed the lowest stabilization capability among the 4 configurations investigated here, in agreement with previous papers,[8,14] but it was still significant. The other configurations induced a smaller ROM in all simulations as in a previous study,[10] although to a different extent due to the static or dynamic nature of the plate.[2] Cage and plate load sharing were also strongly affected by the screw fixation.[12,18] The combined plate–cage device induced the greatest ROM among the 3 plated configurations, in particular in flexion and axial rotation, presumably because of its asymmetrical fixation with only 2 screws.

The stand-alone cage induced a higher value of the peak contact pressure than that of the 3 plate models at the endplate–cage interface in extension. Thus, a higher risk of subsidence may be inferred for this configuration.[8] In the plate configurations, the ability of the plate to share part of the load may play a role in reducing the peak contact pressure. We did not observe any significant differences between the peak values among the 3 plate models, except for the asymmetry in the pressure distri-

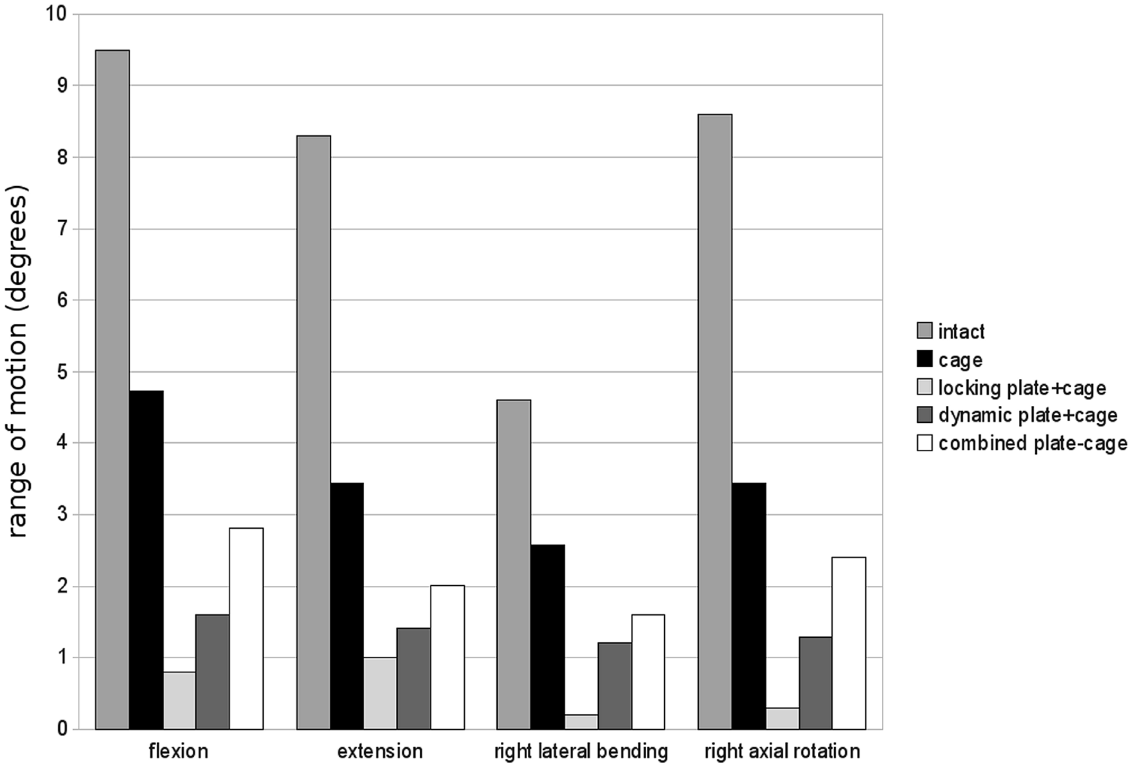

FIG. 3. Graph showing the ROMs calculated with the finite element models in flexion, extension, right lateral bending, and right axial rotation.

bution found for the combined plate–cage device, which is believed to be related to its geometrical asymmetry with respect to the sagittal plane.

The actual relevance of the observed differences in the biomechanics of the stabilized segment should be interpreted by analyzing the clinical results of the different fixation techniques. Globally, the clinical implications of these differences are currently considered not to be of a critical importance. Currently available clinical papers have reported good fusion rates for nonplate, locking, or dynamic plate configurations.[3,13,16] In single-level fusions, the locking or dynamic design of the plate was found not

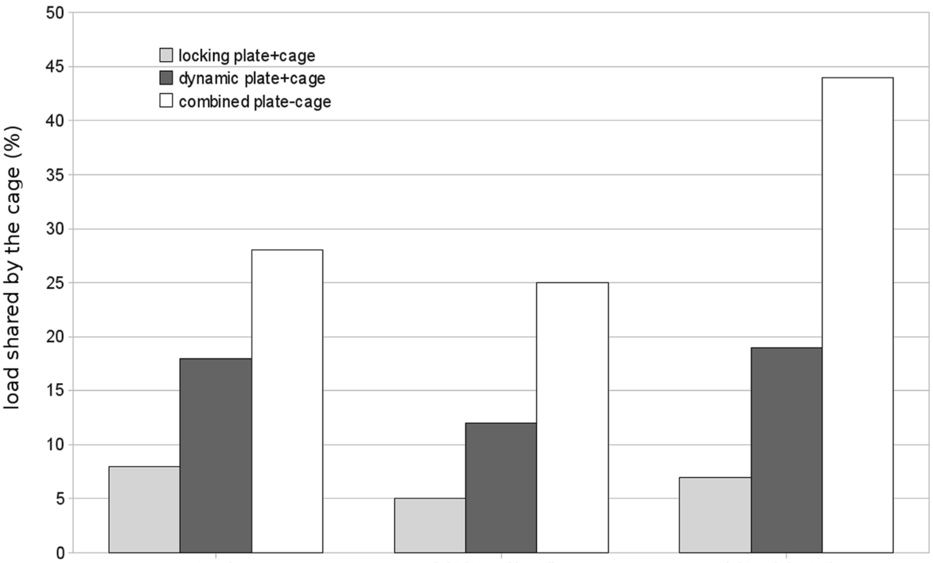

FIG. 4. Graph showing the fraction of load shared by the cage calculated with the models including the cage supplemented with the anterior locking or dynamic plate and the model including the combined plate–cage device.

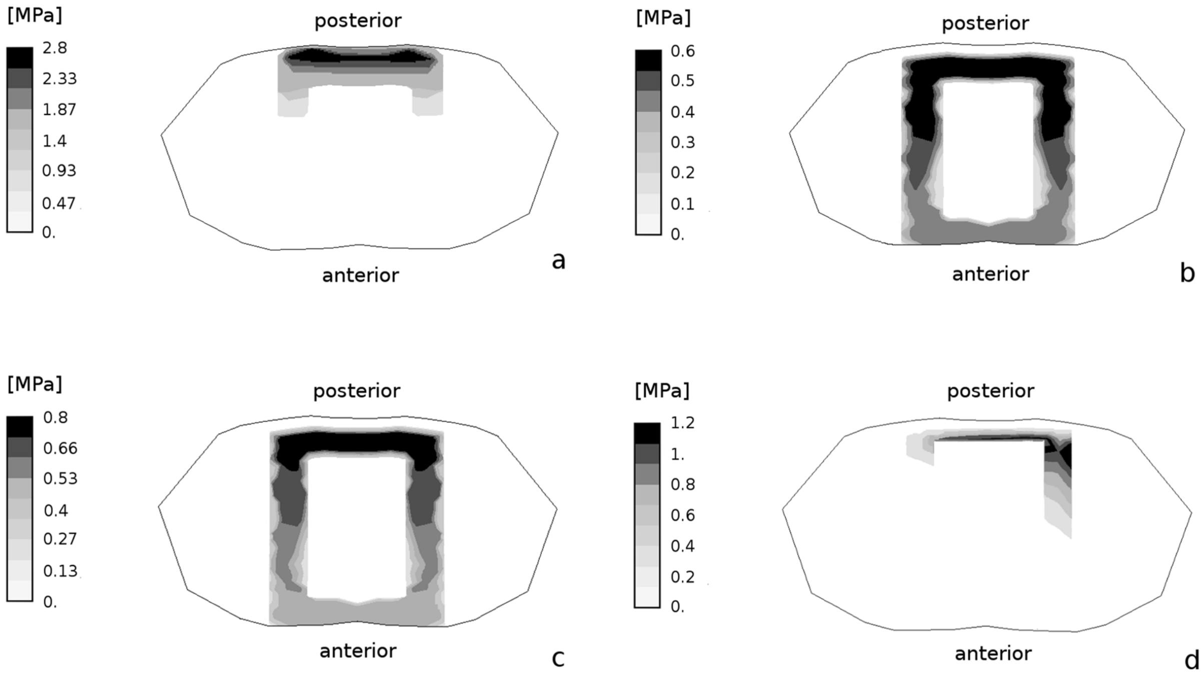

Fig. 5. Images showing the contact pressures in extension at the cage–C6 endplate interface calculated with the models including a stand-alone cage (a), a cage supplemented by an anterior locking plate (b), cage supplemented by an anterior dynamic plate (c), and a combined plate–cage device (d).

to affect the clinical outcome in a study of 66 patients.[9] However, the use of an anterior cervical plate system was reported to increase the fusion rate in a meta-analysis of the literature.[5] In our opinion, a large-scale randomized clinical trial is necessary for the understanding of the real relevance of the biomechanics of the different fixation methods.

Conclusions

Anterior plating provides convenient augmentation to interbody fusion with cages in terms of stabilization potential and reduced risk of cage subsidence. Locking versus dynamic plating induces noticeable differences in the biomechanics of the fused spine, regarding load sharing between the cage and plate and segmental ROM. The actual relevance of these differences, currently considered not of critical importance in single-level fusion, should be investigated with randomized clinical trials.

Disclaimer

The authors report no conflict of interest concerning the materials or methods used in this study or the findings specified in this paper.

References

1. Adams MS, Crawford NR, Chamberlain RH, Sonntag VK, Dickman CA: Biomechanical comparison of anterior cervical plating and combined anterior/lateral mass plating. **Spine J 1:** 166–170, 2001
2. Brodke DS, Klimo P Jr, Bachus KN, Braun JT, Dailey AT: Anterior cervical fixation: analysis of load-sharing and stability with use of static and dynamic plates. **J Bone Joint Surg Am 88:** 1566–1573, 2006
3. Cauthen JC, Theis RP, Allen AT: Anterior cervical fusion: a comparison of cage, dowel and dowel-plate constructs. **Spine J 3:**106–117, 2003
4. Espinoza-Larios A, Ames CP, Chamberlain RH, Sonntag VK, Dickman CA, Crawford NR: Biomechanical comparison of two-level cervical locking posterior screw/rod and hook/rod techniques. **Spine J 7:**194–204, 2007
5. Fraser JF, Härtl R: Anterior approaches to fusion of the cervical spine: a metaanalysis of fusion rates. **J Neurosurg Spine 6:**298–303, 2007
6. Galbusera F, Bellini CM, Raimondi MT, Fornari M, Assietti R: Cervical spine biomechanics following implantation of a disc prosthesis. **Med Eng Phys** [epub ahead of print], 2008
7. Greene DL, Crawford NR, Chamberlain RH, Park SC, Crandall D: Biomechanical comparison of cervical interbody cage versus structural bone graft. **Spine J 3:**262–269, 2003
8. Hakalo J, Pezowicz C, Wronski J, Bedzinski R, Kasprowicz M: Comparative biomechanical study of cervical spine stabilisation by cage alone, cage with plate, or plate-cage: a porcine model. **J Orthop Surg 16:**9–13, 2008
9. Nunley PD, Jawahar A, Kerr EJ III, Cavanaugh DA, Howard C, Brandao SM: Choice of plate may affect outcomes for single versus multilevel ACDF: results of a prospective randomized single-blind trial. **Spine J** [epub ahead of print], 2008
10. Pflugmacher R, Schleicher P, Gumnior S, Turan O, Scholz M, Eindorf T, et al: Biomechanical comparison of bioabsorbable cervical spine interbody fusion cages. **Spine 29:**1717–1722, 2004
11. Rancourt D, Shirazi-Adl A, Drouin G, Paiement G: Friction properties of the interface between porous-surfaced metals

and tibial cancellous bone. **J Biomed Mater Res 24:**1503–1519, 1990
12. Rapoff AJ, Conrad BP, Johnson WM, Cordista A, Rechtine GR: Load sharing in Premier and Zephir anterior cervical plates. **Spine 28:**2648–2650, 2003
13. Samartzis D, Shen FH, Lyon C, Phillips M, Goldberg EJ, An HS: Does rigid instrumentation increase the fusion rate in one-level anterior cervical discectomy and fusion? **Spine J 4:** 636–643, 2004
14. Shimamoto N, Cunningham BW, Dmitriev AE, Minami A, McAfee PC: Biomechanical evaluation of stand-alone interbody fusion cages in the cervical spine. **Spine 26:**E432–E436, 2001
15. Wang JC, McDonough PW, Endow K, Kanim LE, Delamarter RB: The effect of cervical plating on single-level anterior cervical discectomy and fusion. **J Spinal Disord 12:**467–471, 1999
16. Wang M, Gourab K, McGrady LM, Rao RD: Alteration of load sharing of anterior cervical implants with change in cervical sagittal alignment. **Med Eng Phys 30:**768–773, 2008
17. Wilke HJ, Kettler A, Claes L: Primary stabilizing effect of interbody fusion devices for the cervical spine: an in vitro comparison between three different cage types and bone cement. **Eur Spine J** 9:410–416, 2000
18. Yang S, Wang LW: Biomechanical comparison of the stable efficacy of two anterior plating systems. **Clin Biomech (Bristol, Avon)** 18:S59–S66, 2003

Manuscript submitted April 3, 2008.
Accepted August 28, 2008.
Address correspondence to: Fabio Galbusera, M.S.Eng., IRCCS Istituto Ortopedico Galeazzi, via R. Galeazzi 4, 20161 Milan, Italy. email: fabio.galbusera@polimi.it.
