# Supplementary material for: Meta-Analysis Comparing Zero-Profile Spacer and Anterior Plate in Anterior Cervical Fusion
Source: PLoS One. 2015 Jun 11;10(6):e0130223. doi: 10.1371/journal.pone.0130223 (PMC4466022; doi:10.1371/journal.pone.0130223)
Supplement: S1 File — (ZIP) [file pone.0130223.s013.zip › S8_ZIP. Fve full-text excluded studies and reasons for exclusion/Excluded Study_5.pdf]

J Neurosurg Spine 12:342–346, 2010

A unique device, the disc space–fitted distraction device, for anterior cervical discectomy and fusion: early clinical and radiological evaluation

Technical note

Satoshi Tani, M.D., Hiroyasu Nagashima, M.D., Akira Isoshima, M.D., Masahiko Akiyama, M.D., Hiroki Ohashi, M.D., Satoru Tochigi, M.D., and Toshiaki Abe, M.D.

Department of Neurosurgery, Jikei University School of Medicine, Tokyo, Japan

Object. To perform interbody distraction and to obtain spinal curvature correction during anterior cervical discectomy and fusion (ACDF), the authors recently adopted a new stand-alone device, a disc space–fitted distraction device (DFDD). In this preliminary report the authors introduce this unique device and discuss some advantages in terms of short-term clinical and radiological evaluations.

Methods. The most unique aspect of the DFDD is the function of gentle distraction at anterior disc space with maximum lordotic correction of up to 8° while rotating a screw at the front of the device. Additional advantages are related to its configuration such as disc space–matched shape in all dimensions, tapering contour for easy insertion into the disc space, multiple spikes to avoid dislodgment, wider contact area to the vertebral endplate for diminishing sinking process, and sufficient space for accommodation of bone-conductive materials. Twenty-four patients who have been observed more than 12 months after ACDF were involved in this evaluation.

Results. The objective clinical outcome, measured by the Neurological Cervical Spine Scale, was significantly improved. In radiological evaluation, statistically significant improvement compared with preoperative values was noted on the curvature index, C2–7 curvature, and disc angle ($p < 0.01$) throughout the entire postoperative period, up to 12 months. A high fusion rate and remodeling process around the implants were also observed.

Conclusions. The DFDD may have some advantages over other devices—its distraction action, diminished sinking, and early solid bone union resulted in maintaining sufficient correction of the spinal curvature. This corrected spinal curvature may play an important role in preventing progressive adjacent-disc degeneration subsequent to ACDF in the long term. *(DOI: 10.3171/2009.10.SPINE09283)*

Key Words • **anterior cervical discectomy and fusion** • **cage** • **cervical spine** • **spine curvature** • **subsidence**

No devices commercially available have a potent distraction action of the disc space during ACDF. To perform interbody distraction and to obtain spine curvature correction during ACDF, in 2006 we adopted a new stand-alone device, a DFDD (Kisco DIR Co.) (Fig. 1). In this preliminary report we discuss short-term clinical and radiological results associated with the DFDD's positive benefit on the disc space.

Abbreviations used in this paper: ACDF = anterior cervical discectomy and fusion; DFDD = disc space–fitted distraction device; NCSS = Neurological Cervical Spine Scale; VB = vertebral body.

Methods

Description of DFDD

The DFDD is made of pure titanium with dimensions of 12 or 15 mm in width; 5, 6, 7, 8, or 9 mm in height; and 12 mm in depth. The posterior side of the cage is 1 mm lower than the anterior side. The most unique aspect of this device is the function of gentle distraction at the anterior disc space with maximum lordotic correction of up to 8° achieved by rotating a screw in the front of the device (Figs. 1 and 2). Additional advantages are related to its configuration, which matches the disc space shape in 3 dimensions, its tapering contour for easy

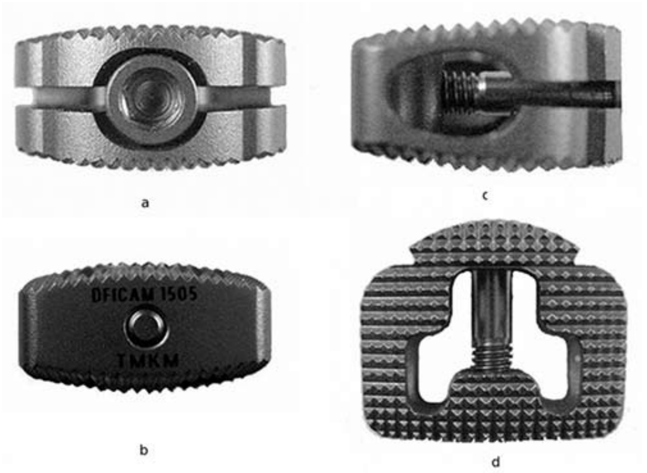

Fig. 1. Photographs showing the configurations of the DFDD: anterior **(a)**, posterior **(b)**, lateral **(c)**, superior **(d)** views.

insertion into the disc space, its multiple spikes to avoid the dislodgment, its wider contact area with the vertebral endplate for diminishing the sinking process, and its sufficient space to accommodate bone-conductive materials. The values associated with the quantity of bone chips or other substitutes without correction and with maximal correction are shown in Table 1.

Patient Population

Twenty-four patients with more than 12 months of post-ACDF follow-up comprise the study population. The objective clinical outcome was measured using the NCSS (Table 2) for neurological evaluation. Subjective neck pain was also scored as none, mild, moderate, or severe. Radiological parameters, as determined on cervical radiographs and reconstructed CT scans, were studied to evaluate whole cervical spine curvature and the disc-level changes at the operated sites. The evaluation of the whole cervical spine curvature included determination of cervical alignment (lordotic, straight, kyphotic, or S shaped) and measurement of curvature index (Ishihara method) (Fig. 3), C2–7 angle (Fig. 4), and C2–7 range of motion. At the treated disc levels we evaluated changes in focal disc angle (Fig. 5), device subsidence, bony fusion, and bony encasement around the device. Positive subsidence was designated when the cage was embedded into the vertebral endplate more than 2 mm, as documented on reconstructed sagittal CT scans. Bone density of the treated VB was not measured preoperatively. Statistical analysis was completed by repeated-measure ANOVA on a personal computer.

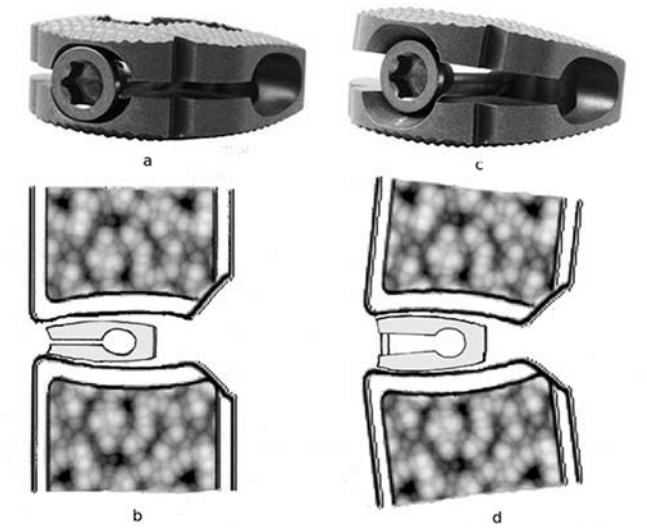

Fig. 2. Photographs showing an anterolateral view of the DFDD before **(a)** and after **(c)** distraction. Schemata showing the predistraction **(b)** and postdistraction **(d)** process of the disc space.

TABLE 1: Association of device size and volume of bone-conducive material, without correction (0°) and with correction (8°)

	Vol (mm^3)	
Device Size (mm)*	Correction Angle 0°	Correction Angle 8°
12 x 5	497	659
12 x 6	580	740
12 x 7	663	823
15 x 5	574	764
15 x 6	672	862
15 x 7	786	976
15 x 8	895	1085
15 x 9	1006	1196

* Size indicates the width by the height.

Operation

After induction of general anesthesia, the patient was placed in the supine position with the neck extended. A standard anteromedian approach was used. All disc tissue, including the herniated disc fragments and osteophytes, was removed under the microscope. Special care was taken not to violate the vertebral endplate. The posterior longitudinal ligament was partially removed to confirm the decompression of neural structures.

Templates of different sizes, varying according to the height of the posterior side of the cage (4, 5, 6, 7, and 8 mm), were introduced to determine the size of DFDD needed. After the cage was filled with autologous cancellous bone harvested from the manubrial bone, the device was inserted into the disc space. The surgeon rotated the screw in front of the cage to distract the anterior part of the device. Rotation was limited to 4 turns (each turn gaining 2°), and the degree of the rotation forces depended on the achievement of correction. After observing the device and adjacent VBs while moving the patient's head in different directions, we closed the surgical wound in the usual manner.

All patients wore a Philadelphia-type orthosis for 3 weeks after surgery.

TABLE 2: Summary of the NCSS*

Score	Function
lower-extremity motor function	
1	total disability: chair-bound or bedridden
2	severe disability: needs support in walking on flat surface
3	moderate disability: difficulty in walking on flat surface & needs support in ascending or descending stairs
4	mild disability: no difficulty in walking on flat surface but mild difficulty in ascending stairs
5	normal: normal walking, w/ or w/o abnormal reflexes
upper-extremity motor function	
1	total disability: unable to perform daily activities
2	severe disability: severe difficulty in daily activities, w/ motor weakness
3	moderate disability: moderate difficulty in daily activities, w/ hand &/or finger clumsiness
4	mild disability: no difficulty in daily activities but mild hand &/ or finger clumsiness
5	normal: normal daily activities, w/ or w/o abnormal reflexes
sensory function &/or pain	
1	severe disturbance: severe difficulty in daily activities, w/ incapacitating sensory disturbance &/or pain
2	moderate disturbance: moderate difficulty in daily activities, w/ sensory disturbance &/or pain
3	mild disturbance: normal daily activities but mild sensory disturbance &/or pain
4	normal: neither sensory disturbance nor pain

* When different levels of neurological function are demonstrated in the right and left extremities, the worse status of the 2 should be recorded.

Results

Anterior cervical discectomy and fusion was performed for the following degenerative diseases: cervical spondylosis (17 cases), cervical herniated disc (6 cases), and cervical malalignment (1 case). The mean preoperative NCSS score of $11.0^{1,8,10–12}$ was significantly improved to $13.8^{12–14}$ 12 months after surgery. Five of 6 patients with preoperative severe neck pain experienced pain relief after surgery; the sixth patient had persistent moderate pain for the following 3 months. No cases of worsening neck pain postoperatively were noted.

In 4 patients of 5 with kyphosis, the curvature was altered to a straight configuration after surgery. In 9 of 12 patients with a straight curvature, the spine improved to a lordotic configuration. In 5 patients with lordotic curvature and in 2 with an S-shaped curvature, the spines remained unchanged. The mean preoperative curvature index of −2.18 improved to 7.92, 7.91, 6.99, and 7.00 at 1, 3, 6, and 12 months, respectively, after surgery (Fig. 3). The mean preoperative C2–7 angle of 4.77° increased to 9.65, 8.76, 9.06, and 9.82° at 1, 3, 6, and 12 months, respectively, after surgery (Fig. 4). The mean preoperative range of motion of 25.8° was decreased by 1.6, 2.1, and 1.5°, respectively, at 3, 6, and 12 months postoperatively. The mean preoperative disc angle of −0.579° was improved by 8.63, 7.95, 7.53, and 7.26°, respectively, at 1, 3, 6, and 12 months after ACDF (Fig. 5). Subsidence of the cage into the VB was observed in 10 (38.5%) of 26 levels; however, more than 3 mm of subsidence was noted in 1 level. No further subsidence was observed after 3 months of observation. Although any biological actions of the grafted bone chips could not be demonstrated on serial CT scans, bone fusion was achieved in 18 levels at 3 months, 21 levels at 6 months, and 25 levels of 26 levels at 12 months. Of all 26 levels, a remodeling process around the device was confirmed in 12 levels at 3 months, 23 levels at 6 months, and all levels at 12 months.

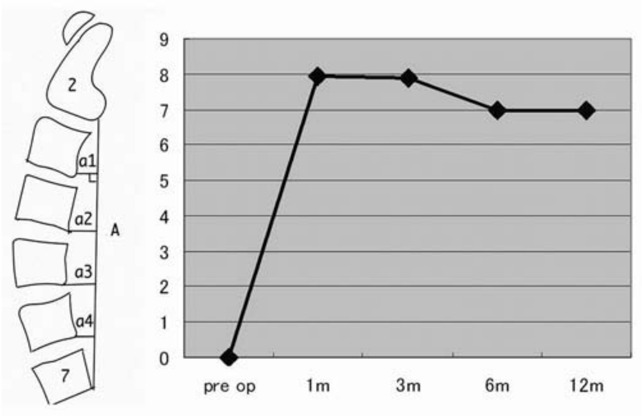

Fig. 3. Left: Schema showing the formula to obtain the curvature index (CI); CI = (a1 + a2 + a3 + a4) × 100 /A. **Right:** Chronological assessment of the mean curvature index demonstrating postoperative improvement as well as its maintenance up to 12 months (m) postoperatively.

Compared with preoperative values, statistically sig-

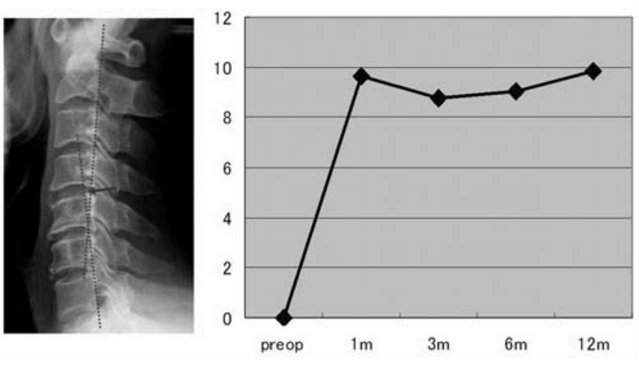

Fig. 4. Left: Lateral radiograph showing the C2–7 angle *(arrow)*. **Right:** Chronological assessment of the C2–7 angle demonstrating postoperative improvement as well as its maintenance up to 12 months postoperatively. Values on the y axis represent degrees.

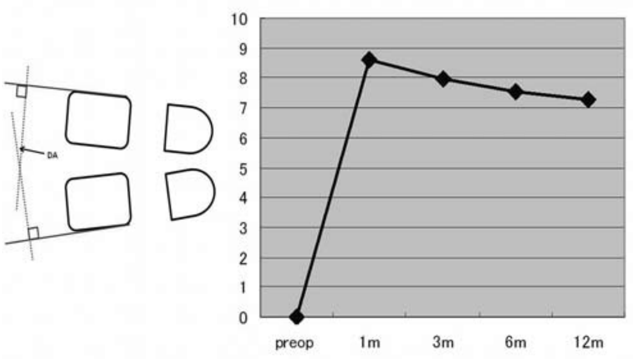

Fig. 5. Left: Schema showing the measurement of disc angle (DA) *(arrow)*. **Right:** Chronological assessment of the mean disc angle demonstrating postoperative improvement as well as its maintenance up to 12 months postoperatively. Values on the y axis represent degrees.

nificant improvement was noted in the curvature index, C2–7 curvature, disc angle ($p < 0.01$) for all follow-up criteria for 12 months.

A single surgical complication of postoperative wound hematoma occurred. After prompt treatment, the patient recovered uneventfully.

Illustrative Case

The images in (Fig. 6) show findings in a representative case.

This 30-year-old woman, who had a 3-year history of progressive tetraparesis associated with dysesthesia in her both hands, was admitted to the hospital with a diagnosis of C5–6 disc herniation with focal kyphotic deformity. Magnetic resonance imaging revealed severe spinal cord compression at C5–6 with high signal cord area on T2-weighted image. The patient underwent ACDF in which the DFDD was placed, and we noted some recovery in sensorimotor disturbance in her extremities. Three months after surgery, her preoperative kyphosis improved to a straightened configuration, and dynamic cervical radiography seemed to reveal fusion at the surgically treated disc level. The improved radiographic parameters including curvature index, C2–7 angle, disc height, and disc angle, and they remained unchanged during a 2-year

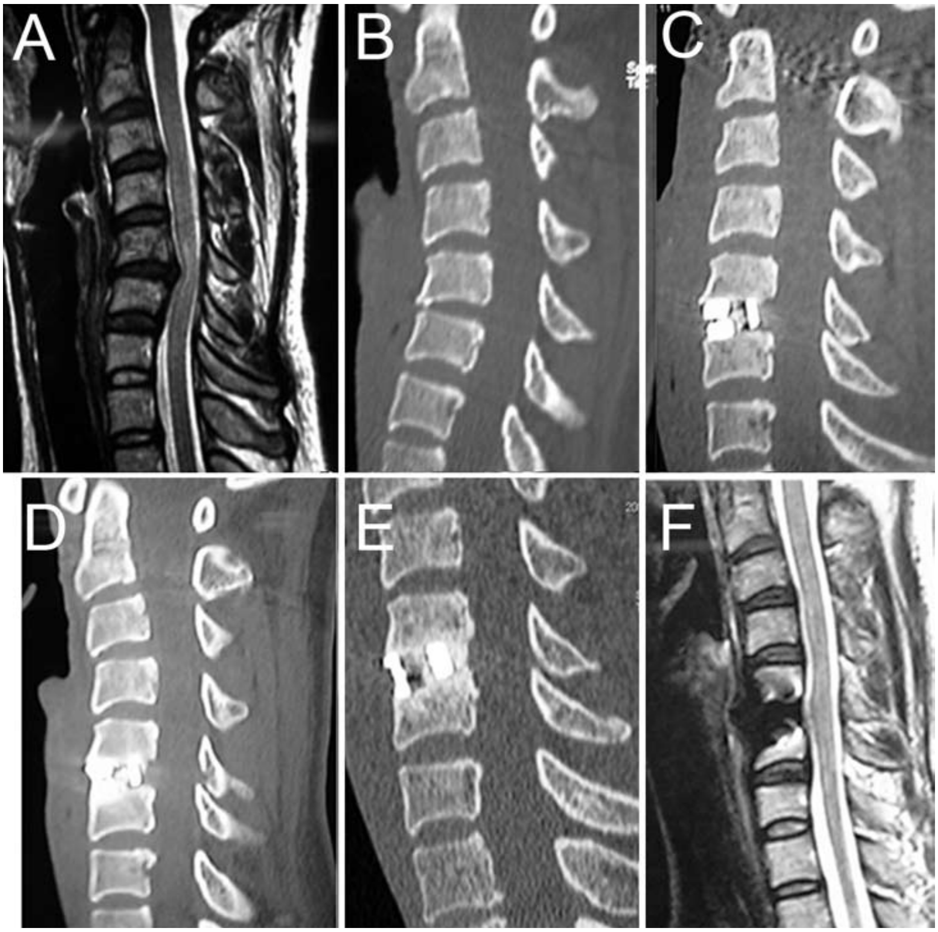

Fig. 6. A: Preoperative T2-weighted MR image showing a long T2 lesion in the cervical cord. **B:** Preoperative sagittal CT reconstruction revealing significant kyphosis. **C:** Reconstructed CT scan 1 month after surgery showing corrected spinal curvature. **D:** Reconstructed CT scan 3 months after surgery demonstrating maintenance of the corrected spinal curvature. **E:** Reconstructed CT scan 12 months after surgery demonstrating the remodeling process around the device. **F:** Postoperative T2-weighted MR image obtained 12 months after surgery.

follow-up period. Reconstructed CT scans demonstrated a remodeling process around the DFDD without significant subsidence.

Discussion

No commercially available devices have an effective distraction action; however, the DFDD, easily inserted in the targeted disc space, can achieve gentle distraction at the anterior disc space.

Surgical outcomes were found to be excellent on neurological and radiological evaluations. We did not observe any cases of worsening neck pain after surgery resulting from disc space distraction. The DFDD seems to produce some correction of the cervical spine curvature due to its unique capacity for gentle distraction. The mild distraction force, configured disc space fitting, and the multiple spikes may play a role in the initial efficient fixation and excellent fusion rate. The DFDD has a potential to achieve bone conduction through its wide window from the vertebral endplate, although the destiny of the grafted bone chips could not be delineated on serial CT scans. Because of the DFDD's mild sinking into the vertebral endplate, the corrected spinal curvature at 1 month seemed to be well maintained up to 12 month after surgery. This maintenance of corrected spinal curvature seems to correlate well to previous reports in which corrected spinal curvature were shown to be stabilized within 1–6 months after surgery.[16]

In relation to implant configuration, more prominent subsidence has been reported with cylindrical cages than with box-type cages.[4,12] The precise reason for this prominent subsidence has not been discovered, but violation of the endplate during disc space preparation for the cage and decreased cylindrical cage–VB interface may play an important role in the prominent subsidence.[1,7,9] The DFDD was not associated with any significant difference in the subsidence rates compared with commercially available box-type cages discussed in the Japanese literature.[7,16] Furthermore, smoking, treatment of lower-level discs, multilevel fusion, malalignment conditions such as kyphosis or a straight spine, and hypermobility at the operated disc level have been reported as risk factors for subsidence,[2,3,7,11,14,15] but no statistically significant risk factor has been established.

Long-term results associated with frequent reoperations following ACDF may be related to adjacent disc level degeneration that could be influenced by an abnormal spinal curvature. Maintaining appropriate spinal curvature may play an important role in the prevention of progressive adjacent-disc degeneration after ACDF in the long term.[5,6,8,10,13]

Conclusions

Although this is a preliminary report introducing a new device, the DFDD for ACDF, the clinical properties of the device—distraction, mild sinking process, and early compact bone union—may have some advantages in maintaining sufficient correction of spinal curvature.

Disclosure

The authors report no conflict of interest concerning the materials or methods used in this study or the findings specified in this paper.

References

1. Barsa P, Suchomel P: Factors affecting sagittal malalignment due to cage subsidence in standalone cage assisted anterior cervical fusion. **Eur Spine J 16:**1395–1400, 2007
2. Bartels RH, Donk RD, Feuth T: Subsidence of stand-alone cervical carbon fiber cages. **Neurosurgery 58:**502–508, 2006
3. Cauthen JC, Theis RP, Allen AT: Anterior cervical fusion: a comparison of cage, dowel and dowel-plate constructs. **Spine J 3:**106–117, 2003
4. Cho DY, Liau WR, Lee WY, Liu JT, Chiu CL, Sheu PC: Preliminary experience using a polyethertherketone (PEEK) cage in the treatment of cervical disc disease. **Neurosurgery 51:**1343–1350, 2002
5. Edwards CC II, Heller JG, Murakami H: Corpectomy versus laminoplasty for multilevel cervical myelopathy: an independent matched-cohort analysis. **Spine 27:**1168–1175, 2002
6. Hacker RJ: A randomized prospective study of an anterior cervical interbody fusion device with a minimum of 2 years of follow-up results. **J Neurosurg 93 (2 Suppl):**222–226, 2000
7. Horikoshi T, Uchida M, Watanabe A, Kinouchi H: Factors affecting Titanium cage subsidence after anterior fixation of the cervical spine. **Spinal Surgery 22:**17–22, 2008 (Jpn)
8. Katsuura A, Hukuda S, Saruhashi Y, Mori K, Imai S: Kyphotic malalignment after anterior cervical fusion is one of the factors promoting the degenerative process in adjacent intervertebral levels. **Eur Spine J 10:**320–324, 2001
9. Matgé G: Cervical cage fusion with 5 different implants: 250 cases. **Acta Neurochir (Wien) 144:**539–550, 2002
10. Rajshekhar V, Arunkumar MJ, Kumar SS: Changes in cervical spine curvature after uninstrumented one- and two-level corpectomy in patients with spondylotic myelopathy. **Neurosurgery 52:**799–805, 2003
11. Schmieder K, Wolzik-Grossmann M, Pechlivanis I, Engelhardt M, Scholz M, Harders A: Subsidence of the wing titanium cage after anterior cervical interbody fusion: 2-year follow-up study. **J Neurosurg Spine 4:**447–453, 2006
12. Tancredi A, Agrillo A, Delfini R, Fiume D, Frati A, Rinaldi A: Use of carbon fiber cages for treatment of cervical myeloradiculopathies. **Surg Neurol 61:**221–226, 2004
13. Türeyen K: Disc height loss after anterior cervical microdiscectomy with titanium intervertebral cage fusion. **Acta Neurochir (Wien) 145:**565–570, 2003
14. van Jonbergen HP, Spruit M, Anderson PG, Pavlov PW: Anterior cervical interbody fusion with a titanium box cage: early radiological assessment of fusion and subsidence. **Spine J 5:**645–649, 2005
15. Wilke HJ, Kettler A, Goetz C, Claes L: Subsidence resulting from simulated postoperative neck movements: an in vitro investigation with a new cervical fusion cage. **Spine 25:**2762–2770, 2000
16. Yunoki M, Hirashita K, Gohda Y, Yoshino K, Fujimoto S: Radiographic evaluation of anterior cervical fusion with a box-design titanium cage. **Spinal Surgery 20:**233–240, 2006

Manuscript submitted March 31, 2009.

Accepted October 23, 2009.

Address correspondence to: Satoshi Tani, M.D., 3-25-8, Nishishinbashi, Minatoku, Tokyo, Japan 105-8461. email: tani@jikei.ac.jp.
