# Supplementary material for: Meta-Analysis Comparing Zero-Profile Spacer and Anterior Plate in Anterior Cervical Fusion
Source: PLoS One. 2015 Jun 11;10(6):e0130223. doi: 10.1371/journal.pone.0130223 (PMC4466022; doi:10.1371/journal.pone.0130223)
Supplement: S1 File — (ZIP) [file pone.0130223.s013.zip › S8_ZIP. Fve full-text excluded studies and reasons for exclusion/The reasons for exclusion.docx]

**The reasons for exclusion**

**(1) Title:** Zero-P: a new zero-profile cage-plate device for single and multilevel ACDF. A single institution series with four years maximum follow-up and review of the literature on zero-profile devices.

**Reason for exclusion**: This article was pubished on European Spine Journal by Dr. Barbagallo et al. in 2013. (DOI: 10.1007/s00586-013-3005-0) It presented a series of patients treated with ACDF only with Zero-P device. No patients were implanted with anterior plate. Thus this study was excluded.

**(2) Title:** Stand-alone cervical cages versus anterior cervical plate in 2-level cervical anterior interbody fusion patients: Clinical outcomes and radiologic changes.

**Reason for exclusion**: This article was pubished on Journal of Spinal Disorders and Techniques by Dr. Jae Keun Oh et al. in 2013.(DOI: 10.1097/BSD.0b013e31824c7d22) This retrospective study analyzed the fusion rate of two kinds of procedures (ACDF with stand-alone cage VS ACDF with stand-alone cage and anterior plate). No patients were treated with Zero-P device. Thus this study was excluded.

**(3) Title:** A New Stand-Alone Cervical Anterior Interbody Fusion Device Biomechanical Comparison With Established Anterior Cervical Fixation Devices.

**Reason for exclusion**: This article was pubished on Spine by Dr. Matti Scholz et al. in 2009.(DOI: 10.1097/BRS.0b013e31818ff9c4) It was the first study of Zero-P. Obviously, judging from the title it was a biomechanical study on human cervical spine cadaver. Thus this study was excluded.

**(4) Title:** Anterior cervical fusion: a biomechanical comparison of 4 techniques. Laboratory investigation.

**Reason for exclusion**: This article was pubished on Journal of Neurosurgery: Spine by Galbusera et al. in 2008.(DOI: 10.3171/spi.2008.9.11.444) This study was finite element study. Thus it was excluded.

(5) **Title:** A unique device, the disc space-fitted distraction device, for anterior cervical discectomy and fusion: early clinical and radiological evaluation Technical note

**Reason for exclusion**: This article was pubished on Journal of Neurosurgery: Spine by SatoShi Tanietal et al. in 2010.(DOI: 10.3171/2009.10.spine09283) This study was a technical report. Thus it was excluded.
